# Molecular Evolution of Multiple-Level Control of Heme Biosynthesis Pathway in Animal Kingdom

**DOI:** 10.1371/journal.pone.0086718

**Published:** 2014-01-28

**Authors:** Wen-Shyong Tzou, Ying Chu, Tzung-Yi Lin, Chin-Hwa Hu, Tun-Wen Pai, Hsin-Fu Liu, Han-Jia Lin, Ildeofonso Cases, Ana Rojas, Mayka Sanchez, Zong-Ye You, Ming-Wei Hsu

**Affiliations:** 1 Department of Life Sciences, National Taiwan Ocean University, Keelung, Taiwan; 2 Institute of Bioscience and Biotechnology, National Taiwan Ocean University, Keelung, Taiwan; 3 Department of Computer Science and Engineering, National Taiwan Ocean University, Keelung, Taiwan; 4 Department of Medical Research, Mackay Memorial Hospital, Taipei, Taiwan; 5 Computational Cell Biology Group, Institute of Predictive and Personalized Medicine of Cancer (IMPPC), Barcelona, Spain; 6 Cancer and Iron Group, Institute of Predictive and Personalized Medicine of Cancer (IMPPC), Barcelona, Spain; CINVESTAV-IPN, Mexico

## Abstract

Adaptation of enzymes in a metabolic pathway can occur not only through changes in amino acid sequences but also through variations in transcriptional activation, mRNA splicing and mRNA translation. The heme biosynthesis pathway, a linear pathway comprised of eight consecutive enzymes in animals, provides researchers with ample information for multiple types of evolutionary analyses performed with respect to the position of each enzyme in the pathway. Through bioinformatics analysis, we found that the protein-coding sequences of all enzymes in this pathway are under strong purifying selection, from cnidarians to mammals. However, loose evolutionary constraints are observed for enzymes in which self-catalysis occurs. Through comparative genomics, we found that in animals, the first intron of the enzyme-encoding genes has been co-opted for transcriptional activation of the genes in this pathway. Organisms sense the cellular content of iron, and through iron-responsive elements in the 5′ untranslated regions of mRNAs and the intron-exon boundary regions of pathway genes, translational inhibition and exon choice in enzymes may be enabled, respectively. Pathway product (heme)-mediated negative feedback control can affect the transport of pathway enzymes into the mitochondria as well as the ubiquitin-mediated stability of enzymes. Remarkably, the positions of these controls on pathway activity are not ubiquitous but are biased towards the enzymes in the upstream portion of the pathway. We revealed that multiple-level controls on the activity of the heme biosynthesis pathway depend on the linear depth of the enzymes in the pathway, indicating a new strategy for discovering the molecular constraints that shape the evolution of a metabolic pathway.

## Introduction

Molecular evolution has recently been a popular area of investigation, and through the advancement of technology and the maturation of analysis methods, this field continues to spawn important insights into the evolutionary processes affecting genes. None of these genes or their encoded proteins exists in isolation, and the products of genes construct the metabolic pathways and networks underlying the cellular and metabolic processes of organisms. As an increasing number of studies are describing the rates of protein and pathway evolution over evolutionary time, there are more opportunities to clarify the patterns and principles of natural selection acting on the pathways involved in the metabolic networks of organisms.

Research focusing on the effects of the organization of pathways on the strength of selection acting on individual proteins in these pathways has revealed various evolutionary patterns among proteins at different positions in a pathway. Important research has been conducted with respect to the plant anthocyanin biosynthetic pathway [1]–[3], terpenoid biosynthesis pathway [4], starch biosynthesis pathway [5], [6], gibberellin biosynthesis pathway [7] and carotenoid biosynthesis pathway [8] as well as lateral line innovation in teleosts [9], the glucosinolate pathway in *Arabidopsis thaliana* [10] and the primate N-glycosylation pathway [11]. It has been demonstrated that the pleiotropic genes in the upstream portions of pathways or those found at branch points in a network are subject to stronger selective constraints [12]. On the other hand, selection is relaxed in the downstream enzymes, and nonsynonymous substitution rates as well as *d_N_/d_S_* ratios are higher in these pathway components.

**Figure 1:**
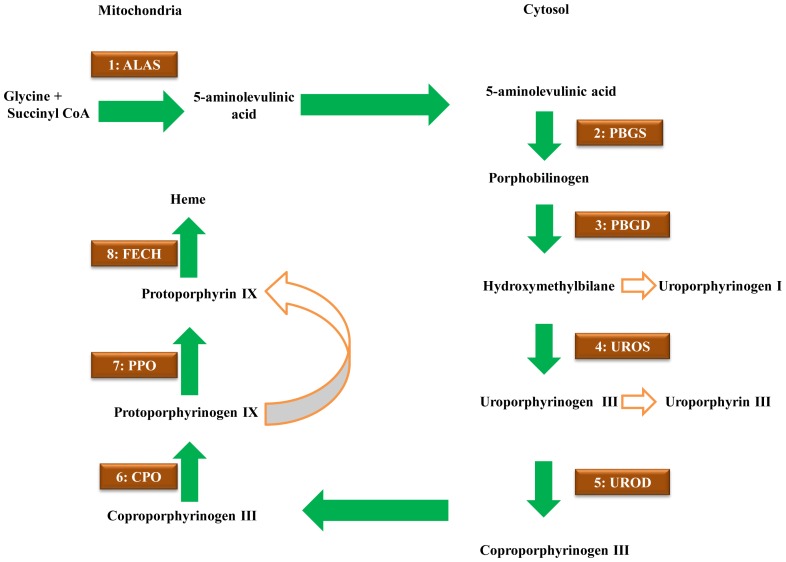
Heme biosynthesis pathway in animals. The substrate and product are indicated for each enzyme and the subcellular localization of each enzyme is also shown (cytosol or mitochondria). Each enzyme is coded from one to eight according to the linear order of the pathway. Also shown are the processes by which hydroxymethylbilane, the substrate of UROS, can be non-enzymatically cyclized to form uroporphyrinogen I, leading to uroporphyrin I or coproporphyrin I, and the process by which uroporphyrinogen III, the product of UROS, can be auto-oxidized to form uroporphyrin III. Protoporphyrinogen, the substrate of PPO, can be auto-oxidized to form protoporphyrin.

The heme biosynthesis pathway is an appropriate system not only for comparing the evolutionary rates of genes according to their position or pathway reticulation but also for studying functional motifs that may play a role at several levels of gene regulation. Heme acts as an essential cofactor for cytochromes, oxidases, peroxidases, catalases, hemoglobin and myoglobin in organisms. Heme acts as an iron-chelating tetrapyrrole and is composed of a complex macrocycle containing four pyrrolic rings connected by methine bridges in cyclic form. Heme also plays multiple regulatory roles, including microRNA processing, ion channel functions, circadian rhythms, mitochondrial targeting, translational regulation and protein degradation [13]–[16]. The heme biosynthesis pathway is an especially well-characterized and important pathway for erythroid production in animals, and malfunctions in heme biosynthesis result in several types of porphyrias because of the accumulation of toxic tetrapyrrole intermediates [17]. The heme biosynthesis pathway of animals is comprised of eight consecutive genes: 5-aminolevulinic acid synthase (*ALAS*), porphobilinogen synthase (*PBGS*), porphobilinogen deaminase (*PBGD*), uroporphyrinogen III synthase (*UROS*), uroporphyrinogen III decarboxylase (*UROD*), coproporphyrinogen III oxidase (*CPO*), protoporphyrinogen IX oxidase (*PPO*) and ferrochelatase (*FECH*), from positions one to eight (Figure 1). In animals, the formation of heme occurs in the mitochondria and cytosol. Heme biosynthesis begins with the formation of 5-aminolevulinic acid. In the Shemin pathway of animals, ALAS catalyzes a single-step reaction to condense glycine and succinyl-CoA into ALA, with the elimination of CO_2_. In vertebrates, the housekeeping isoforms of ALAS1 are expressed in all cell types, and ALAS2 is expressed in erythroid cells (eALAS) at a very high level. Subsequently, PBGS converts two 5-aminolevulinic acids into porphobilinogen, and the addition of four PBG substrates to a dipyrrole by PBGD results in the production of pre-uroporphyrinogen, also known as hydroxymethylbilane. A circularization reaction performed by UROS generates uroporphyrinogen III. The modification of side chains on the cyclic intermediates sequentially mediated by UROD, CPO and PPO results in protoporphyrin IX, which is subsequently chelated with ferrous iron by FECH (see reviews in [14], [18]).

ALAS1 and ALAS2 are derived from gene duplication. A phylogenetic analysis of ALAS suggested that the relevant gene duplication event took place before the divergence of hagfish from the deuterostome line leading to vertebrates [19]. In extant vertebrate species, ALAS1 and ALAS2 are paralogs, and their amino acid sequences are highly similar to each other.

Regulation of the genes encoding the eight enzymes of the heme biosynthesis pathway can occur at the transcriptional, translational and post-translational levels. At the transcriptional level, multiple erythroid-specific factors have been observed to be involved in the transcriptional activation of several genes that participate in heme biosynthesis and erythropoiesis. Among these transcription factors, genomic DNA-binding activity and the conserved binding sites of KLF1, GATA1 and TAL1 have been studied in humans and mice [20]. The binding sites of KLF1 are located within the intergenic regions or introns (particularly the first intron) of genes encoding the components of the heme biosynthesis pathway. In a de novo motif study, GATA1 and TAL1 were hypothesized to complex with KLF1 in a small subset of erythroid *cis*-regulatory modules [21]. The majority of the GATA1-binding sites that mediate the activation of gene expression are close to the transcription start site, within either the first intron or the proximal 5′ flanking region [22]. TAL1-binding sites for the eight genes of the heme biosynthesis pathway are detected in either proximal promoter or intronic regions [23]. A housekeeping promoter utilized in all tissue types exists in ALAS1, PBGS, PBGD, and UROS. However, for ALAS2, PBGS, PBGD and UROS, erythroid-specific promoters drive gene expression. More importantly, the alternative promoters found in PBGS, PBGD and UROS are located in intron 1, and an alternative splicing event is required for the transcript generated from the housekeeping promoters and erythroid-specific promoters [24]. It has also been established that DNase-hypersensitive sites usually serve as sites for conserved and cell-type specific transcription factor binding and histone modification [25]–[27]. Combined analysis of intron 1 sequences and DNase-hypersensitive sites will shed light on the regulatory potential of the corresponding *cis*-elements and the evolution of the heme biosynthesis pathway.

At the translational level, IRE-binding protein (IRP) interacts with the iron-responsive element (IRE) located in the 5′ untranslated regions (5′UTRs) of ALAS2 and ferritin mRNA to inhibit protein translation and in the 3′ untranslated region (3′UTR) of TfR1 to stabilize mRNA. During iron repletion, iron-sulfur clusters can abolish the IRE-binding ability of IRP1, and the F-box protein FBXL5 recognizes IRP2 and targets it for degradation by E3 ligases. Both mechanisms offer a link between iron availability and heme synthesis [28]–[30]. At the post-translational level, the binding of heme to the heme-regulatory motif (HRM) in the mature ALAS1 peptide blocks mitochondrial import and results in end-product inhibition [19], [31]–[33].

The objectives of this study are to investigate the molecular fingerprints underlying the adaptation of the eight genes encoding the enzymes of the heme biosynthesis pathway in Kingdom Metazoa and, particularly, how these adaptations correlate with the positions of the gene products (enzymes) in the pathway. First, through the analysis of the evolutionary constraints on the protein-coding sequences, we reveal strong purifying selection on the protein sequences and clade-specific adaptations in teleosts and arthropods. We also demonstrate that the first introns of these genes in vertebrates play a role in their erythroid-specific transactivation, highlighting the emergence of erythrocytes in animals. Then, we show that pathway product (heme) feedback control is widely utilized in this pathway. The evolutionarily conserved motifs that enable this control include HRMs found not only in ALAS but also in PBGS. We also discover IREs within 5′UTRs and exon-intron boundaries, raising the possibility of regulation of both translation and splicing choice, respectively. We conclude by summarizing the evolution of multifarious controls on the heme biosynthesis pathway.

## Results

### Relationship between Selection Pressure and Pathway Position

**Figure 2:**
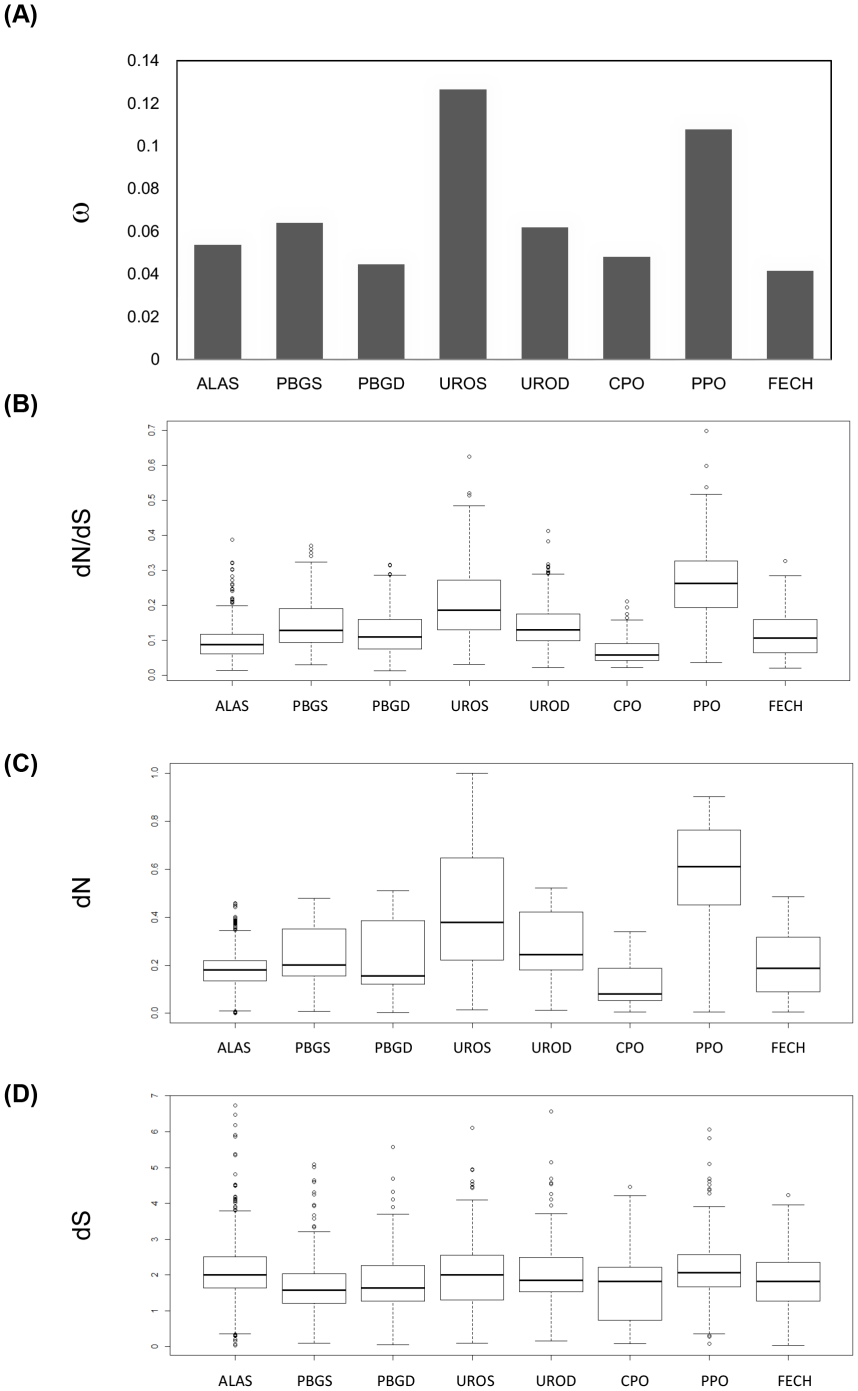
Selection pressure on heme biosynthesis genes in animals. ω values (*d_N_/d_S_*) were estimated with the M0 model for the eight heme biosynthesis genes in animals (A). The distribution of ω values (B), the nonsynonymous substitution rate, *d_N_* (C), and the synonymous substitution rate, *d_S_* (D). The order of genes follows the linear order of their pathway positions (Figure 1).

Based on the M0 model, assuming a constant evolutionary rate (nonsynonymous versus synonymous rate, *d_N_/d_S_*, ω) for all branches and all codons of the eight genes encoding the enzymes of the metazoan heme biosynthesis pathway, the ω values vary from 0.041 (FECH) to 0.127 (UROS), providing evidence that the sequences of the coding regions of these genes are under negative selection (Figure 2A). We also evaluated the M1a model, which assumes that there are two groups of codons, subject to purifying selection and neutral evolution, in contrast to the M0 model. We found that the M1a model significantly improves the model (*p*<0.001) and that a large fraction of the codons are under purifying selection (>91% in all genes, except for PPO, where the obtained value was 79%). Notably, the ω values at positions four (UROS) and seven (PPO) were highest (0.116 and 0.105, respectively) among the genes of the heme biosynthesis pathway. (Table S1).

To determine whether the variation in ω values among the genes was statistically significant and showed a relationship with their positions in the pathway, we conducted several tests on the *d_N_*, *d_S_* and ω values obtained for each gene (Figure 2B, 2C, 2D). First, we determined whether the distributions of the *d_N_*, *d_S_* and ω values were correlated. We found that the distributions of the *d_N_*, *d_S_* and ω values were not correlated for each gene (Kruskal-Wallis rank sum test, *p*<0.0001). Second, the ω values of genes located at positions four (UROS) and seven (PPO) were shown to be significantly higher than for the genes located at other positions (Wilcoxon rank sum test, P<0.0001). Third, to determine the significance of the variations in ω values among the genes, we conducted multiple comparisons of the ω values for each gene pair (see Methods). Three groups were established: group 1 (low ω values), consisting of FECH, PBGD and CPO; group 2 (intermediate ω values), consisting of ALAS, UROD and PBGS; and group 3 (high ω values), consisting of PPO and UROS (Table 1). Fourth, we determined whether the *d_N_*, *d_S_* and ω values were correlated with the pathway positions of the eight enzymes of the heme biosynthesis pathway. It was found that the ω values were positively correlated with the pathway positions of the enzymes (Kendall’s correlation test: tau = 0.2202, *P*<0.0001), as were the *d_N_* values (Kendall’s correlation test: tau = 0.1625, *P*<0.0001). However, the *d_S_* values were not correlated with pathway position (Kendall’s correlation test: tau = −0.0372, *P = *0.0243).

**Table 1:** Comparison of ω values among eight genes to test the significance of the ω variations among genes.

		Group 1			Group 2			Group 3	
		FECH	PBGD	CPO	ALAS	UROD	PBGS	PPO	UROS
	FECH				**	*	**	**	**
Group 1	PBGD				*		**	**	**
	CPO				*		*	**	**
	ALAS							**	**
Group 2	UROD	**	*					**	**
	PBGS	**	**	*	*			**	**
Group 3	PPO	**	**	**	**	**	**		
	UROS	**	**	**	**	**	**		

For each comparison, a pair of genes was chosen. ω value of the gene in the row is constrained to the average value of omega values from the genes in the rows and columns. The difference in the likelihood between the null model M0 and the constrained model was obtained. The significance level is labeled * if *p*<0.05 and ** if *p*<0.01.

In conclusion, the variations in ω and *d_N_* values were shown to be correlated with the pathway positions of the eight enzymes of the heme biosynthesis pathway.

### Amino Acid Residues under Positive Selection

When the genes of the heme biosynthesis pathway are observed to be under strong purifying selection, it is interesting to examine whether there are amino acids in certain lineages that experience positive selection. Due to limitations regarding the number of sequences collected, we focused on the protein sequences of the mammal, teleost and arthropod subgroups. Of the eight enzymes involved in heme biosynthesis, only ALAS2 from teleosts, PBGS from arthropods and UROD from teleosts showed positively selected residues in the branch-site model (*p* value <0.05) (Table 2). In ALAS2 from teleosts, five sites were found to be positively selected (model A/A1, *p*<2.3e-10) (BEB >0.978) (Table 3). Notably, while the amino acid detected in teleost ALAS2 at position 204 is I/M/T, the amino acid E is present at this position in all other vertebrate ALAS1 proteins. We mapped this amino acid in the crystal structure of the protein and found that it is positioned at the ALAS dimer interface. The amino acid R located at position 353 in teleost ALAS2 is also interesting because a K residue is found at this position in all other vertebrate ALAS1, ALAS2 and ALAS proteins. The crystal structure of ALAS indicates that this amino acid forms a hydrogen bond with the ribose-O3’ of the substrate succinyl-CoA [34] (Figure S1A).

**Table 2:** Selection pressure of genes of heme-biosynthesis pathway by employing the branch model.

Model	Model	Estimates of parameters	2ΔL^a^	*p* Value^b^
ALAS+ALAS1+ALAS2	M0: one ratio	ω = 0.05356		
Teleost ALAS2 branch	two ratios	ω0 = 0.05323, ω = 8.04979	10.087074	1.49E-03
PBGS	M0: one ratio	ω = 0.06398		
Arthropod branch	two ratios	ω0 = 0.06398, ω = 999.000	4.478516	3.43E-02
UROD	M0: one ratio	ω = 0.06194		
Teleost branch	two ratios	ω0 = 0.06135, ω = 13.69790	6.01671	1.42E-02

a Twice the difference between the log likelihood of M0 (one ratio) and two ratio model.

b *p*(χ2) of the likelihood ratio test.

**Table 3:** Positive selection in the different lineages of genes of heme biosynthesis pathway.

Foreground branches	2ΔL^a^	*p* Value^b^	Estimates of the parameters in the modified model A^c^	Positively selected sites^d^
Teleost ALAS2	40.20213	2.29E-10	p0 = 0.86464, p1 = 0.07816, p2a = 0.05246,	204E, 243K, 352P, 353K,
			p2b = 0.00474, ω0 = 0.04595, ω2 = 999.0	431G
Arthropod PBGS	23.97615	9.75E-07	p0 = 0.86020, p1 = 0.03447, p2a = 0.10127,	11Y, 106H, 162C, 195S,
			p2b = 0.00406, ω0 = 0.06383, ω2 = 999.0	267K, 274A, 314I
Teleost UROD	25.60371	4.19E-07	p0 = 0.87046, p1 = 0.07295, p2a = 0.05222,	136Q, 174M, 290K, 297K,
			p2b = 0.00438, ω0 = 0.06761, ω2 = 999.0	300T, 316E, 349H

a Twice the difference between the log likelihood of M0 (one ratio) and two ratio model.

b *p*-value *p*(χ2) of the likelihood ratio test.

c For the branch-site model A, the following four classes are demarcated to each amino acid: class 0 with 0< ω0<1 in all branches; class 1 with ω1 = 1 in all branches; class 2a with foreground ω2>1 but background 0< ω0<1; and class 2b with foreground ω2>1 but background ω1 = 1. p0 is the proportion of codons with 0< ω <1 in class 0; p1 is the proportion of codons with ω1 = 1 in class 1; p2a is the proportion of codons with foreground ω2>1 but with background ω0 in class 2a; p2b is the proportion of codons with foreground ω2>1 but with background ω1 = 1 in class 2b.

d Bayes empirical Bayes (BEB) is used to calculate the posterior probabilities to identify the sites (amino acid residues) under positive selection (higher than 95%). The sites are indexed by the amino acids of the site in the human sequence (ALAS1, PBGS, UROD).

In PBGS from arthropods, seven sites are positively selected (model A/A1, *p*<9.8e-7) (BEB >0.964) (Table 3). The amino acid found in arthropod PBGS at position 11 is I/M, whereas F/Y is present at this position in all vertebrate and cnidarian PBGS proteins. We mapped this amino acid in the crystal structure and found that it is positioned at the PBGS dimer interface and is very close to the Mg^2+^ binding site, which has been hypothesized to trigger the conversion of PBGS between its open and closed forms [35]. The amino acid A is present at position 195 in *Drosophila* PBGS, whereas the amino acid at this position is S in all other animal PBGS proteins. The crystal structure indicates that this amino acid forms the active site pocket [35] (Figure S1B).

In UROD from teleosts, seven sites were observed to be positively selected (model A/A1, *p*<4.2e-7) (BEB >0.981) (Table 3). The amino acid S is present at position 290 in teleost UROD, whereas D/E/K is found at this position in other vertebrate, cnidarian and arthropod UROD proteins. The amino acid at position 316 in the teleost UROD is H/R/S, whereas the amino acid at this position is D/E/K in other vertebrate, chordate, arthropod and cnidarian UROD proteins. We mapped both amino acid residues in the crystal structure and found that they are positioned at the UROD dimer interface [36] (Figure S1C).

In summary, we discovered several amino acid residues under positive selection. Some of them are located at the active sites and dimer interfaces of biologically functional enzymes of the heme biosynthesis pathway.

### Detection of Evolutionarily Conserved DNase-hypersensitive Sites in Intron Sequences

**Figure 3:**
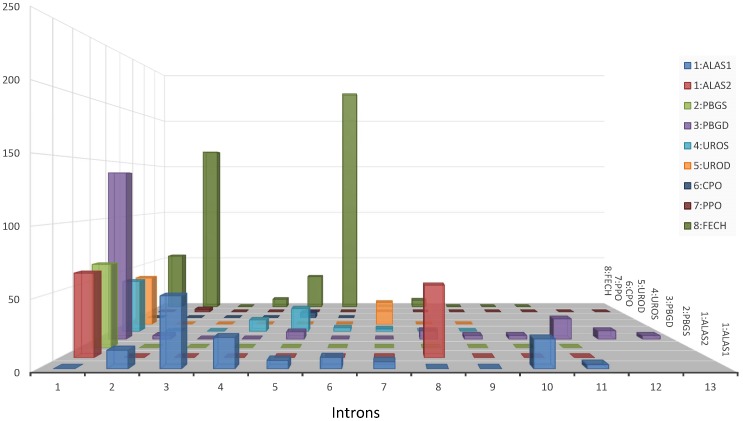
Three-dimensional view of the evolutionarily conserved DNase-hypersensitive sites in intron sequences. For each gene in the biosynthesis pathway (ALAS1 and ALAS2 are treated separately because they are different genes located on different chromosomes), the length of the intersection of the DNA sequence that is evolutionarily conserved across vertebrates and DNase-hypersensitive sites is indicated on the z-axis. The intron ID is provided on the x-axis. Genes from ALAS1 to FECH are shown on the y-axis and are coded from one to eight according to the linear order of the pathway. (Figure 1).

It was previously reported that intron 1 sequences contain *cis*-elements that are necessary for the transcriptional activation of human *PBGS* [37], *PBGD* [38]–[40] and *UROS* [41], [42]. Therefore, we investigated the selection acting on intron sequences by searching for DNA sequences that are both evolutionarily conserved across 46 vertebrates and located in DNase-hypersensitive site clusters (ENCyclopedia of DNA Elements, ENCODE, for human). We found considerable stretches of DNA longer than 40 nucleotides in intron 1 of *ALAS2*, *PBGS*, *PBGD*, *UROS*, *UROD* and *FECH* that could serve as evolutionarily conserved DNase-hypersensitive sites in host genes (Figure 3, Table S2). However, evolutionarily conserved DNase-hypersensitive sites were also found in other intron regions, including in intron 3 of *ALAS1*, intron 8 of *ALAS2*, and introns 2 and 6 of *FECH*. We did not find considerable lengths of evolutionarily conserved DNase-hypersensitive sites in the intron regions of *CPO* and *PPO*. In summary, we revealed evolutionarily conserved DNase-hypersensitive sites in intron regions in six of the eight genes of the heme biosynthesis pathway, implying that conserved regulatory mechanisms acting on intron sequences might be involved in the transactivation of gene expression in the vertebrate heme biosynthesis pathway.

### Distribution of IREs in Exon and Intron Sequences

IREs have been reported to exist in the 5′ and 3′UTR sequences of mRNAs, through which they control the translational efficiency and stability of transcripts, respectively. The 5′UTR of human ALAS2 has been shown to contain an IRE. We conducted a survey of the 5′UTR sequences of genes of the heme biosynthesis pathway (Tables 4 and 5 and Table S3). Notably, the 5′UTRs of nine of the thirteen collected vertebrate ALAS2 sequences contained IREs of high quality (see Methods). Intriguingly, we also detected IREs in the 5′UTRs of teleost ALAS1 sequences and the ALAS sequences of one chordate (tunicate) and the purple sea urchin (echinoderm) that were of high quality as well as in a sea anemone (cnidarian) and honey bee (arthropod) that were of medium quality. These findings constitute the first demonstration that IREs can be identified in the 5′UTRs of ALAS1 sequences from vertebrates and ALAS sequences from arthropods, echinoderms and cnidarians.

**Table 4:** IRE in 5′UTR.

Gene	Mammal	Bird,Reptile,Amphibian	Teleost	Chordate	Echinoderm	Arthropod	Cnidaria
ALAS	NF	NF	NF	1(2)	1(1)	1(2)	1(1)
ALAS1	(5)	(5)	3(4)	NF	NF	NF	NF
ALAS2	5(5)	1(1)	3(3)	NF	NF	NF	NF
PBGS	(6)	(3)	(4)	(1)	NF	(0)	(1)
PBGD	(5)	(3)	(3)	(1)	1(1)	2(2)	(1)
UROS	(5)	(4)	(3)	(0)	(0)	(1)	(1)
UROD	(5)	(1)	(3)	(1)	(1)	(1)	(2)
CPO	(4)	(3)	(4)	(1)	(1)	(1)	(0)
PPO	(5)	(1)	(4)	(0)	(0)	(1)	(2)
FECH	(5)	(0)	(3)	(0)	(0)	(1)	(1)

Number of species containing potential IRE in 5′UTR region of genes involved in heme-biosynthesis pathway.

Number inside the parenthesis is the total number of 5′UTR sequences under investigation.

NF: Not found.

**Table 5:** IRE in intron.

Gene	Mammal	Bird,Reptile,Amphibian	Teleost	Chordate	Echinoderm	Arthropod	Cnidaria
ALAS	NF	NF	NF	2(2)	1(1)	(4)	(2)
ALAS1	1(7)	1(5)	(6)	NF	NF	NF	NF
ALAS2	(5)	1(2)	(5)	NF	NF	NF	NF
PBGS	4(6)	2(4)	1(5)	(1)	NF	(1)	1(3)
PBGD	3(7)	(5)	(6)	(1)	(1)	(4)	1(2)
UROS	5(7)	3(5)	(4)	(1)	(1)	(2)	(2)
UROD	(7)	(5)	(5)	(2)	(1)	(4)	(3)
CPO	(7)	(5)	(5)	(1)	(1)	(1)	(1)
PPO	(7)	1(2)	(5)	(2)	(1)	(1)	1(3)
FECH	5(7)	(4)	(4)	(1)	(1)	(5)	(1)

Number of species containing potential IRE in intron region of genes involved in heme biosynthesis pathway.

Number inside the parenthesis is the total number of intron sequences under investigation.

NF: Not found.

IREs were also found in the 5′UTRs of the PBGD sequences of *Drosophila melanogaster* (fruit fly) and *Apis mellifera* (honey bee) (high quality) as well as that of *Strongylocentrotus purpuratus* (sea urchin) (medium quality) (Tables 4 and 5 and Table S3). This observation raised the possibility that the translation of genes other than ALAS in the heme biosynthesis pathway could also respond to iron availability.

**Figure 4:**
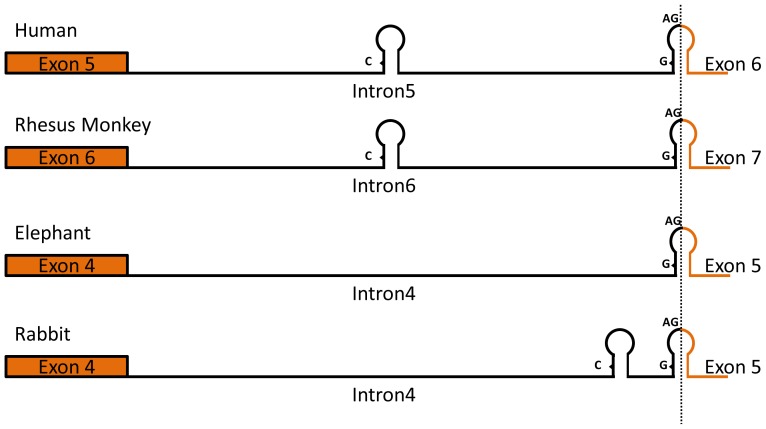
Potential iron-responsive elements (IREs) in the introns and intron-exon boundaries of UROS genes. IREs depicted as stem-loop structures are shown in the corresponding intron regions. UROS exon and intron IDs from four species are indicated. The conserved splicing acceptor site AG and the unpaired nucleotide of the IRE structure are also shown.

**Figure 5:**
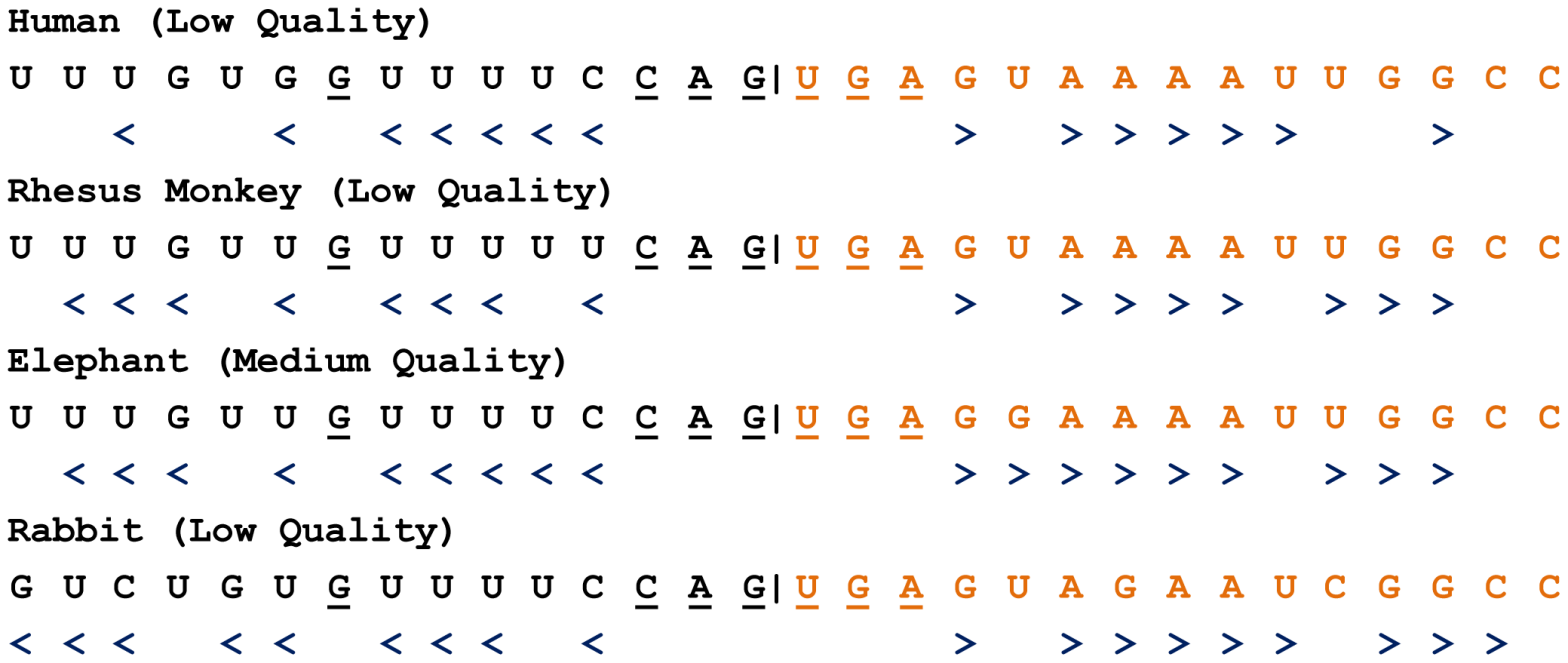
Sequence alignment of the IREs at the intron-exon boundaries of UROS from four species. “>” and “<” represent the base pairing of the RNA secondary structure. The potential IRE consensus loop sequence, CAGUGN, and the unpaired nucleotide G are also shown with respect to the location of the IRE hairpin. The intron-exon boundary is indicated as |.

We also found IREs in intron regions. In particular, several potential IREs (of medium quality) exist at the intron-exon boundaries of PBGS from *Mus musculus* (mouse), PBGD from *Loxodonta africana* (elephant) and *Oryctolagus cuniculus* (rabbit) and UROS from *Loxodonta africana* (elephant) and *Mus musculus* (mouse) (Tables 4 and 5 and Table S3). This class of IREs forms stem and loop regions that overlap with protein-coding and intron sequences (Figure 4 and Figure 5). Based on these observations, we conducted a survey to identify potential IREs in the intron-exon boundary sequences of genes in the human and zebrafish genomes. We found 21 and 12 potential IREs of high quality at the intron-exon boundaries of genes in the human and zebrafish genomes, respectively (Table S4).

In summary, we detected potential IREs not only in the 5′UTR of ALAS2 but also in the 5′UTRs of ALAS and ALAS1. More intriguingly, several potentially conserved IREs might exist at the intron-exon boundaries of genes involved in the heme biosynthesis pathway.

### Distribution of HRMs in Protein Sequences

The HRM (denoted HRM_t, see Methods) has been shown to bind heme to inhibit the import of ALAS1 into mitochondria. Here, we collected recently published HRM sequences and compiled them into a new HRM consensus sequence (denoted HRM_r, see Methods). Some of the HRM_r sequences are predicted to sense the redox state of the cell and may be critical for triggering the degradation of proteins containing the HRM sequence.

Only ALAS1, ALAS2 and PBGD protein sequences contain HRM_t. We found HRM_t sequences in the ALAS sequences of chordates, echinoderms, and cnidarians. The ALAS1 genes of all vertebrate species show HRM_t sequences at the N-terminus. In ALAS2, HRM_t sequences were found in several types of mammals and in chickens and Xenopus (Tables 6 and 7 and Table S5).

**Table 6:** HRM_t in protein sequence.

Gene	Mammal	Bird,Reptile,Amphibian	Teleost	Chordate	Echinoderm	Arthropod	Cnidaria
ALAS	NF	NF	NF	3(3)	2(2)	1(6)	1(2)
ALAS1	7(7)	6(6)	6(6)	NF	NF	NF	NF
ALAS2	4(5)	3(4)	(5)	NF	NF	NF	NF
PBGS	(6)	(5)	(5)	(1)	NF	(6)	(3)
PBGD	(7)	(6)	(5)	(1)	(1)	3(6)	(2)
UROS	(7)	(6)	(4)	(2)	(1)	(6)	(3)
UROD	(7)	(6)	(5)	(3)	(1)	(7)	(3)
CPO	(7)	(5)	(5)	(2)	(1)	(6)	(1)
PPO	(7)	(5)	(5)	(2)	(1)	(6)	(3)
FECH	(7)	(5)	(4)	(3)	(1)	(7)	(2)

Number of protein species containing potential HRM_t of genes involved in heme-biosyntheis pathway.

Number inside the parenthesis is the total number of protein sequences under investigation.

NF: Not found.

**Table 7:** HRM_r in protein sequence.

Gene	Mammal	Bird,Reptile,Amphibian	Teleost	Chordate	Echinoderm	Arthropod	Cnidaria
ALAS	NF	NF	NF	(3)	(2)	(6)	(2)
ALAS1	(7)	2(6)	(6)	NF	NF	NF	NF
ALAS2	5(5)	(4)	1(5)	NF	NF	NF	NF
PBGS	6(6)	5(5)	5(5)	(1)	NF	2(6)	1(3)
PBGD	(7)	(6)	(5)	(1)	(1)	3(6)	(2)
UROS	(7)	(6)	(4)	(2)	(1)	(6)	(3)
UROD	(7)	(6)	(5)	(3)	(1)	(7)	(3)
CPO	(7)	(5)	(5)	(2)	(1)	(6)	(1)
PPO	(7)	(5)	(5)	(2)	(1)	(6)	(3)
FECH	(7)	(5)	(4)	(3)	(1)	(7)	(2)

Number of protein species containing potential HRM_r of genes in heme biosynthesis pathway.

Number inside the parenthesis is the total number of protein sequences under investigation.

NF: Not found.

**Figure 6:**
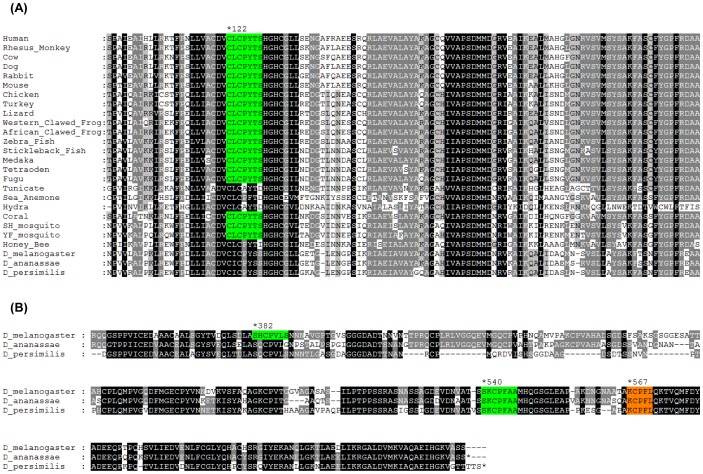
Heme-regulatory motifs (HRMs) in PBGS and PBGD. Multiple sequence alignments of PBGS (A) and PBGD (B) are shown, with HRM_t and HRM_r colored orange and green, respectively. Amino acid numbers for HRM_t and HRM_r are also shown according to the first protein sequence in the alignment.

Notably, when we used HRM_r as the search sequence for HRMs, new HRM sequences were identified in PBGS (vertebrates, arthropods, and cnidarians) and PBGD (*Drosophila*). This class of HRM is not restricted to the N-terminus of the protein sequence and is conserved with respect to its position in the amino acid sequence (Figure 6).

## Discussion

Elucidation of the evolutionary history of biological pathways sheds light on the principles underlying the evolutionary forces acting on organisms in the environment. The history of pathway evolution may vary among different pathways and ancestral organisms, but understanding the underlying principles helps to reveal the modifications that have taken place in the physiological processes of organisms during their evolutionary history.

Methods allowing the detection of selection pressure in the evolutionary histories of pathways and networks have made it possible to investigate the existence of fundamental principles driving selection in nature. By estimating the ratio of the nonsynonymous to the synonymous substitution rate for individual protein-encoding genes (ω), the types of selection pressure acting on a gene can be identified. In addition to the properties of proteins themselves, the regulatory mechanisms acting on genes are important in metabolic pathways and are also exposed to selection during evolutionary processes. A novel regulatory mechanism affecting the genes in a metabolic pathway can give rise to a new gene function that may become fixed in a lineage, through which we can determine the evolutionary history of a particular biosynthesis pathway throughout different lineages of organisms. To clarify the evolutionary history of the heme biosynthetic pathway of animals, we analyzed the three previously reported regulatory mechanisms related to genes in the heme biosynthesis pathway, which involve DNase I-hypersensitive sites, IREs, and HRMs. Our *in silico* prediction results showed that multiple regulatory mechanisms may exist for the genes in the heme biosynthesis pathway.

### Stronger Selection at the Middle and Penultimate Positions of the Pathway could Result from Self-catalysis

We conducted a phylogenetic analysis of the eight genes of the heme biosynthesis pathway found in the animal kingdom. The ω values at positions four (UROS) and seven (PPO) were shown to be significantly higher than at the other positions in the pathway. Notably, some self-catalysis and by-products of the heme biosynthesis pathway have been reported. Hydroxymethylbilane, the substrate of UROS, can be non-enzymatically cyclized to form uroporphyrinogen I, a useless by-product that leads to uroporphyrin I or coproporphyrin I [43]. The product of UROS can also be auto-oxidized to uroporphyrin III. Protoporphyrinogen, the substrate of PPO, has been shown to be auto-oxidized to protoporphyrin in air, without the need for PPO [43], [44]. The degree of such “leaking” among the biochemical reactions involved in the heme biosynthesis pathway is unknown, and whether this phenomenon is common in animals remains to be determined. We speculate that “leaking” in a biochemical reaction would impact selection pressure and, most likely, lead to decreased evolutionary constraint.

### Biological Function of Evolutionarily Conserved DNase-hypersensitive Sites in Intron Sequences

Based on the chromatin immunoprecipitation with high-throughput sequencing (ChIP-seq) technique, the transcription factor-binding module GATA1-KLF1 has been hypothesized to allow erythrocyte-specific activation of gene expression through binding to the intron 1 sequences of *ALAS2*, *PBGS*, *PBGD* and *UROS* [21], [22]. In this study, we identified intron 1 sequences as a type of DNase-hypersensitive site that is conserved in vertebrates among several genes, including *ALAS2*, *PBGS*, *PBGD*, *UROS* and *UROD*. This finding suggests that in response to the high demand for heme in the erythrocytes of the common ancestor of vertebrates, several transcription factors were recruited to bind to the first intron region to trans-activate the expression of the first five genes of the heme biosynthesis pathway. Notably, an intronic enhancer has been identified in intron 8 of *ALAS2* in mouse erythroleukemia cells [45]–[47], which is consistent with our analysis (Figure 2). We also identified introns 2 and 6 of *FECH* as potential intronic enhancers, although two promoter regions of mouse *FECH* were shown to function in basic and inducible expression [48].

The use of the first intron as an alternative promoter to induce erythrocyte-specific expression has been documented for several hematopoietic genes, including *Abcg2* [49], [50], *Ank1* [51] and *Slc11a2* [21]. We also determined whether evolutionarily conserved intron regions of these genes could be detected. Without exception, the first intron regions of all three genes contain long stretches of DNA that are both conserved across vertebrates and accessible to DNase attack, suggesting that transcription factor binding occurs (Table S6). The high frequency of alternative first exons found in erythroid genes has been shown to be crucial for the regulation of gene function [52], and we propose here that the evolutionarily conserved region in the first intron can serve as either an alternative promoter or enhancer to enable alteration of the first exon during gene transcription [53].

### Elucidation of Novel Evolutionarily Conserved IREs at Intron-exon Boundaries

An IRE was previously identified in the 5′UTR of ALAS2 [54]. However, the detection IREs in the 5′UTRs of PBGD sequences is reported for the first time here. The scattered appearance of IREs in enzymes other than ALAS suggests that animal genes acquired IREs at a later point through the process of convergent evolution [55]. Moreover, whereas the noted IRE found in an echinoderm ALAS (*Ciona intestinalis*) has been reported previously [55], our identification of a potential IRE in the ALAS of a cnidarian (sea anemone) and an arthropod (honey bee) suggested that a more thorough search for the existence of IREs in animals is necessary.

Both the 5′UTR and 3′UTR are commonly regarded as sites where IREs are located. However, we found that intron sequences can also contain IREs. In particular, we identified an IRE at the intron 4-exon 5 boundary of UROS in *Loxodonta africana* (elephant). We also aligned the IRE in the corresponding region and found that this intron-exon IRE is conserved in humans, rhesus monkeys and rabbits, although these IREs are of low quality. If IREs found at intron-exon boundaries are functional, it is possible that the pre-mRNA splicing junction could be bound by IRP, thereby influencing splicing efficiency or choice.

In the iron-depleted state, IRP1 and IRP2 are stable and bind to IREs, either to inhibit protein translation (5′UTR) or to prevent mRNA degradation (3′UTR). In the iron-replete state, the degradation of IRP2 allows IREs to bind iron and eIF4E [56] to initiate protein translation. Intriguingly, eIF4E has been demonstrated to function as a co-factor in the *Sxl*-dependent female-specific alternative splicing of *msl*-2 as well as *Sxl* premRNAs in *Drosophila*, which is required for sex determination due to the silencing of the X chromosome [57]. This observation raised the possibility that eIF4E could be involved in splicing events in mammals. To determine how many IREs exist at intron-exon boundaries, we conducted a genome-wide survey to detect IREs of high quality with respect to the nucleotides present at the junctions of CDS-exons and introns in the human and zebrafish genomes. We found 21 and 12 high-quality IREs at human and zebrafish CDS-exon/intron junctions, respectively (Table S4). One of human genes, ZNF446, has been found in the Friendly Alternative Splicing and Transcripts Database (FAST DB) [58] that contains an alternatively spliced transcript (AY279351), which presumably depends on the corresponding junction containing an IRE. This possible phenomenon adds one more layer of complexity to the regulation of genes in the heme biosynthesis pathway in particular and to the regulation of erythropoiesis in general. Furthermore, this finding also calls for an investigation into the mechanism by which iron and IRP regulate the alternative splicing of protein-coding genes and the associated biological effects.

### Elucidation of Novel Evolutionarily Conserved HRMs

By defining a new HRM (HRM_r), we were able to identify HRMs in most of the studied PBGS genes (76%, 19/25) and all PBGD genes from *Drosophila*. Recently, HRMs have been implicated in regulatory functions other than controlling import into the mitochondria. The HRM in human IRP2 has been shown to be responsible for the ubiquitin-targeted degradation of the IRP2 protein [59]. The degradation of human circadian Factor Period 2 (hPer2) is also mediated by an HRM, suggesting that metabolic signals can modulate the circadian regulation of gene expression [60]. Notably, mouse *ALAS1* and *ALAS2* have been shown to be under circadian control and are regulated by mPER1 and mPER2 [61]. It has been demonstrated that the HRM of ALAS2 is not involved in the heme-mediated control of import into the mitochondria [32]. Therefore, we postulate that the HRMs identified in ALAS2 and PBGS could be involved in protein degradation. This is consistent with the localization of the PBGS enzyme in the cytosol and that, unlike ALAS1 and ALAS2, PBGS does not need to enter the mitochondria to function (Figure 1). The HRM binds heme and senses both the concentration of heme and the oxidation/reduction state of the cell. Our findings indicate that product feedback control of protein stability could be involved in the evolution of the heme biosynthesis pathway.

### Evolutionary Implications of HRMs and IREs in the Teleost Lineage based on Comparison with other Species

We found HRMs in the ALAS sequences of chordates and echinoderms. ALAS1 HRM_t exists in all investigated vertebrate species as well. While we also detected ALAS2 HRM_t in certain monophyletic groups (mammals, birds, and amphibians), we did not find any HRM_t sequences in teleosts. Additionally, we identified IREs in the 5′UTRs of ALAS sequences from chordates and echinoderms. In the 5′UTR of ALAS2, there is an IRE in all of the examined vertebrate species. In the 5′UTR of ALAS1, an IRE is found in teleosts, but not in mammals. Based on our phylogenetic analysis, chordate and echinoderm ALAS and vertebrate ALAS1/2 are derived from the same ancestral ALAS gene. We propose that the HRMs and IREs observed in ALAS1 and ALAS2 existed in the ALAS sequence of a common ancestor of vertebrates, chordates, and echinoderms. The unique absence of HRMs in ALAS2 proteins and the presence of IREs in ALAS1 mRNAs in teleosts are striking. We further propose that during the evolutionary branching and speciation of vertebrates, the loss of the HRM from ALAS2 and the retention of the IRE in ALAS1 in teleosts resulted from the adaptation of the heme biosynthesis pathway to the environment. Notably, we have also shown that the amino acid sequences of ALAS2 and UROD have experienced positive selection in the teleost lineage.

### Conclusion: An Integrated View of the Evolution of Multiple Types of Regulation vs. Pathway Position Reveals Different Depths within the Pathway

We have investigated the evolution of multiple controls modulating the heme biosynthesis pathway. It was illuminating to study the degree by which these combinatorial controls impinge on the pathway from top to bottom, if we regard ALAS, at position one, as the top and FECH, at position eight, as the bottom. In a summary table (Table 8), with the eight genes represented from positions one to eight in rows and the six control mechanisms across the top of the table, it can clearly be observed that there are multiple degrees of regulatory potential with respect to this pathway. Notably, transcriptional control and iron-mediated splicing control infiltrate the middle and bottom of the pathway. Substrate (iron) or product (heme) feedback translational control as well as protein localization and protein stability control primarily infiltrate the top of the pathway. Purifying selection on the protein sequences is widespread but is subject to loose constraint in the middle and near the bottom of the pathway. An intriguing question from the point of view of pathway position is whether the evolvability of pathway regulation depends on the position of the genes in the pathway, thereby leading to differential adaptation. Our research suggests that the investigation of the molecular evolution of a pathway should involve the examination of different control mechanisms related to the activity of the genes that constitute the pathway under investigation. Thus, a new perspective in the field of evolutionary developmental biology could concern the roles played by the *cis*-regulatory regions and protein-coding regions of genes with respect to adaptive mutations [62], [63]. We are aware that our key findings in this study are predictions based on the current understanding of the heme biosynthesis pathway, and these findings will require substantial experimental work to be confirmed or corrected.

**Table 8:** Multiple regulatory potentials in heme biosynthesis pathway.

Gene	Pathway Position	First Intron-mediatedTranscription Control^a^	Splicing Control^b^	Translational Control^c^	Protein Localization^d^	ProteinStability^e^	Selection Pressure^f^
ALAS	1			**	**		
ALAS1	1			*	***	*	
ALAS2	1	**		***	**	**	
PBGS	2	**	*			***	
PBGD	3	***	*	*		*	
UROS	4	*	*				***
UROD	5	*					
CPO	6						
PPO	7						***
FECH	8	**					

a Evolutionarily conserved DNase-hypersensitive sites in intron sequences.

b IRE in intron-exon boundary that could potentially affect splicing.

c IRE in 5′UTR,while binding IRP, could potentially inhibit protein translation.

d HRM_t that could potentially block the import of enzyme to mitochondria while binding heme.

e HRM_r that could potentially affect protein stability.

f Genes with two highest ω value.

In ^a^, *: <50 bps, **:>50 bps, <100 bps, ***:>100 bps. In ^bcde^, *:< = 0.33, **:>0.33, < = 0.67, ***:>0.67 for the proportion of species collected.

## Materials and Methods

### Sequence Collection

For genes encoding the eight enzymes of the heme biosynthesis pathway in animals, we collected the sequences of organisms whose entire genome sequences were available. Sequences were collected from mammals (seven species), amphibians (two species), birds (three species), reptiles (one species), teleosts (six species), echinoderms (two species), arthropods (eight species) and cnidarians (three species). The amino acid sequences, coding nucleotide sequences, exon sequences and intron sequences were downloaded from NCBI, UCSC [64], [65] and Ensembl [66]. Some of the protein sequences of ALAS genes were extracted as suggested previously [19]. The 5′UTR, exon and intron sequences of the coral *Acropora digitifera* came from OIST Marine Genome Unit [67]. The 5′UTR, exon and intron sequences of the sea anemone *Nematostella vectensis* were obtained from the DOE Joint Genome Institute [68]. The 5′UTR, exon and intron sequences of the hydra *Hydra magnipapillata* also came from the DOE Joint Genome Institute [69].

If a gene model lacked a 5′UTR, we searched for a cDNA collection to determine whether there was any possibility of extension in the 5′ direction with respect to the direction of transcription. If such an extension was possible, a 5′UTR was added to the gene model. Subsequently, if this extension added a new exon, which was regarded as the first exon, then a new intron, which was usually the first intron, was hypothesized to exist. The numbers of coding sequences obtained for each gene in the heme biosynthesis pathway were as follows: ALAS (46 genes from 31 species), PBGS (26 genes from 26 species), PBGD (29 genes from 28 species), UROS (28 genes from 28 species), UROD (31 genes from 31 species), CPO (27 genes from 27 species), PPO (26 genes from 26 species) and FECH (30 genes from 30 species) (Table S7). Lists about the number of sequences and the species by taxonomic groups can be found in Table S8 and S9. Sequences from species whose genome had not been decoded were not included in this study.

### Sequence Alignment and Phylogenetic Analysis

The amino acid sequence alignment of the eight enzymes involved in the heme biosynthesis pathway was performed in MEGA5 with the MUSCLE algorithm [70]. After the aligned sequences were adjusted manually to confirm their accuracy, topologies of the phylogenetic trees were generated with PHYLIP [71] and PHYML [72] via maximum likelihood (ML) methods. The gamma rate heterogeneity model and the JTT substitution model from Tree-Puzzle [73] and MEGA5 were used for the enzymes of the heme biosynthesis pathway. Branch support was provided via bootstrap analysis, involving a heuristic search with 1000 replicates. The alignments and trees are shown in Figures S2 and S3, respectively.

### Analysis of Evolutionary Constraints

The aligned nucleotide coding sequences without gaps based on the aligned protein sequences and the unrooted tree were fed into the CODEML program of the PAML program package (version V4.4e) [74], [75] to analyze the evolutionary constraints on the coding sequences for the eight genes of the heme biosynthesis pathway. For the different models applied, ω (the nonsynonymous versus synonymous rate, *d_N_/d_S_*) for codons can be assumed to be less than one (negative selection or purifying selection), equal to one (neutral), or greater than one (positive selection).

First, we obtained ω for each gene by applying the null model M0, assuming a constant ω value for all codons and branches. Second, site models M1a (Nearly Neutral) and M2a (Positive Selection) were both used to allow ω to vary among sites. In the M1a model, the codons are categorized into two types, one of which shows ω values of less than one, while ω is equal to one of the other type. In the M2a model, the codons are categorized into three types, one with ω values less than one, one for which ω is equal to one and one with ω values greater than one. We performed the likelihood ratio test for the M1a and M2a models compared to the null model, M0 (twice the log-likelihood difference (2Δln*L*) of the two models).

We also used the branch model to analyze the evolutionary constraints on the mammal, teleost and arthropod branches. We then compared the likelihood of the null model, M0, with the branch model. If the likelihood of the branch model were to be significantly higher than that of the null model M0, it was hypothesized that the branch under consideration is potentially under positive selection. Subsequently, we used the branch-site model to allow variation among sites in the proteins and across branches to determine whether any amino acid residues were under positive selection [76]. For branch-site model A, the following four classes were demarcated for each amino acid: class 0 with 0< ω_0_<1 in all branches; class 1 with ω_1_ = 1 in all branches; class 2a with foreground ω_2_≥1 but background 0< ω_0_<1; and class 2b with a foreground ω_2_≥1 but background ω_1_ = 1. Null model A1 was the same as A but with the foreground ω_2_ constrained to one. A likelihood ratio test is used between models A and A1. If the likelihood of model A were to be significantly higher than the likelihood of model A1 (null model) (*p*<0.05), it would indicate that there were amino acid residues under positive selection. Bayes empirical Bayes (BEB) was used to calculate the posterior probability of identifying sites under positive selection (>0.95) [77]. When we describe the position of the amino acids under positive selection, we use the human sequences (for ALAS1, PBGS and UROD) as a reference to index the sequences. We mapped the positively selected amino acids onto the crystal structures of ALAS [34], PBGS [35] and UROD [36].

We also compared the ω values among the eight genes using a previously described method [2], [8]. When the difference of the ω values between two genes, such as gene A, with ω_A_ and a likelihood of L_A0_ in the M0 null model, and gene B, with ω_B_ and a likelihood of L_B0_ in the M0 null model, was significant, we were able to find a ω_n_ between the two genes that was significantly different from those of the two genes being compared. To perform this test, we set ω_n_ as the average ω value of the two genes being compared (ω_n_ = (ω_A_+ω_B_)/2). We then acquired the likelihood values (L_A_ and L_B_) after we constrained the ω of genes A and B as ω_n_. Subsequently, the statistical significance (df = 1, *p*<0.05) of the difference in ω was determined from the difference in the likelihood between the null model M0 and the constrained model, as follows: 2*(L_A_ – L_A0_) and 2*(L_B_ – L_B0_).

### Detection of Evolutionarily Conserved DNase-hypersensitive Sites in Intron Sequences

We used the UCSC table browser [78], [79] and Galaxy [80] to extract the intron sequences at the intersections of regions that are conserved in vertebrates (hg19, phastConsElements46way) and DNase-hypersensitive sites (hg19, wgEncodeRegDnaseClustered) [81].

### Detection of IREs

We used the SIRE web service to identify potential IRE sites in the gene sequences [82]. SIRE takes into consideration the non-canonical sequence as indicated by SELEX. By allowing 18 motifs to be confirmed as binding to IRP1 or IRP2, SIRE accepted the input sequence and reported the motif type, the free energy of the secondary structure and the level of stringency as High, Medium, or Low. A batch version of the same algorithm was also developed for the genome-wide detection of IREs at the intron-exon boundaries of human and zebrafish genes.

### Detection of HRMs

The HRM sequence, N/K/R-C-P-K or a hydrophobic residue-L/M, has been commonly used to detect HRMs [19], [31] (denoted HRM_t). By identifying new occurrences of HRMs that have been shown to function biologically (human ALAS1; human ALAS2 [32]; human, mouse, rat, spalax, and zebrafish PER2 [60]; human IRP2 [59]; human STC2 [83]; and human, mouse and rabbit eIF2alpha [84]), we also identified a new HRM motif, denoted HRM_r, A/C/F/G/I/R/S/Q-A/C/K/H/L/N/R/S/T-C-P-A/E/F/I/K/S/V/Y-A/D/H/I/L/M/T/V-A/L/M/P/R/S. We used seven sites in HRM_r rather than five sites in HRM_t to increase the specificity of HRM_r. We note that HRM_r is similar to, but not the same as HRM_t.

## Supporting Information

Figure S1 **The collection of figures describing the protein structures of enzymes involved in the heme biosynthesis pathway and the positions of the positively selected residues.** Homodimer structures are shown with the monomers colored in white and yellow. Positively selected amino acid residues are colored in red. Substrate analogs or prosthetic groups are colored in green. (A) ALAS2 of teleost; (B) PBGS of arthropod; (C) UROD of teleost.
(PDF)

Figure S2 **The aligned protein sequences of eight genes of heme biosynthesis pathway in animals.**
(PDF)

Figure S3 **The collection of figures for the maximum likelihood phylogeny of protein sequences for eight genes of heme biosynthesis pathway in animals.** Bootstrap values >70% are indicated. The bootstrap values are displayed only for the branches of the main lineages. (A) ALAS, (B) PBGS, (C) PBGD, (D) UROS, (E) UROD, (F) CPO, (G) PPO, (H) FECH.
(PDF)

Table S1 **Model test (M1a) for selection of genes in heme biosynthesis pathway.**
(PDF)

Table S2 **Length of evolutionarily conserved DNase-hypersensitive sites in intron sequences.**
(PDF)

Table S3 **Potential IRE in eight genes of heme biosynthesis pathway.**
(PDF)

Table S4 **Genomewide detection of potential IRE in exon-intron boundary in human and zebrafish genes.**
(PDF)

Table S5 **Potential HRM in eight genes of heme biosynthesis pathway (HRM_t and HRM_r).**
(PDF)

Table S6 **Length of evolutionarily conserved DNase-hypersensitive sites in intron sequences (bps) for** ***Abcg2*** **,** ***Ank1*** **and** ***Slc11a2*** **.**
(PDF)

Table S7 **IDs of genes of heme biosynthesis pathway in animals.**
(PDF)

Table S8 **Number of sequences by taxonomic groups.**
(PDF)

Table S9 **Species by taxonomic groups.**
(PDF)
