# Supplementary figures and images for: Molecular Evolution of Multiple-Level Control of Heme Biosynthesis Pathway in Animal Kingdom
Source: PLoS One. 2014 Jan 28;9(1):e86718. doi: 10.1371/journal.pone.0086718 (PMC3904948; doi:10.1371/journal.pone.0086718)

(A)

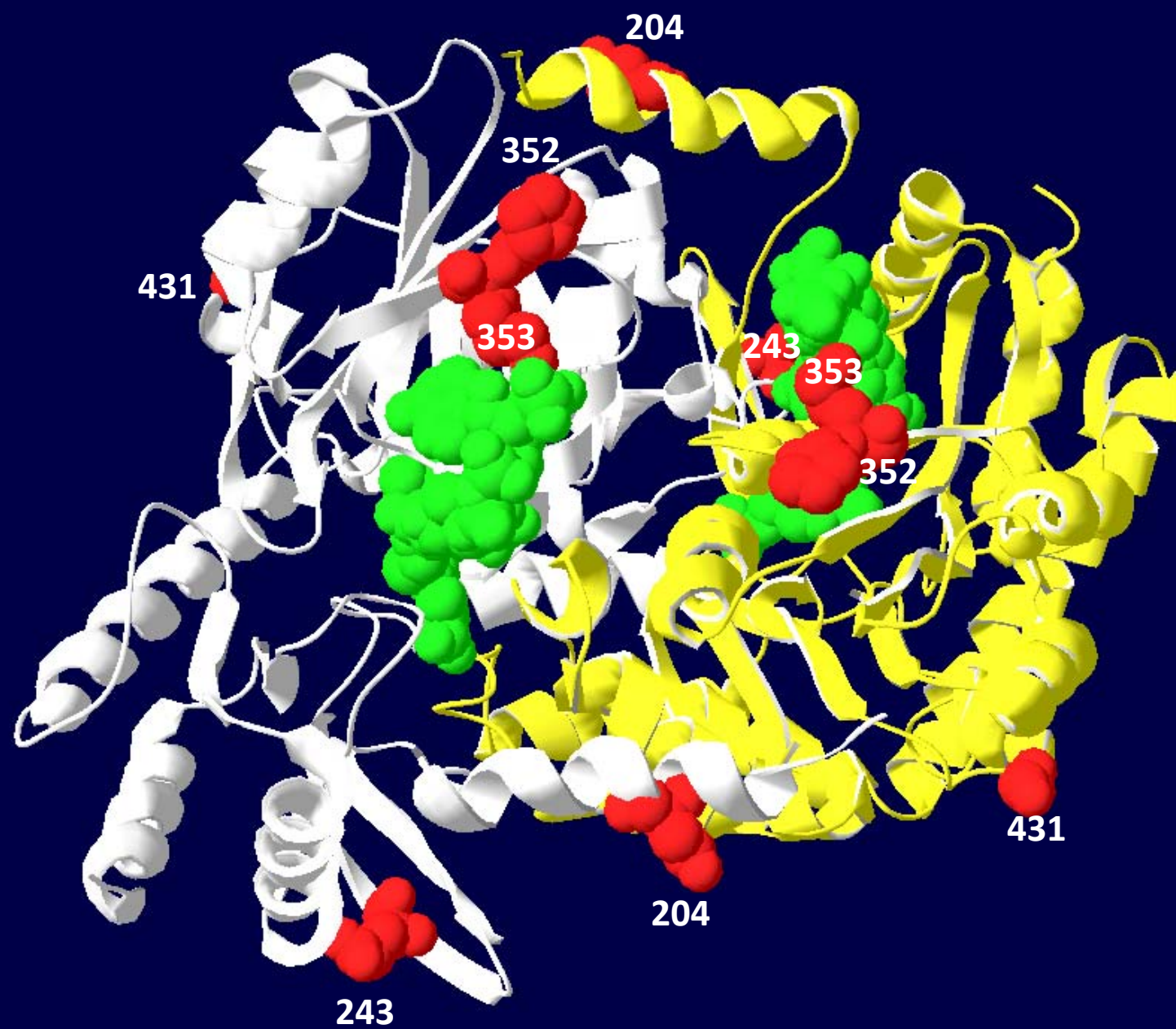

(B)

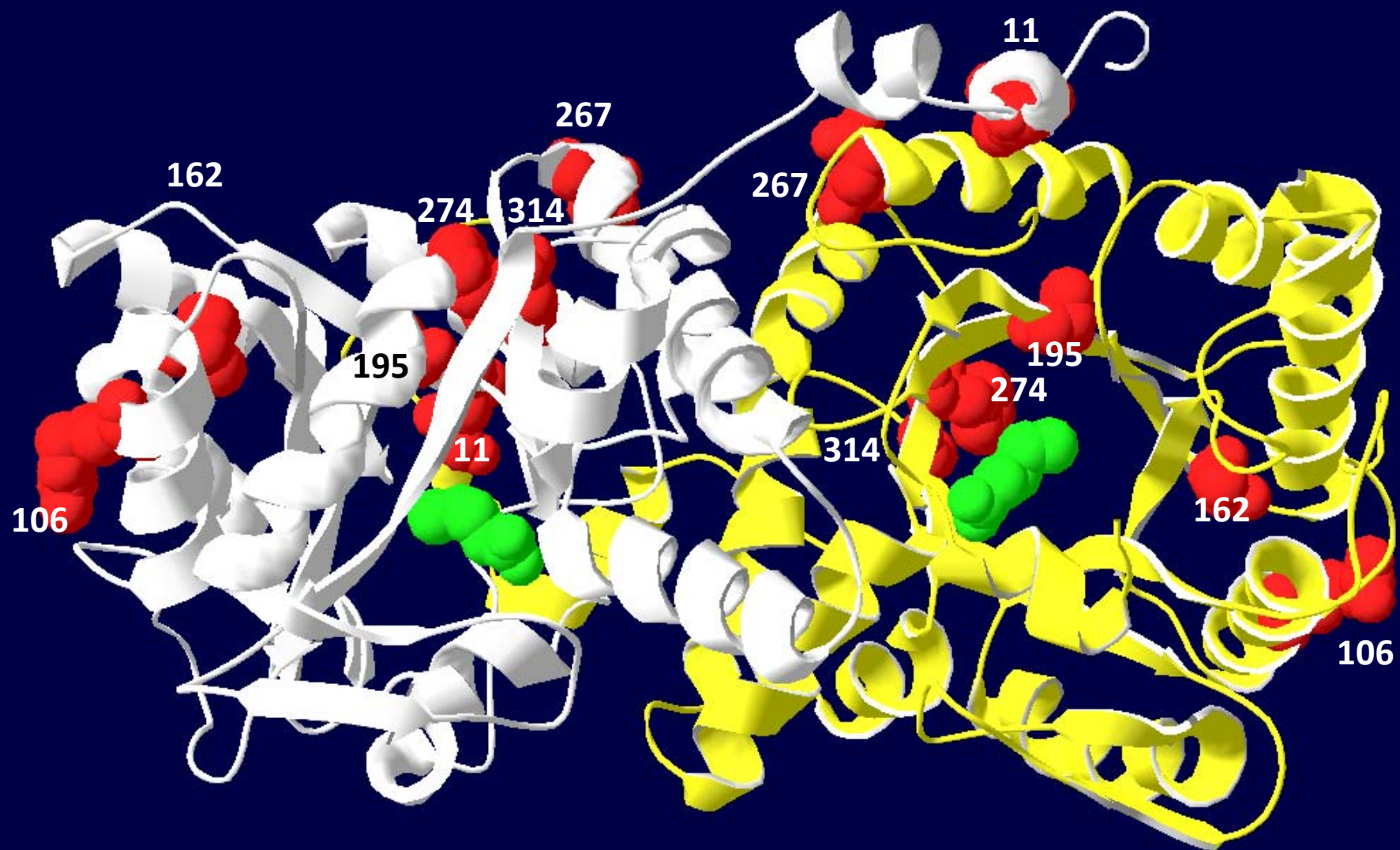

(c)

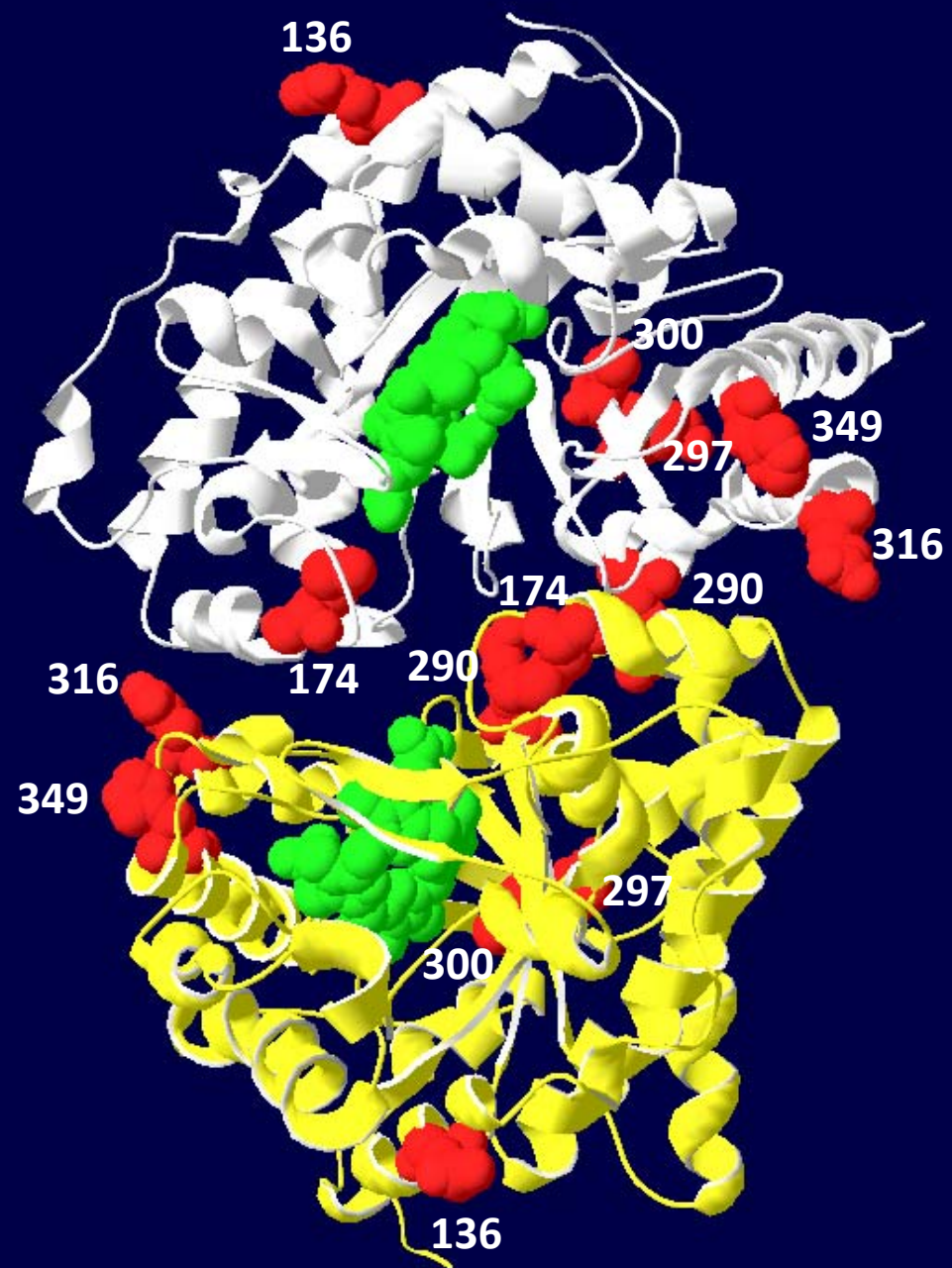

Supplement: Figure S1 — The collection of figures describing the protein structures of enzymes involved in the heme biosynthesis pathway and the positions of the positively selected residues. Homodimer structures are shown with the monomers colored in white and yellow. Positively selected amino acid residues are colored in red. Substrate analogs or prosthetic groups are colored in green. (A) ALAS2 of teleost; (B) PBGS of arthropod; (C) UROD of teleost. (PDF) [file pone.0086718.s001.pdf]

(A)

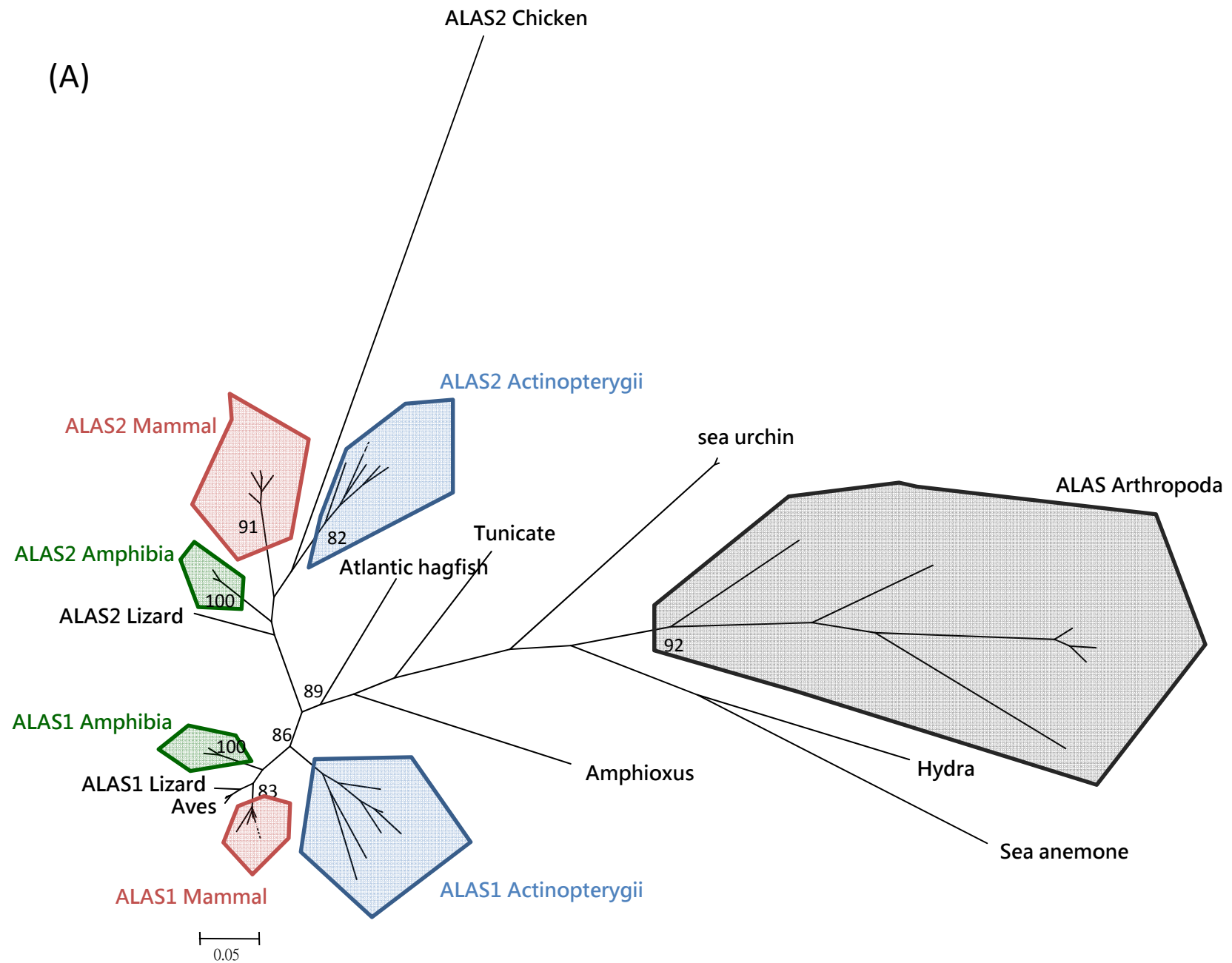

(B)

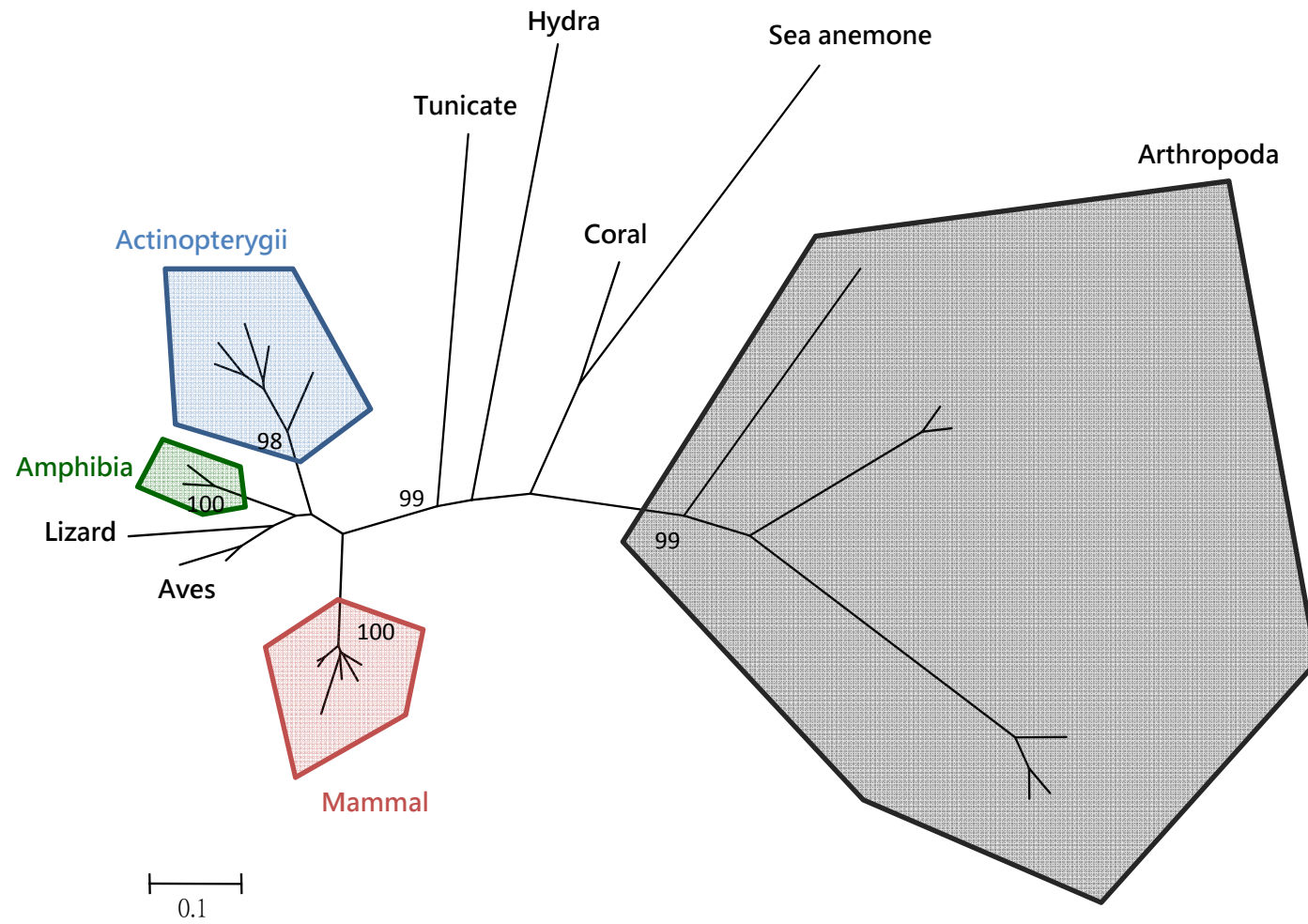

(C)

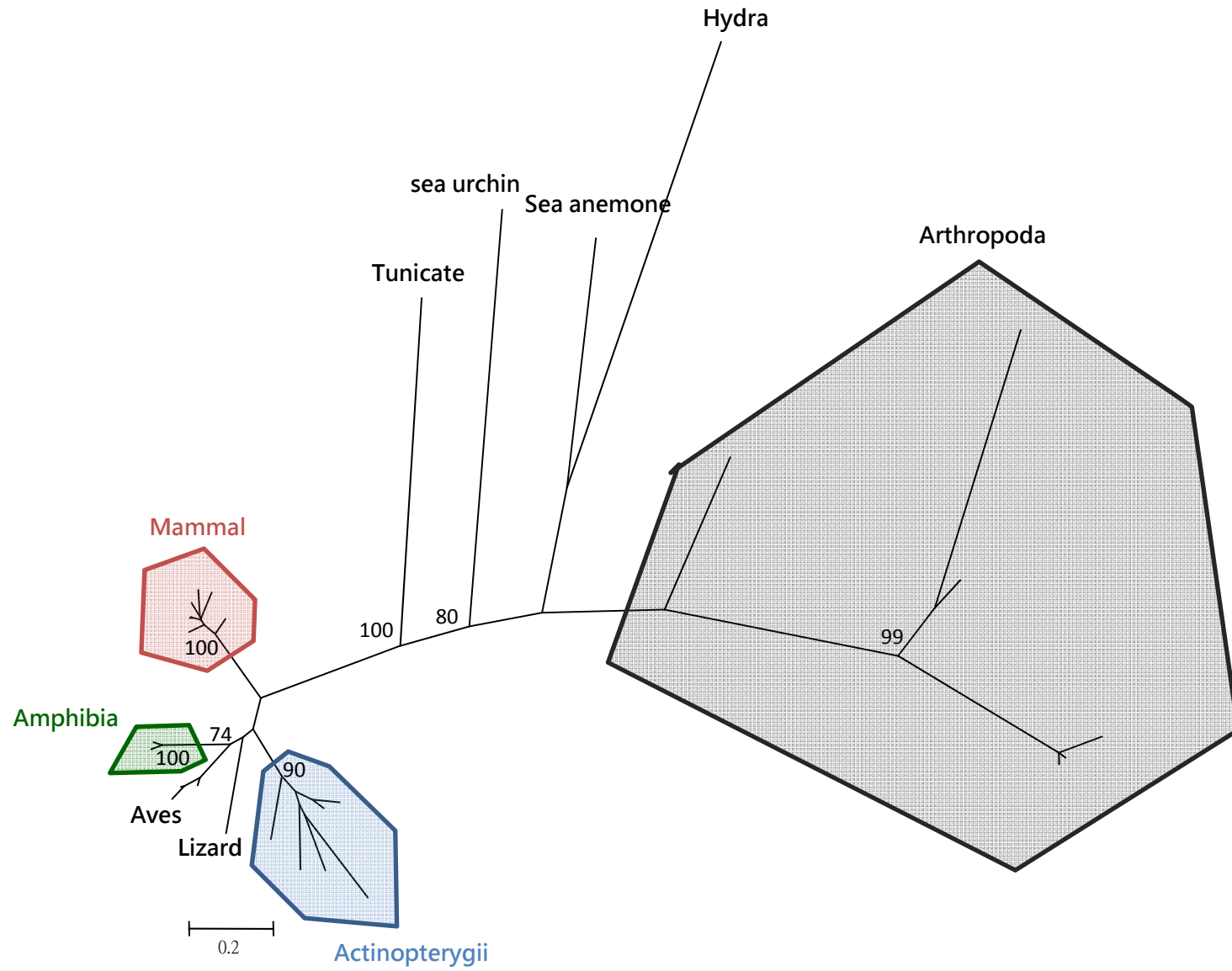

(D)

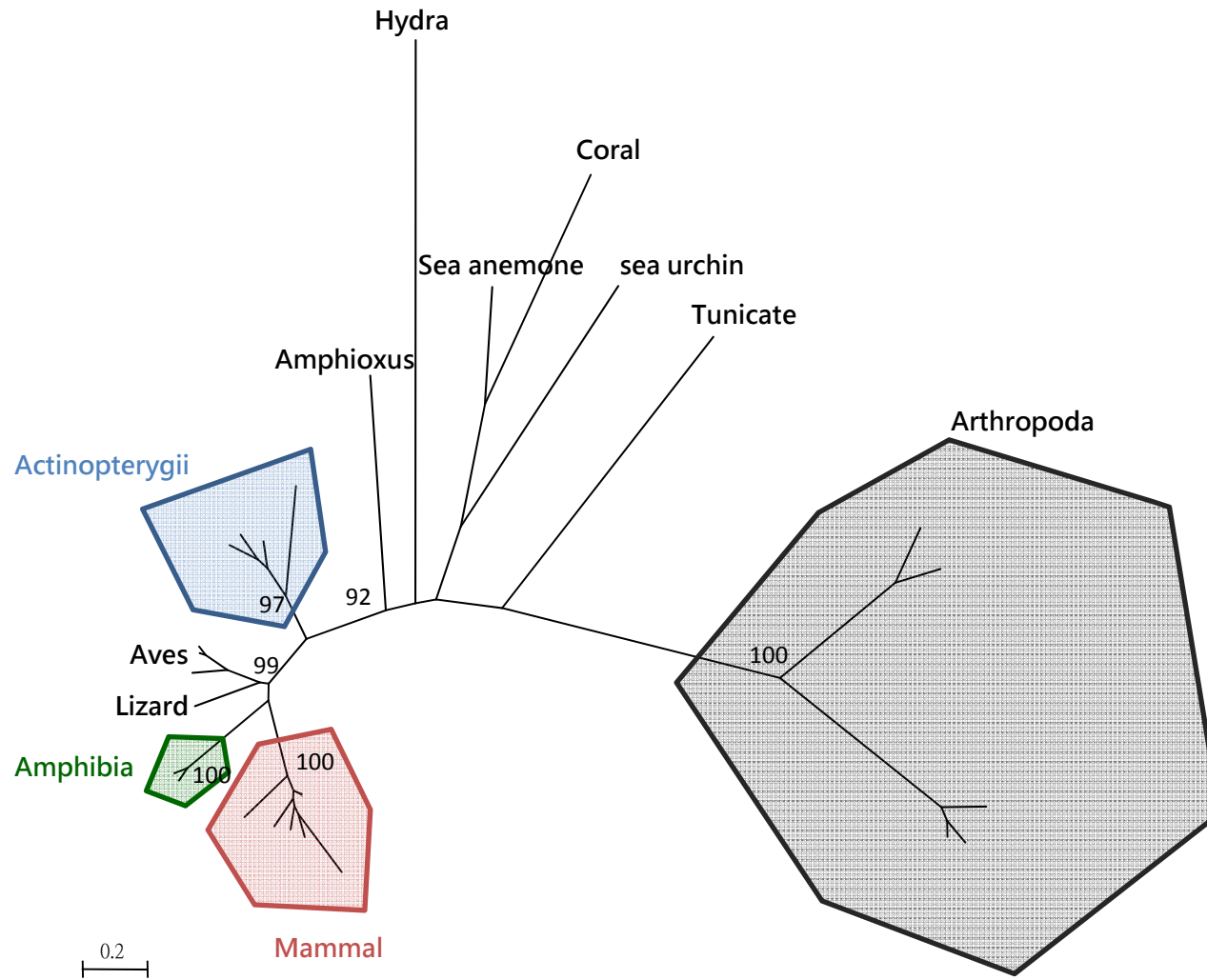

(E)

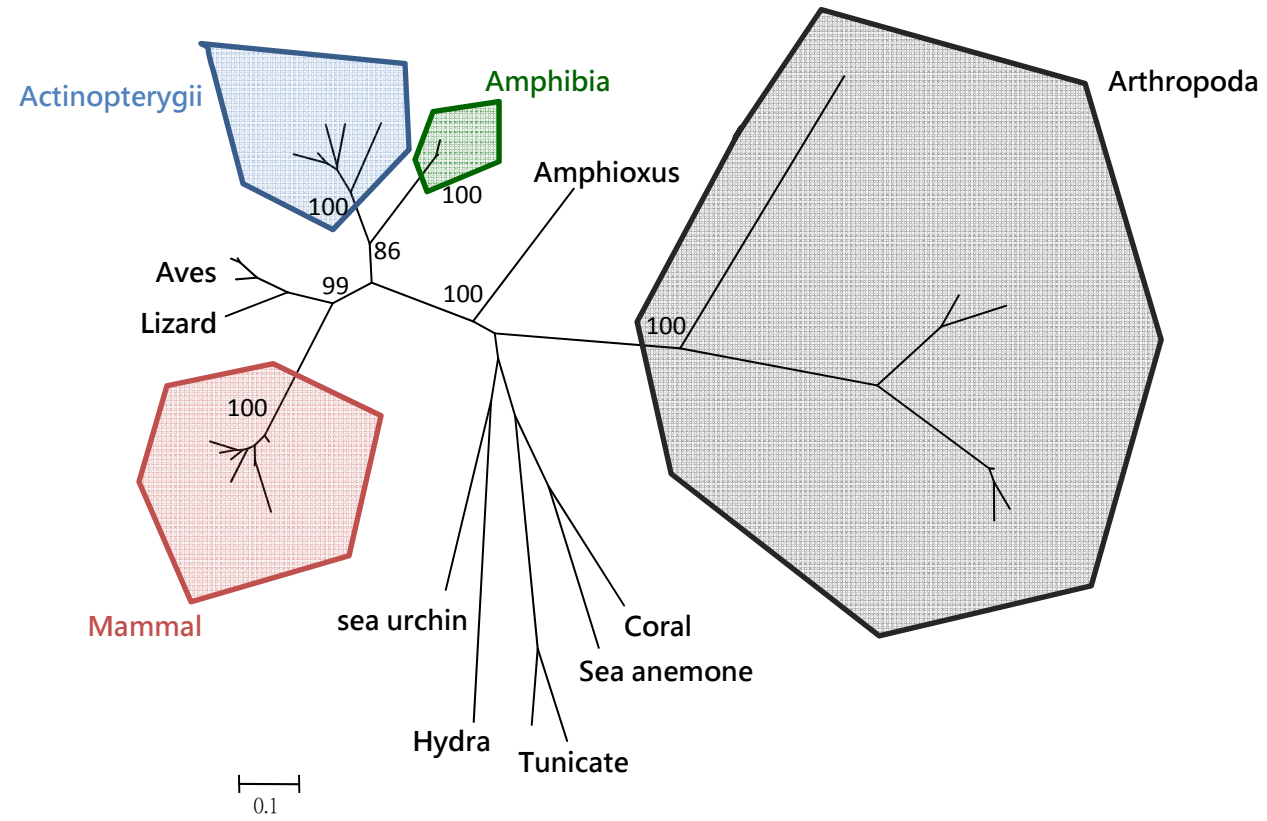

(F)

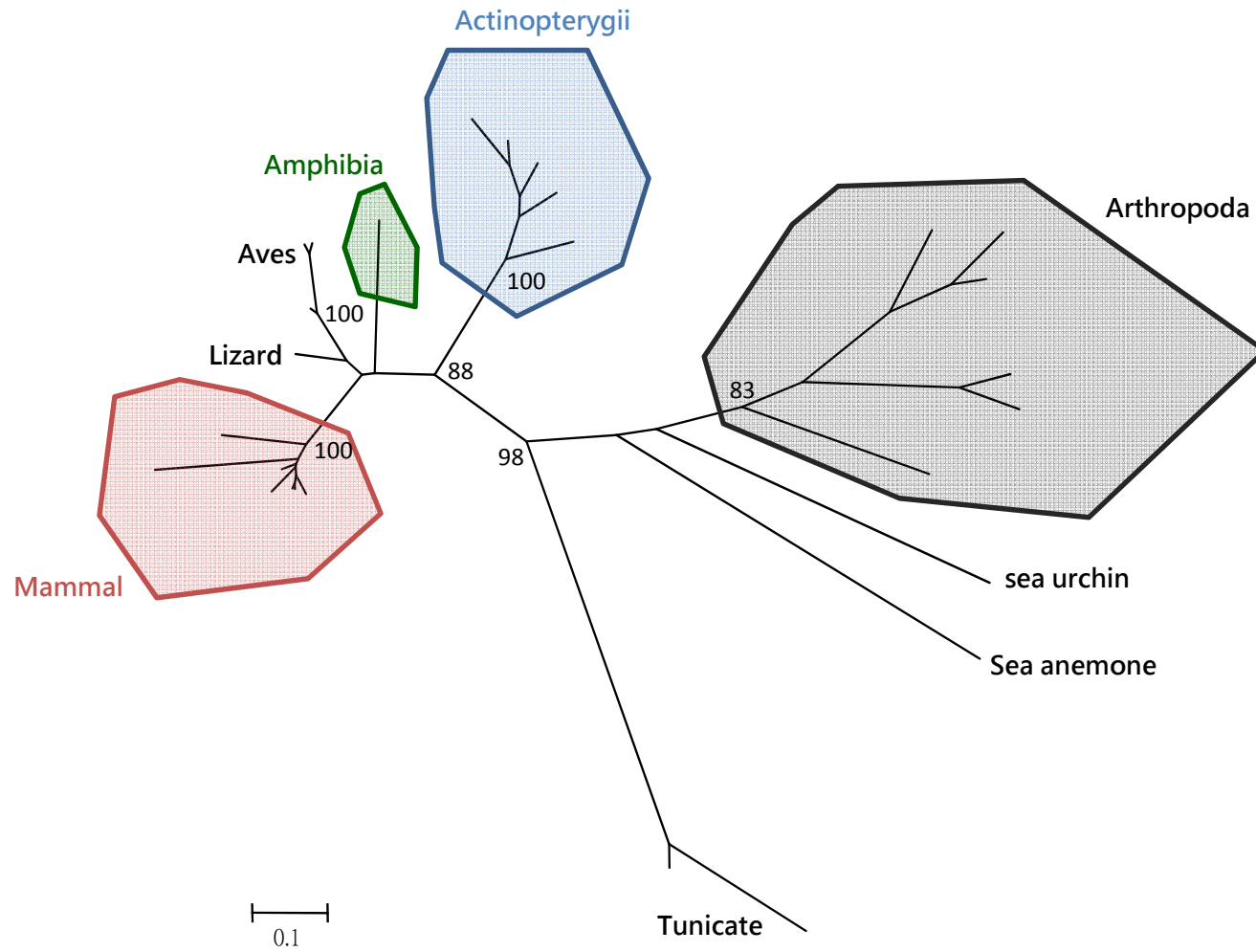

(G)

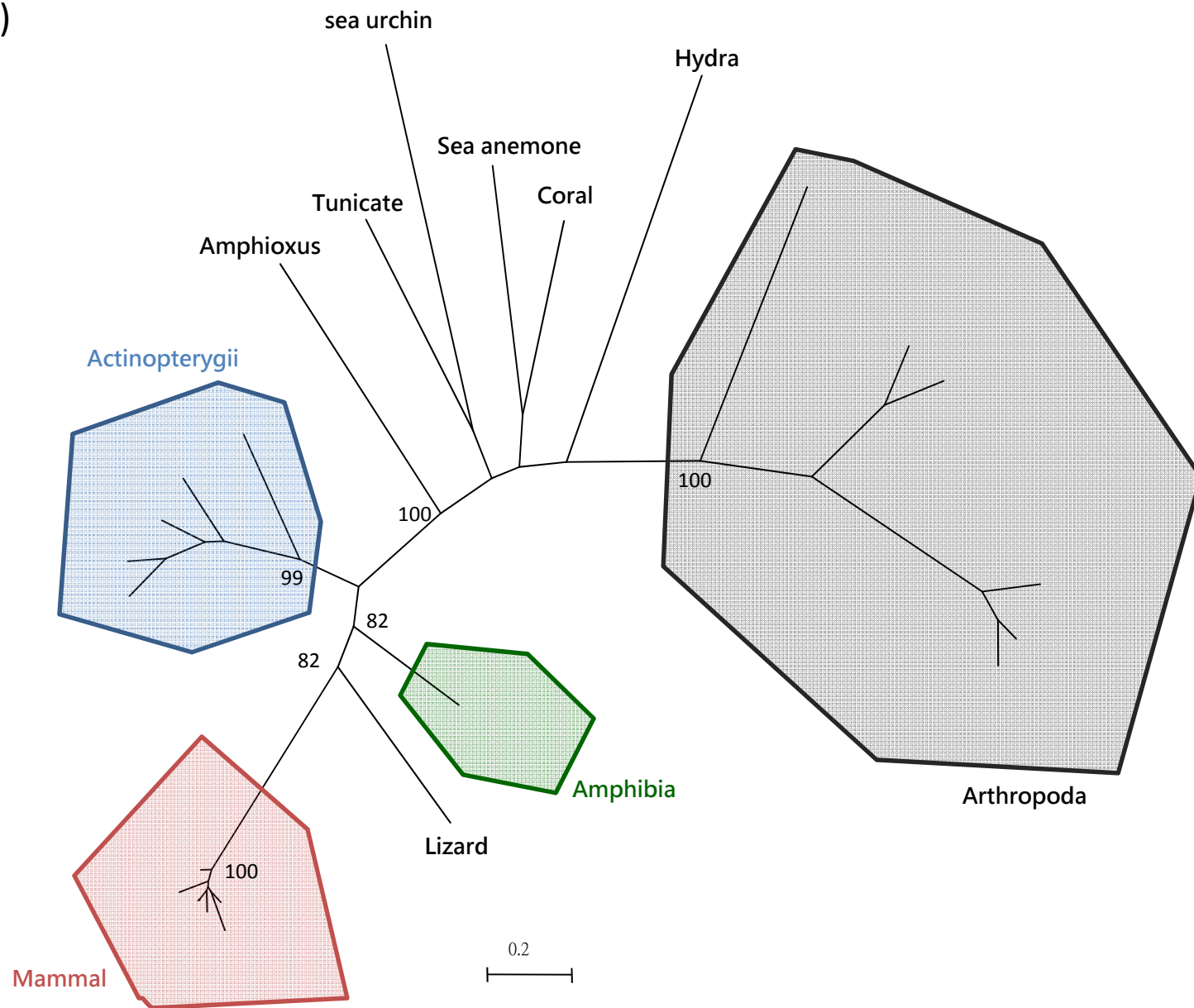

(H)

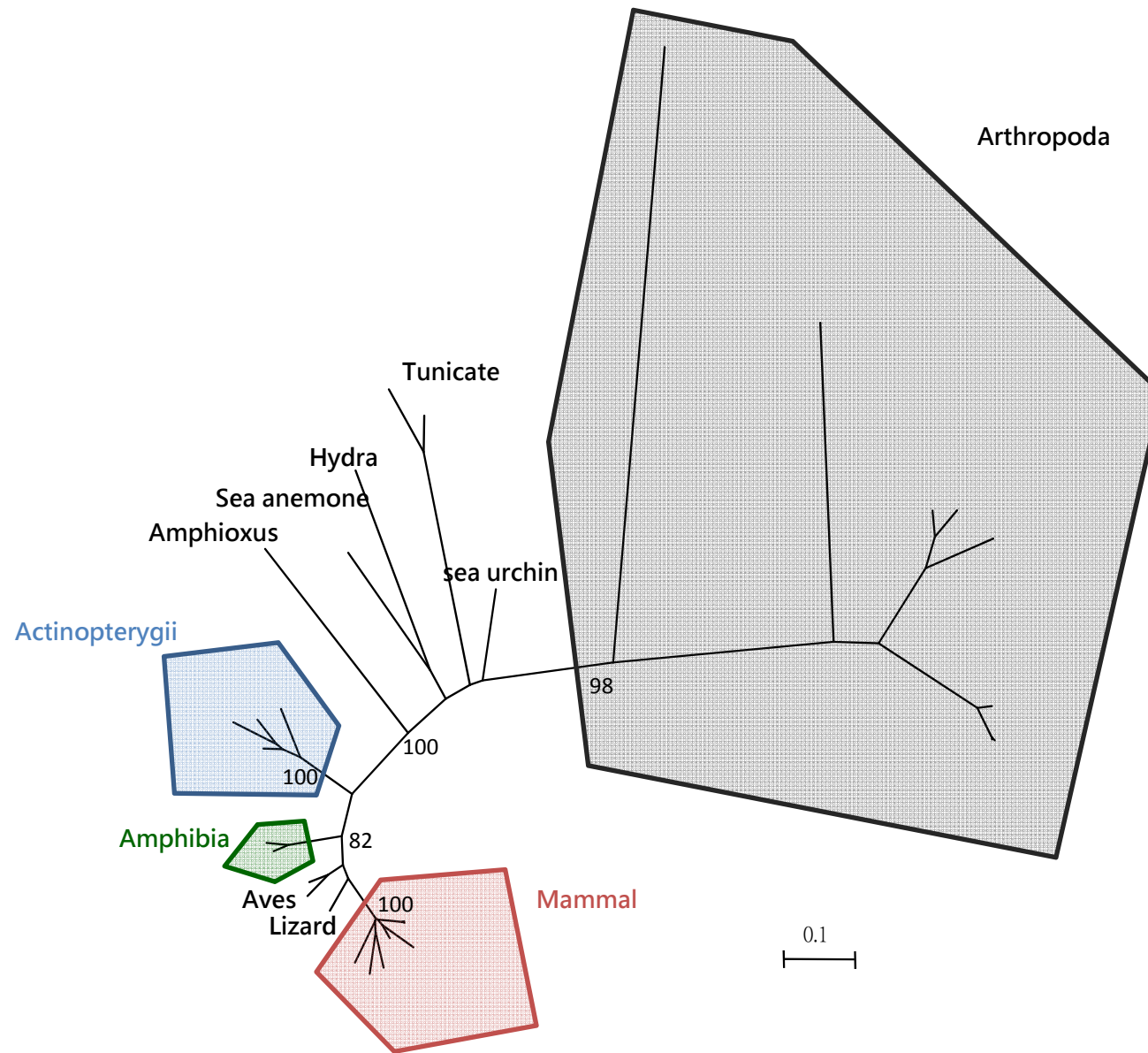

Supplement: Figure S3 — The collection of figures for the maximum likelihood phylogeny of protein sequences for eight genes of heme biosynthesis pathway in animals. Bootstrap values >70% are indicated. The bootstrap values are displayed only for the branches of the main lineages. (A) ALAS, (B) PBGS, (C) PBGD, (D) UROS, (E) UROD, (F) CPO, (G) PPO, (H) FECH. (PDF) [file pone.0086718.s003.pdf]
