# Supplementary material for: Molecular Evolution of Multiple-Level Control of Heme Biosynthesis Pathway in Animal Kingdom
Source: PLoS One. 2014 Jan 28;9(1):e86718. doi: 10.1371/journal.pone.0086718 (PMC3904948; doi:10.1371/journal.pone.0086718)
Supplement: Figure S2 — The aligned protein sequences of eight genes of heme biosynthesis pathway in animals. (PDF) [file pone.0086718.s002.pdf]

FIG. S2

#ALAS#
>HsAS1
STFQYDRFFEKKIDEKKNDHTYRVFKTVNRRAHI--FPMADDYSDSLITKKQVSVWCSNDYLGMSRHPRVCGAVMDTLKQHGAGAGGTRNISGT
SKFHVDLERELADLHGKDAALLFSSCFVANDSTLFTLAKMMPGCEIYSDSGNHASMIQGIRNSRVPKYIFRHNDVSHLRELLQRSDPSVPKIVA
FETVHSMDGAVCPLEELCDVAHEFGAITFVDEVHAVGLYGARGGGIGDRDGVMPKMDIISGTLGKAFGCVGGYIASTSSLIDTVRSYAAGFIFT
TSLPPMLLAGALESVRILKSAEGRVLRRQHQRNVKLMRQMLMDAGLPVVHCPSHIIPVRVADAAKNTEVCDELMSRHNIYVQAINYPTVPRGEE
-LLRIAPTPHHTPQMMNYFLENLLVTWKQVGLELK-PHSSAECNFCRR-PL-HFEVMSEREKSYFSGLSKLVSAQA
>MmAS1
STFQYDHFFEKKIDEKKNDHTYRVFKTVNRRAQI--FPMADDYTDSLITKKQVSVWCSNDYLGMSRHPRVCGAVMETVKQHGAGAGGTRNISGT
SKFHVELEQALADLHGKDAALLFSSCFVANDSTLFTLAKMMPGCEIYSDSGNHASMIQGIRNSRVPKYIFRHNDVNHLRELLQRSDPSVPKIVA
FETVHSMDGAVCPLEELCDVAHEFGAITFVDEVHAVGLYGARGGGIGDRDGVMPKMDIISGTLGKAFGCVGGYIASTSLLIDTVRSYAAGFIFT
TSLPPMLLAGALESVRILKSSEGRALRRQHQRNVKLLRQMLMDAGLPVIHCPSHIIPVRVADAAKNTEICDELMTRHNIYVQAINYPTVPRGEE
-LLRIAPTPHHTPQMMNFFVEKLLVTWKRVGLELK-PHSSAECNFCRR-PL-HFEVMSEREKAYFSGMSKMVSAQA
>GgAS1
STFQYDQFFEKKIDEKKKDHTYRVFKTVNRKAQI--FPMADDYSDSLITKKEVSVWCSNDYLGMSRHPRVCGAVMDTLKQHGAGAGGTRNISGT
SKFHVDLEKELADLHGKDAALLFSSCFVANDSTLFTLAKMLPGCEIYSDSGNHASMIQGIRNSRVPKHIFRHNDVNHLRELLKKSDPSTPKIVA
FETVHSMDGAVCPLEELCDVAHEHGAITFVDEVHAVGLYGARGGGIGDRDGVMHKMDIISGTLGKAFACVGGYISSTSALIDTVRSYAAGFIFT
TSLPPMLLAGALESVRTLKSAEGQVLRRQHQRNVKLMRQMLMDAGLPVVHCPSHIIPIRVADAAKNTEICDKLMSQHSIYVQAINYPTVPRGEE
-LLRIAPTPHHTPQMMSYFLEKLLATWKDVGLELK-PHSSAECNFCRR-PL-HFEVMSERERSYFSGMSKLLSVSA
>DrAS1
SSFRYDEFFEKKIEEKKSDHTYRVFKTVNRRATE--FPMADDYTESLSFKRNVSVWCSNDYLGMSRHPRVVQTIMDTLGKHGSGAGGTRNISGT
SKFHVDLEHELADLHGKDAALLFTSCFVANDSTLFTLAKMMPGCEIYSDAGNHASMIQGIRNSGAKKFIFRHNDAKHLRELLEKSDPSTPKIVA
FETVHSMDGAVCPLEQLCDVAHEFGAVTFVDEVHAVGLYGPRGGGIGDRDSVMHKMDIISGTLGKAFGCVGGYIASTHALVDTVRSYAAGFIFT
TSLPPMLLSGARQSVQILKSEEGRTLRRKHQRNVTLLRQMLMDSGLPVIHCPSHIIPVRVADAEKNTEVCDIMMSRYNIYVQAINYPTVARGEE
-LLRIAPTPHHTPQMMKYFVDKLTQTWTEVGLPLK-PHSSAECNFCRQ-PL-HFEIMSEREKSYFSGLSQPISACG
>XtAS1
KTFKYDKFFEKKIDEKKNDHTYRVFKTVNRRAEV--FPMADDYSDTLISKKTVSVWCSNDYLGMSRHPKVVGAVMETLKQHGAGAGGTRNISGT
SKFHVELEHELADLHGKDAALLFSSCFVANDSTLFTLAKMLPGCEIYSDAGNHASMIQGIRNSRAPKYVFRHNDVGHLRELLKNSDPSTPKIVA
FETVHSMDGAVCPLEEMCDVSHEYGAITFVDEVHAVGLYGARGGGIGDRDGVMTKMDIISGTLGKAFGCVGGYIASTSALIDTVRSYAAGFIFT
TSLPPMLLSGAIASVRVLKSEEGQALRRQHQRNVKLMRQMLMDSGLPVVHCPSHIIPIRVSDAAKNTEICDKLMRDYNIYVQAINYPTVARGEE
-LLRIAPTPHHNPQMMSYFLDKLLTTWKSVDLELK-PHPTAECNFCRQ-PL-HFEVMSEREKSYFSGLSKMISVRA
>BtAS1
CTFQYDRFFEKKIDEKKNDHSYRVFKTVNRKAQC--FPMADDYSDSLISKKQVSVWCSNDYLGMSRHPRVCGAVIDTLKQHGTGAGGTRNISGT
SKFHVDLEQELADLHGKDAALLFSSCFVANDSTLFTLAKMMPGCEIYSDAGNHASMIQGIRNSGVPKYIFRHNDVSHLRELLQRSDPAVPKIVA
FETVHSMDGAVCPLEELCDVAHEFGAITFVDEVHAVGLYGLQGGGIGDRDGVMPKMDIISGTLGKAIGCVGGYIASTSSLIDTVRSYAAGFIFT
TSLPPMLLAGALESVRILRSTEGRTLRRQHQRNVKLMRQMLMDAGLPVVHCPSHIIPVRVADAAKNTEVCDELMTRHNIYVQAINYPTVPRGEE
-LLRIAPTPHHTPQMMSYFVDNLLATWKRVGLELK-PHSSAECNFCRR-PL-HFEMMSEREKSYFSGMSKLVSAQA
>ClfAS1
STFQYDRFFEKKIDEKKNDHTYRVFKTVNRRAHI--FPMADDYSDSLITKKQVSVWCSNDYLGMSRHPRVCGAVMDTLKQHGAGAGGTRNISGT
SKFHVDLEQELADLHGKDAALLFSSCFVANDSTLFTLARMMPGCEIYSDSGNHASMIQGIRNSRVPKYIFRHNDVSHLRELLQRSDPSVPKIVA
FETVHSMDGAVCPLEELCDVAHEFGAITFVDEVHAVGLYGARGGGIGDRDGVMPKMDIISGTLGKAFGCVGGYIASTSSLIDTVRSYAAGFIFT
TSLPPMLLAGALESVRILKSAEGRALRRQHQRNVKLMRQMLMDAGLPVVHCPSHIIPVRVANAAKNTEVCDELMSRHNIYVQAINYPTVPRGEE
-LLRIAPTPHHTPQMMNYFLENLLATWKRVGLELK-PHSSAECNFCRR-PL-HFEVMSEREKSYFSGMSKLVSAQA
>HsAS2
YVFSYDQFFRDKIMEKKQDHTYRVFKTVNRWADA--YPFAQHFSEASVASKDVSVWCSNDYLGMSRHPQVLQATQETLQRHGAGAGGTRNISGT
SKFHVELEQELAELHQKDSALLFSSCFVANDSTLFTLAKILPGCEIYSDAGNHASMIQGIRNSGAAKFVFRHNDPDHLKKLLEKSNPKIPKIVA
FETVHSMDGAICPLEELCDVSHQYGALTFVDEVHAVGLYGSRGAGIGERDGIMHKIDIISGTLGKAFGCVGGYIASTRDLVDMVRSYAAGFIFT
TSLPPMVLSGALESVRLLKGEEGQALRRAHQRNVKHMRQLLMDRGLPVIPCPSHIIPIRVGNAALNSKLCDLLLSKHGIYVQAINYPTVPRGEE
-LLRLAPSPHHSPQMMEDFVEKLLLAWTAVGLPLQ-DVSVAACNFCRR-PV-HFELMSEWERSYFGNMGPQYVTTY
>MmAS2
QAFGYDQFFRDKIMEKKQDHTYRVFKTVNRWANA--YPFAQHFSEASMASKDVSVWCSNDYLGISRHPRVLQAIEETLKNHGAGAGGTRNISGT
SKFHVELEQELAELHQKDSALLFSSCFVANDSTLFTLAKLLPGCEIYSDAGNHASMIQGIRNSGAAKFVFRHNDPGHLKKLLEKSDPKTPKIVA
FETVHSMDGAICPLEELCDVAHQYGALTFVDEVHAVGLYGARGAGIGERDGIMHKLDIISGTLGKAFGCVGGYIASTRDLVDMVRSYAAGFIFT
TSLPPMVLSGALESVRLLKGEEGQALRRAHQRNVKHMRQLLMDRGFPVIPCPSHIIPIRVGNAALNSKICDLLLSKHSIYVQAINYPTVPRGEE
-LLRLAPSPHHSPQMMENFVEKLLLAWTEVGLPLQ-DVSVAACNFCHR-PV-HFELMSEWERSYFGNMGPQYVTTY
>ClfAS2
HVFGYDQFFRHKIMEKKQDHTYRVFKTVNRWADA--YPFAQHFSEASMASKDVSVWCSNDYLGMSRHPRVLQATQETLQRHGAGAGGTRNISGT

```
SKFHVELEQELAELHQKDAALLFSSCFVANDSTLFTLAKILPGCEIYSDAGNHASMIQGIRNSGAAKFVFRHNDPDHLKKLLKESNTRTPKIVA
FETVHSMDGAICPLEELCDVAHQYGALTFVDEVHAVGLYGPQGAGIGERDGVMHKIDIVSGTLGKAFGCVGGYIASTRDLVDMVRSYAAGFIFT
TSLPPMVLSGALESVRLLKGEEGQALRRAHQRNVKHMRQLLMDRGLPVIPCPSHIIPIRVGDAALNSKICDLLLSKHGIYVQAINYPTVPRGEE
-LLRLAPSPHHSPQMMEDFVQKLLVAWTEVGLPLQ-DVSMAACNFCHR-PV-HFELMSEWERSYFGNMGPQYVTTY
>DrAS2
PTFSYDEFFTQKIVEKKKDHTYRIFKTVNRFAEV--FPFAEDYSIAGRLGSQVSVWCSNDYLGMSRHPRVVKAIGDALKKHGAGAGGTRNISGT
SNYHVALENELARLHQKDGALVFSSCFVANDSTLFTLAKMLPGCEIYSDMGNHASMIQGIRNSGAKRFIFRHNDASHLEELLSRSDPLTPKIVA
FETVHSMDGAICPLEELCDVAHKYGALTFVDEVHAVGLYGAHGAGVGERDNVMHKIDIVSGTLGKAFGCVGGYIASTAALVDTVRSFAAGFIFT
TSLPPMVLAGALESVRVLKSDEGQALRRAHQRNVKHMRQLLLDAGLPVVNCPSHIIPIRVGNAAKNSEVCDILLEKHNIYVQAINYPTVPRGEE
-LLRLAPSPFHNPIMMNYFAEKLLDVWQEVGLPLNGP-AQASCTFCDR-PL-HFDLMSEWEKSYFGNMEPRYITVA
>XtAS2
AAFGYDDFFSRRIEEKKSDHTYRLFKTVNRRADA--YPFAEDYSDLHGEKKEVSVWCSNDYLGMSRHPKVLKAIAEALEEHGAGAGGTRNISGT
SKYHVDLECELADLHNKDAALLFSSCFVANDSTLFTLAKMLPGCEIYSDAGNHASMIQGIRNSGVTKFVFRHNDPAHLEELLQKANPKTPKIVA
FETVHSMDGAICPLEEVCDIAHKYGALTFVDEVHAVGLYGTHGAGVGERDSVMHKMDIISGTLGKAFGCVGGYVASTASLIDTVRSYAAGFIFT
TSLPPMVLAGAVESVRVLKSEEGQALRRAHQRNVKHMRQLLMDAGLPVINCPSHIIPIRVGNAAINTRICDVLLSQYNIYVQAINYPTVPRGEE
-LLRLAPSPHHTPDMMNYFVESLVSAWKEVGMPLHTP-SAAECNFCHR-PL-HFDLMSEWERTYFGNMEPKYITMY
>BtAS2
HVFGYDQFFRDKIMEKKQDHTYRVFKTVNRWADA--YPFAEHFFEASVASKDVSVWCSNDYLGMSRHPRVLQATQEILQRHGAGAGGTRNISGT
SKFHVELEQELAELHQKDSALLFSSCFVANDSTLFTLAKILPGCEIYSDAGNHASMIQGIRNSGAAKFVFRHNDPDHLKKLLKKSNPETPKIVA
FETVHSMDGAICPLEELCDVAHQYGALTFVDEVHAVGLYGSRGAGIGERDGIMHKIDIISGTLGKAFGCVGGYIASTRDLVDMVRSYAAGFIFT
TSLPPMVLSGALESVRLLKGEEGQALRRAHQRNVKHMRQLLMDRGLPVIPCPSHIIPIRVGDAMLNTRICDLLLSKYGIYVQAINYPTVPRGEE
-LLRLAPSPHHSPQMMEDFVEKLLEAWTEVGLPLQ-DISIAACNFCRR-PV-HFELMSEWERSYFGNMGPQYVTTY
>XlAS2
AAFGYDDFFSRRIEEKKSDHTYRVFKTVNRRADA--YPFAEDYSDLHGEKKEVSVWCSNDYLGMSRHPRVLKAISEALQEHGAGAGGTRNISGT
SKYHVDLECELADLHNKDAALLFSSCFVANDSALFTLAKMLPGCEIYSDAGNHASMIQGIRNSGVTKFVFRHNDPAHLEELLQKADPKTPKIVA
FETVHSMDGAICPLEEMCDVAHKYGAMTFVDEVHAVGLYGTHGAGVGERDGVMHKMDIISGTLGKAFGCVGGYIASTASLIDTVRSYAAGFIFT
TSLPPMVLAGAVESVRVLKSEEGQALRRAHQRNVKHMRQLLMDAGLPVINCPSHIIPIRVGNAAINSRICDVLLSQYNIYVQAINYPTVPRGEE
-LLRLAPSPHHTPDMMTYFVESVVSAWKEVGMPLHTP-SAAECNFCHR-PL-HFDLMSEWERTYFGNMEPKYITMY
>GgAS2
DAFPYEEQFQAQLGALRRTHTYRVVTAVGRRADA--PPLGTRGT---APHTSVELWCSSDYLGLSRHPAVLRAARAALDAHGLGAGGTRNIGGT
SPLHGALERALALLHRQPRAALFSSCFAANDTALDTLARILPGCQVYSDAGNHASMIQGIRRRGVPKFIFRHNDPHHLEQLLGRSPPGVPKIVA
FESLHSMDGSIAPLEELCDVAHAYGALTFVDEVHAVGLYGARGAGIAERDGVQHKVDVVSGTLGKALGAVGGYIAGSEALVDAVRSLGPGFIFT
TALPPQRGGGALAALQVVGSAEGAALRRAHQRHAKHLRVLLRDRGLPAL--PSHIVPVRW-DAEANTRLSRALLEEHGLYVQAINHPTVPRGQE
LLLRIAPTPHHSPPMLENLADKLSECWGAVGLPREDP-PGPSCSSCHR-PL-HLSLLSPLERDQFGVRGAAAG---
>XlAS1
QTFKYDKFFEKKIEEKKNDHTYRVFKTVNRRAEV--FPMADDYSDTLISKKTVSVWCSNDYLGMSRHPKVVGAVMETLKEHGAGAGGTRNISGT
SKFHVDLEHELADLHGKDAALLFSSCFVANDSTLFTLAKMLPGCEIYSDAGNHASMIQGIRNSRVPKYVFRHNDVGHLRELLKNSDPSIPKIVA
FETVHSMDGAVCPLEEMCDVAHEYGAITFVDEVHAVGLYGARGGGIGDRDGVMNKMDIISGTLGKAFGCVGGYVASTSALIDTIRSYAAGFIFT
TSLPPMLLSGAIASVRVLKSEEGQALRRQHQRNVKLMRQMLMDSGLPVVHCPSHIIPIRVSEAAKNTEICDKLLRDYNIYVQAINYPTVPRGEE
-LLRIAPTPHHNPQMMTYFLDKLLITWKSVDLELK-PHSTAECNFCRQ-PL-HFEVMSEREKSYFTGLSKLISARA
>MgAS
PIFDYDKFFDKKIEDKLEDHSYRIFKTVNRRADA--FPFARNYSDSLYEAKQVSVWCSNDYLGMGRHPRVINTIMDTLKMYGAGAGGTRNISGT
SRFHVALEQELADLHAKDAALVFSSCFVANDSTLFTLAKMLPGCEFYSDAGNHASMIQGIRNSGVPKFIFRHNDAAHLDELLSRSDPSTPKIVA
FETVHSMDGAICPLEELCDVSXKYGAITFIDEVHAVGLYGARGGGIGDREGLMHKMDIISGTLGKAYGCVGGYVASTNTLVDTVRSYAAGFIFT
TSLPPMVLAGARESVQVLKSAEGRALRRAHQRNVIYLRQLLMDAGLPVIHCPSHIIPVLVGNAAHNTELCNELMSRHNIYVQAINYPTVPRGEE
-MLRVVVTPHHTPQMMQYFVEHLTNSWKDIGLNLR-PHASAECNYCKM-PI-HFELMSEHDQVYFDGMSRKLGIAA
>DmAS
--FPYERFFNEQIMKKKRDHSYRVFKKVNRLAGDGLFPHALEYS--ERTEKPITVWCSNDYLGMSAHPGVKRAVQDALNRHGSGAGGTRNISGN
SLHHERLESKLAELHQKEAALLFTSCFVANDSTLFTLAKLLPGCEIFSDAGNHASMIMGIRNSGVPKHIFRHNDVDHLHQLLKQTDKSVPKIVA
FETVHSMTGAICPLEELLDVAHEHGAITFIDEVHAVGLYGDHGAGVGERDGVLHKMDIISGTLGKAFGNIGGYIAGTHNLVDMIRSYAAGFIFT
TSLPPTVLCGALEAVNILASEEGRQLRHLHQRNVSYLKSLLKREGFPVEETPSHIIPIKIGDPLKSSQISNVLIEQFGHYLQSINYPTVARGQE
-KLRLAPTPFHTFEMMNALVTDLKKVWEMVDLSTNVPLSPNACMFCNSESCWHQDTSPDLECGIPNCPRLEISLAA
>LpAS
STFCYEDFFQDQIAKKKKDHSYRIFKKVNRIAGG--FPLAQEHT---DGNHPITVWCSNDYLGMSRHPEVKRAVIEAVEAHGCGAGGTRNISGN
SPFHELLEKELAQLHNKEAALLFTSCFVANDSTLFTLAKSLPGCHIFSDAGNHASMIQGIRNSGVSKHIFHHNNPEHLEELLSKVDISIPKIVA
FETVHSMTGAVCPLEKMCDVAHKYGAITFVDEVHAVGLYGEHGGGIGEXDGVMHKMDIISGTLGKAFGNIGGYIASTLPLVDTVRSYATGFIFT
TSLPPTVLKGALTSIQVLKSEEGRMLREKHQDNVRYLRKTLMNAGIPVVHCPSHIIPVHVGDPWYSTKVSNELISMHGHYVQAINYPTVPRGEE
```

```
-KLRIAPTPFHTRPMMEAFVRDLVSVWRGLKLPLRDGICEKKCEFCEK-PL-YFEHLESRVLPPCGNRFGCPQMVA
>CiAS
PSFDYDGFMEKKIQAKKDDHSYRVFKKVNRRADT--FPFAEDHT--THDQQKVSVWCSNDYLGMSRHPKVIESIVSTIGKYGAGAGGTRNISGT
SSLHQELEEELADLHAKQSALLFSSCFVANDSTLFTLAKMLPGCEIYSDAGNHASMIQGIRNSGVPKFIFRHNDPDHLEELLSKSDPSTPRIVA
FETVHSMDGAVCPLKELCDVAHKYGAITFVDEVHAVGLYGERGAGIGERDGLMDDIDIVTGTLGKAFGNVGGYIASTSSLVDTVRSYAAGFIFT
TALPPINLAGALTAVRILKSKEGIALRAKHQRNVSMMRRMLLAAGLPVVHCPSHIIPIKVGNPEKNSAIMDIMMKKYNIYVQAINYPTVPRGEE
-MLRVAATPGHTVPMMRYFVSSLRSAWAEVGLCGKESHPTETCEYCQA-PI-QFEMMSARLKLVMPMRQPILACA-
>SdAS
GHFSYNKYFNTLVDSKKADHTYRVFKRVNREAET--FPYAYDFSHVNAEGLPVTVWCSNDYLGMSRHPKVKQAVADTMAKHGTGAGGTRNISGN
TTFHEALEAKVAKLHQKDAGLVFTSCYVANDSTLYTLAKLLPGCEIYSDEGNHASMIQGIRNSQAPKFIFRHNDPTHLEEMLRKADPLTPKIVA
FETVHSMTGAICPLKEMCDVAHKYGAITFVDEVHAVGLYGRHGAGVGERDGCMADMDIVSGTLGKAFGMIGGYIVGDNALVDMVRSYAAGFIFT
TSLPPMVLAGAMASIDVLSGEEGVALRTKHQDTVSMMRGKLIAAGLPVVHCPSHIIPIHVGNPRANTNVANSLLEEHNIYVQAINSPTVPSGEE
-KLRIAPSPXHTPDMMDRFVASLSEVWAKSGLRFNTPICPRECEFCKN-PE-KFEELSSRERSFAEESCGLRVSQG
>BfAS
GTFNYDKFLKGEIEKKKKDHTYRVFKRVNRRAP---FPFAENYSEGSHPGEDVSVWCSNDYLGMSRHPKVVETIVDTVNRRGAGAGGTRNISGN
SMYHVELENELADLHQKEAALLFTSCYVANDSTLFTLARALPGCEIYSDAGNHASMIQGIKNSGQPKFIFKHNDPIHLEELLKKSDPATPKIVA
FETVHSMDGAVCPLNELCDVAHKYGAITFIDEVHAVGLYGTRGGGIGDRDGLLPKMDIISGTLGKAFGLIGGYIAGSSLLVDMIRSYAAGFIFT
TALPPMVLAGALASVRILKSEEGVQLRRGHQRAVKLMRTMLMDAGLPVVHCPSHIIPVKVGDAEKNTDVCNDMMTKHKIYVQAINYPTVPRGEE
-MLRIAPSPHHTPEMMKYFVDALAKEWKEHDLAFE-KHSDVECHFCKM-PI-HFEIMSAREKEYFADFAQSNNRVT
>SpAS
GHFSYNKYFKNLVDSKKADHTYRVFKRVNREAES--FPYAYDFSHVNAEGLSVTVWCSNDYLGMSRHPKVKQAVVDTMAKHGTGAGGTRNISGN
TTFHEALEAKVAKLHQKDAGLVFTSCYVANDSTLYTLAKLLPGCEIYSDEGNHASMIQGIRNSQAPKFIFRHNDPTHLEEMLRKADPLTPKIVA
FETVHSMTGAICPLKEMCDVAHKYGAITFVDEVHAVGLYGRHGAGVGERDGCMADMDIVSGTLGKAFGMIGGYIVGDNALVDMVRSYAAGFIFT
TSLPPMVLAGAMASIDVLSGEEGVALRTKHQDTVSMMRGKLIAAGLPVVHCPSHIIPIHVGNPRANTNVANSLLEEHNIYVQAINSPTVPSGEE
-KLRIAPSPFHTPDMMDQFVASLSEAWAKSGLRFNTPVCPRECEFCKN-PE-KFEELSSRERSFAEESCGLRVSQG
>NvAS
AHFEYEKFFEGEIQKKKADDTYRVFKKVNRLAKA--FPHAQEFS--GAANKDISVWCSNDYLGMSRHPEVLKVARETIDVHGVGAGGTRNISGT
SSYHEQLEKVLAQLHQKEAALLFTSCYVANDTTLYTLGKHLPGCLIFSDAGNHASMIQGIRNSGAKKFIFRHNDPEHLGKLLAAADPAAPKIVA
FETVHSMTGDISPIKQLCDVAHKYGALTFVDEVHAVGLYGHHGGGVGERDGLLADMDIISGTLGKAFGVIGGYIAGTASLIDTIRSYGAGFIFT
TALPPITVKSAIKSIEILSGDEGRDLRRVHQSVVARLRNKLVQRGLPVVYAPSHIIPIHVGDAGKCTQVSNDLMSKHNIYAQAINYPTVAKGEE
-RLRIAPTPMHTEPMMEHFADVLTQVWLDNGLELLNQTCSNSCD-CQE-RCLKIDELVCNKYATPVQA--------
>MmuAS1
STFQYDRFFEKKIDEKKNDHTYRVFKTVNRRAHI--FPMADDYSDSLITKKQVSVWCSNDYLGMSRHPRVCGAVMDTLKQHGAGAGGTRNISGT
SKFHVDLEQELADLHGKDAALLFSSCFVANDSTLFTLAKMMPGCEIYSDSGNHASMIQGIRNSRVPKYIFRHNDVSHLRELLQRSDPSVPKIVA
FETVHSMDGAVCPLEELCDVAHEFGAITFVDEVHAVGLYGARGGGIGDRDGVMPKMDIISGTLGKAFGCVGGYIASTSSLIDTVRSYAAGFIFT
TSLPPMLLAGALESVRILKSAEGRVLRRQHQRNVKLMRQMLMDAGLPVVHCPSHIIPVRVADAAKNTEVCDELMSRHNIYVQAINYPTVPRGEE
-LLRIAPTPHHTPQMMNYFLENLLVTWKQVGLELK-PHSSAECNFCRR-PL-HFEVMSEREKSYFSGLSKLVSAQA
>MmuAS2
YVFSYDQFFRDKIMEKKQDHTYRVFKTVNRWADA--YPFAQHLSEASVASKDVSIWCSNDYLGMSRHPQVLQATRETLQRHGAGAGGTRNISGT
SKFHVELEQELAELHQKDSALLFSSCFVANDSTLFTLAKILPGCEIYSDAGNHASMIQGIRNSGAAKFVFRHNDPDHLKKLLEKSNPKIPKIVA
FETVHSMDGAICPLEELCDVSHQYGALTFVDEVHAVGLYGSRGAGIGERDGIMHKIDIISGTLGKAFGCVGGYIASTRDLVDMVRSYAAGFIFT
TSLPPMVLSGALESVRLLKGEEGQALRRAHQRNVKHMRQLLMDRGLPVIPCPSHIIPIRVGNAALNSKLCDLLLSKHGIYVQAINYPTVPRGEE
-LLRLAPSPHHSPQMMEDCVEKLLLAWTEVGMPLQ-DVSVAACNFCRR-PV-HFELMSEWERSYFGNMGPQYVTTY
>MgaAS
STFQYDQFFEKKIDEKKKDHTYRVFKTVNRKAQI--FPMADDYSDSLITKKEVSVWCSNDYLGMSRHPRVCGAVMDTLKQHGAGAGGTRNISGT
SKFHVDLEKELADLHGKDAALLFSSCFVANDSTLFTLAKMLPGCEIYSDSGNHASMIQGIRNSRVPKHIFRHNDVNHLRELLKKSDPSTPKIVA
FETVHSMDGAVCPLEELCDVAHEHGAITFVDEVHAVGLYGARGGGIGDRDGVMHKMDIISGTLGKAFGCVGGYISSTSALIDTVRSYAAGFIFT
TSLPPMLLAGALESVQTLKSAEGQVLRRQHQRNVKLMRQMLMDAGLPVVHCPSHIIPIKVADAAKNTEICDKLMSQHSIYVQAINYPTVPRGEE
-LLRIAPTPHHTPQMMSYFLERLLATWKDVGLELK-PHSSAECNFCRR-PL-HFEVMSERERSYFSGMSKLVSVSA
>TgAS1
STFQYDQFFEKKIDEKKKDHTYRVFKTVNRKAQI--FPMADDYTDSLITKKEVSVWCSNDYLGMSRHPRVCGAVMETLKQHGAGAGGTRNISGT
SKFHVDLEKELADLHGKDAALLFSSCFVANDSTLFTLAKMLPGCEIYSDSGNHASMIQGICNSRVPKHIFRHNDVNHLRELLKKSDPSTPKIVA
FETVHSMDGAVCPLEELCDVAHEHGAITFVDEVHAVGLYGARGGGIGDRDGVMHKMDIISGTLGKAFGCVGGYISSTSSLIDTVRSYAAGFIFT
TSLPPMLLAGALESVRTLKSAEGQALRRQHQRNVKLMRQMLMDAGLPVVHCPSHIIPVRVADAAKNTEICDKLMSQHSIYVQAINYPTVPRGEE
-LLRIAPTPHHTPQMMSYFIEKLLATWKDVGLELK-PHSSAECNFCRR-PL-HFEAMSERERAYFSGMSKLVSVSA
>AlAS1
STFQYDQFFEKKIDEKRRDHTYRVFKTVNRRAQI--FPMADDFSESHSSKKEVSVWCSNDYLGMSRHPRVCGAVMETLKQHGAGAGGTRNISGT
```

SKFHVDLEKELADLHGKDAALLFSSCFVANDSTLFTLAKMLPGCEIYSDAGNHASMIQGIRNSRVPKHIFRHNDVSHLRELLKKSDPSTPKIVA
FETVHSMDGAVCPLEELCDVSHEYGAITFVDEVHAVGLYGTHGGGIGDRDGVMHKMDIISGTLGKAFGCVGGYIASTSSLIDTVRSYAAGFIFT
TSLPPMLLAGALESVKTLKSPEGQVLRRQHQRNVKLMRQMLMDAGLPVVHCPSHIIPIRVADAAKNTEICDKMMSQHSIYVQAINYPTVPRGEE
-LLRIAPTPHHTPQMMNYFIEKLLATWKKVGLELK-PHSSAECNFCRR-PL-HFEVMSERERSYFSGMSKLVSATA
>OlAS1
SRFQYDDFFEKKIEEKKSDHTYRVFKTVNRLATE--FPMADDFTGSLEDKREVSVWCSNDYLGMSRHPQVVQSIMDTLQKHGSGAGGTRNISGT
SKFHVELEQELADLHKKDAALLFTSCFVANDSTLFTLAKMLPGCEIYSDGGNHASMIQGIRNSGAKKFVFRHNDVAHLRELLQKGDPSKPKIVA
FETVHSMDGAVCPLEEMCDLAHEFGAITFVDEVHAVGLYGARGGGIGDRDGIMHKMDIISGTLGKAFGCVGGYIASTSALVDTVRSYAAGFIFT
TSLPPMLLAGARQSIQILKGEEGRTLRRKHQRNVKLLRQMLMDSGLPVVHCPSHIIPIRVSNAEKNTEVCDIMMSRHNIYVQAINYPTVARGEE
-LLRIAPTPHHTPEMMKYFVERLVQTWKEVGLDLK-PHSSAECTFCQQ-PL-HFELMSEREKSYFSGLSHPISACA
>TrAS1a
SCFKYDAFLEKKIVEKKSDHTYRVFKTVNRLATE--FPMADDFTVSLDDKREVSVWCSNDYLGMSRHPRVMQAIMDTLRKHGSGAGGTRNISGT
SKFHVELEHELADLHKKDAALLFTSCFVANDSTLFTLAKMLPGCEIYSDAGNHASMIQGIRNSGAKKFIFRHNDVGHLRELLQKGDPTKPKIVA
FETVHSMDGAVCPLEEMCDTAHEFGAITFVDEVHAVGLYGPRGGGIGDRDGVMDKMDIISGTLGKAFGCVGGYIASTAALVDAMRSYAAGFIFT
TSLPPMLLAGARQSIQVLKSEEGRSLRRNHQRNVKLLRQMLMDSGLPVVHCPSHIIPVRVSDAEKNTKVCDLMMSRHSIYVQAINYPTVARGEE
-LLRIAATPHHTPEMMKYFVERLVDTWKEVGLELK-PHSSAECNFCQQ-PL-HFELMSEREKSYFSGLSHPISARA
>TrAS1b
VTFKYDEFFEKMIESRRSERTYRVFKTLNRRALS--FPMADSYTDSFRTKTEVSVWCSNDYLGMSRHPGVTRAIMDTLQMHGAGAGGTRNISGT
SRFHRELEYDLADLHGKDAALLFSSCFVANDSTLFTLAKMLPGCEIYSDAGNHASIIQGVRNSAARKFIFRHNDVGHLHELLEKSHPSAPKIVA
FESVHSMAGDVCPVEEMCDVAHKFGAITFVDEVHAVGLYGPRGGGIGDRDRVTHKMDIISGTLGKAFGCVGGYIASTAALVDTVRSYAAGFIFT
TSLPPMLLAGAKESVRVLKGEEGRTLRRTHQRNVKLLRQMLMDSGLPVTPCPSHIIPVHVADAEKNTQICDIMMSRYNIYVQAINHPTVAKGEE
-LLRIAPTPHHTPQMMAVFVDRLVQTWTAVGLPLR-PRSSAECHFCQQ-PL-HFELMSEREKSFFSGLGHLIPAVA
>GaAS1a
SRYQYDEFFERKIEEKKSDHTYRVFKTVNRLAQE--FPMADNFTASLEEKSDVSVWCSNDYLGMSRHPRVVQSIMETLQKHGSGAGGTRNISGT
SKFHVELEQELADLHKKDAALLFTSCFVANDSTLFTLAKMLPGCEIYSDAGNHASMIMGIRNSGAKKVVFRHNDVAHLREVLQKGDPTKPKIVA
FETVHSMDGAVCPLEEMCDLAHEFGAITFVDEVHAVGLYGARGGGIGDRDGIMHKMDIISGTLGKAFGCVGGYIASTASLVDTVRSYAAGFIFT
TSLPPMLLNGARNSIQILKGQEGRTLRRKHQRNVKLLRQMLMDSGLPVVHCPSHIIPIRVADAEKNTEVCDIMMSRHNIYVQAINYPTVARGDE
-LLRIAPTPHHTPEMMKYFVERLVDTWKEVGLEVK-PHSSAECTFCQQ-PL-HFEVMSDREKSYFSGLSHPISACA
>GaAS1b
VTFQYDKYFEEKVEGKRSDHTYRVFKTVNRSATS--FPMADDYSESLRAKRDVSVWCSNDYLGMSRHPRVTQAIEETLQKHGAGAGGTRNISGT
SRFHVHLEDELADLHNKDAALLFTSCFVANDSTLFTLAKILPGCEIYSDAGNHASMIQGIRNSGVKKLVFRHNDVNHLHELLERSDPSTPKIVA
FETVHSMDGAVCPLEEMCDIAHKFGAITFVDEVHAVGLYGPRGGGIGDRDKVMYKMDIISGTLGKAFGCVGGYIASTDALVDTVRSYAAGFIFT
TSLPPMLLAGARESIRVLKSEEGQVLRRKHQRSVKLLRQMLTDSCLPVVRCPSHIIPIRVADPEKNNEICDIMMSRYNIYVQAINYPTVAKGEE
-LLRIAPTPHHTPQMMFYFVDRLVKTWKEVGMQLS-RRSSAECDLCRR-PL-HLELMSEREKSYFTGLSHVISAVA
>AlAS2
-TFGYDAFFEAKIQAKKKDHTYRVFKTVNRRADA--YPFADDYSSSQVESQEVSVWCSNDYLGMSRHPRVLQAIKETLQLYGAGAGGTRNISGT
SKFHVDLEQELASLHAKDAALLFSSCFVANDSTLFTLARIMPGCEIYSDAGNHASMIQGIRNSGVPKYVFRHNDPQHLEELLSKAKPDLPKIVA
FETVHSMDGAICPLEEMCNVAHRYGAITFVDEVHAVGLYGTHGAGIGERDGVMGKIDIISGTLGKAFGCVGGYIASTASLIDTVRSYAAGFIFT
TSLPPMVLAGALESVRTLKSPEGQSLRRAHQRNVKHMRQLLMDAGLPVVSCPSHIIPIRVGDAALNTRLCDLLLSKYNIYVQAINYPTVPRGEE
-LLRLAPSPHHTPPMMDYFVEKLLSAWQEVELPLQAP-ASPECNFCRR-PL-HFALMSEWERDYFGNMGPRYVTIS
>OlAS2
PSYDYDHFFTEKISEKKKDHTYRVFKTVNRRANT--FPFAEDYSIPERAGAQVSVWCSNDYLGMSRHPKVLGAIREALENYGAGAGGTRNISGT
SNFHVALERELAQLHGKDAALVFSSCFVANDSTLFTLAKMLPGCEIYSDAGNHASMIQGIRNSGAKRFIFRHNDRRHLEELLQRSDPKTPKIVA
FETVHSMDGAICPLEELCDAAHRYGALTFVDEVHAVGLYGAHGAGVGERDNIMHKIDIVSGTLGKAFGCVGGYVASSGALVDTVRSYAAGFIFT
TALPPMVLAGALESVRILKSAEGQVLRRAHQRNVKHMRQLLMDKGLPVVNCPSHIIPIRVGNAELNTKVCDILLEKYNIYVQAINYPTVPRGEE
-LLRLAPSPHHEPDMMEYFVGKLVEVWQEAGLPLNSP-ATAACTFCDR-PL-HFDLMSEWEKSYFGNMEPQYITVY
>TrAS2
HGFDYDSFFIEKIAEKKQDHTYRVFKTVNRSAQA--FPFAEDYSVPGRDVPQVSVWCSNDYLGMSRHPQVLGAIREALERHGAGAGGTRNISGT
SNYHVSLERELAQLHQKDAALVFSSCFVANDSTLFTLAKMLPGCEIYSDAGNHASMIQGIRNSGAKRVIFRHNDSKHLEELLQRADPKTPKIVA
FETVHSMDGAICPLEELCDIAHRYGALTFVDEVHAVGLYGAHGAGVGERDNVMHKIDIVTGTLGKAFGCVGGYIASSAALVDTVRSFAAGFIFT
TSLPPMVLSGALESVRVLKSPEGQALRRAHQRNVKHMRQLLMDKGLPVVNCPSHIIPIRVGNAEMNTTLCNSLLERHNIYVQAINYPTVPRGEE
-LLRLAPSPHHDPAMMEYFVDKLVEVWQEAGLPLQNP-TAASCTFCDR-PL-HFDLMSEWERSYFGNMEPQYITVA
>GaAS2
PSYDYDRFFTEKISEKKKDHTYRVFKTVNRSAKV--FPFAQDYSVPGRENSQVSVWCSNDYLGMSRHPRVLGAIRNAVDNHGAGAGGTRNISGT
SNFHVTLERELAQLHQKDAALAFSSCFVANDSTLFTLAKMLPGCEIYSDAGNHASMIQGIRNSQAKRFIFRHNDSQHLEELLQRSDPNTPKIVA
FETVHSMDGAICPLEELCDVAHRHGALTFVDEVHAVGLYGAHGAGVGERDNVMHKMDIVSGTLGKAFGCFGGYIASSSALVDTVRSFAAGFIFT
TALPPMILAGALESIRFLRSRKGQVLRRSHQRNVKHMRQLLMDKGLPVVNCPSHIIPIQVGNAELNTKVCDTLLEKHNIYVQAINFPTVPRGQE

-LLRLAPSPHHNPAMMEYFVEKLVEVWHDVGLLVNGS-ATASCNFCDR-PL-HFDLMTEWEKSYFGNMEPQYITVQ
>TnAS
ASFDYDAFFMEKLGEKKQDHTYRVFKTINRSAQA--FPFAEDYSVPGRDASQVSVWCSNDYLGMSQHPRVITAIRDTLDRHGAGAGGTRNISGT
SNYHVSLETELAQLHQKDAALVFSSCFVANDSTLFTLAKMLPGCEIYSDAGNHASMIQGIRNSGAKRFIFRHNDSKHLEELLQRADPKTPKIVA
FETVHSMDGAICPLEELCDIAHRFGALTFVDEVHAVGLYGAHGAGVGERDNVMDKIDIVTGTLGKAFGCVGGYIASSAALVDTVRSYAAGFIFT
TALPPMVLSGALESVRVLKSPEGQALRRAHQRNVKHMRQLLMDKGLPVLNCPSHIIPIRVGNAELNTKVCDSLLERHNIYVQAINYPTVPRGEE
-LLRLAPTPHHNPAMMEYFVEKLVEVWQEAGLPLKSP-TVASCTFCDR-PL-HFALMSEWERSYFGNMEPQYIT--
>OcAS1
STFQYDRFFEKKIDEKKNDHTYRVFKTVNRRAHI--FPMADDYSDSLITKKQVSVWCSNDYLGMSRHPRVCAAVMDTLKQHGAGAGGTRNISGT
SKFHVDLERELADLHGKDAALLFSSCFVANDSTLFTLAKMMPGCEIYSDSGNHASMIQGIRNSRVPKYIFRHNDAGHLRELLRRADPALPKIVA
FETVHSMDGAVCPLEELCDVAHEFGAITFVDEVHAVGLYGARGGGIGDRDGVMPKMDIISGTLGKAFGCVGGYIASTSSLIDTVRSYAAGFIFT
TSLPPMLLAGALESVRILKSAEGRALRRQHQRNVKLMRQMLMDAGLPVVHCPSHIIPVRVADAAKNTAVCDELMSRHNIYVQAINYPTVPRGEE
-LLRIAPTPHHTPQMMSYFLENLLATWKQVGLDLK-PHSSAECNFCRR-PL-HFEVMSEREKSYFSGMSKLVSAQA
>LaAS1
STFQYDHFFEKKIDEKKNDHTYRVFKTVNRRAHI--FPMADDYSDSLITKKQVSVWCSNDYLGMSRHPRVCGAVMDTLKQHGAGAGGTRNISGT
SKFHVDLEQELADLHGKDAALLFSSCFVANDSTLFTLAKMMPDCEIYSDSGNHASMIQGIRNSRVPKFIFRHNDVSHLRELLQRSDPSVPKIVA
FETVHSMDGAVCPLEELCDVAHEFGAITFVDEVHAVGLYGARGGGIGDRDGVMPKMDIISGTLGKAFGCVGGYIASTSSLVDTVRSYAAGFIFT
TSLPPMLLAGALESVRILKSSEGRALRRQHQRNVKLMRQMLMDAGLPVVHCPSHIIPVRVADAAKNTEVCDELMSRRNIYVQAINYPTVPRGEE
-LLRIAPTPHHTPQMMSYFLENLLATWKRVGLELK-PHSSAECNFCRR-PL-HFEVMSEREKSYFSGMSKLVSAQA
>HmAS
SAFEFDGFFENMIQKKLDDHSYRVFRKVNRDANK--FPLGEDFS-FSDEPKPVTVWCSNDYLGMSRHKVVVEAAKNVIESNGVGAGGTRNISGT
STYHSMLENSLAKWHKKEAGLLFTSCYVANDTALFTLGQQLPGCIFFSDAGNHASMIHGIRTSGAKKVIYRHNDPEHLEQLLKTSDPSAPKIVV
FETVHSMSGSVCPLEELCDIAHEYNALTFVDEVHAVGLYGKHGAGIGERDNCMHKMDIISGTLGKAIGCIGGYLVGNSKVIDTLRSYGSGFIFT
TALPPDKVYAAYKSIEVLKTKEGQALRQKHQANVRQLKTKLIQRGFPVHPSPSHIIPVMAADPDKCTKISQLLQSHHGIYVQSINYPTVPRGQE
-KLRIAPTPFHTEQMMDELVDALTEVWLEVGLPIQYPLCNVECE-CQD-MC-KQGTLA---YTHVAAA--------
>DaAS
ETFPYEKFFNEQIQKKKKDHSYRIFKKVNRLAGDGLFPHALEYS--ERSEKPITVWCSNDYLGMSAHPSVKRAVQEALNMHGSGAGGTRNISGN
SLHHERLERKLAELHQKEAGLLFTSCFVANDSTLFTLARLLPGCQIFSDAGNHASMIMGIRNSGVPKHIFRHNDVDHLRSLLKKVDKSTPKIVA
FETVHSMTGAICPLEELLDVAHEYGAITFIDEVHAVGLYGDHGAGVGERDGVLHKMDIISGTLGKAFGNIGGYIAGSEKLVDMIRSYAAGFIFT
TSLPPTVLCGALEAVNILASEEGRELRHLHQRNVSYLKNLLKREGFPVEETPSHIIPIKIGDPLKSSQISNVLMEQFGHYLQSINYPTVARGEE
-KLRLAPTPFHTYAMMNALVTDLKKVWEMVDLSTNVPLSPKACMFCNSESCWHQDASPDLECGIPNCPRLEISVAA
>DpAS
DTFPYEKFFNEQIMKKKRDHSYRVFKKVNRLAGDGLFPHALEYS--ERTERPITVWCSNDYLGMSAHPSVKRAVQDALNMHGSGAGGTRNISGN
SLHHERLEEKLADLHQKEAALLFTSCFVANDSTLFTLAKLLPGCHIFSDAGNHASMIQGIRNSGVPKHIFRHNDVDHLRILLQQLDKSTPKIVA
FETVHSMTGAICPLEELLDVAHEYGAITFIDEVHAVGLYGDHGAGVGERDEVLHKMDIISGTLGKAFGNIGGYIAGSDKLVDMIRSYAAGFIFT
TSLPPTVLCGALEAVNVLASDEGRHLRSLHQRNVSYLKNLLKREGFPVEDTPSHIIPIKLGDPLKCTQISNMLIEQFGHYLQSINYPTVARGQE
-KLRLAPTPFHTFEMMNALVTDLKKVWQMVDLSTNVPLSPNGCMFCNTESCGHQDTCPDLECGIPNCPRLEISVAA
>AmAS
-SFPYEEFFHEQIMKKKKDHSYRVFKKVNRLAEN--FPTAIEYS---WGEKPITVWCSNDYLGMSRHPTVIHSVREALDKFGTGAGGTRNISGN
SMGHEILEKRLASLHQKEAGLLFTSCFVANDSTLYTLAKLLPHCHIFSDAGNHASMIQGIRNSGVPKHIFRHNDVQHLEELLSKVDKNIPKIVA
FETVHSMTGDICPLEDLCDIAHKYNALTFVDEVHAVGLYGYSGAGIGERDWVLHKMDIISGTLGKAFGNVGGYIVGSAKLIDIIRSYAAGFIFT
TSLPPTVLYGALTAIEILASDEGRLLRANHQKNVAYMKSILSIANLPLEQSPSHIIPIKIGDPLLCSQLADLLIKDKGHYVQAINYPTVPKGEE
-KLRLAPTPKHTQTMMDQFVKDILHVFHRLNIPIKHKSNNIPCVIQVH----------------------------
>AaAS
RPFQYEEFFHEQIMRKKKDHSYRVFKKVNRLAEAGKFPKALEYS---WGERPITVWCSNDYLGMSCHPEVKKAVANALETYGAGAGGTRNISGN
SLNHENLESRLAQLHEKEGALLFTSCFVANDSTLFTLAKALPGCHIFSDAGNHASMIQGIKNSGVPKHIFRHNDPAHLRELLSKVDKSLPKIVA
FETVHSMTGAICPLEELCDVAHEFGALTFVDEVHAVGLYGDHGAGVGEREGLLHKMDIISGTLGKAFGNVGGYIASTSLLIDMVRSYAAGFIFT
TSLPPTVLCGALKAVNILASDEGRELRTRHQENVRYLRGLLQREGFPVEHTPSHIIPVKIGNPAQCTELSDLMIKRFGHYIQAINYPTVARGEE
-KLRLAPTPHHTKEMMDILVRDMKIVWKDLGMPLNGSACTAECTFCRK-PL-LFDRFESRTKASCAAEINCQIPNC

#PBGS#
>HsPS
QSVLHSGYFHPLLRAWQTATTTLNASNLIYPIFVTDVPDDIQPITSLPGVARYGVKRLEEMLRPLVEEGLRCVLIFGVPSRVPKDERGSAADSE
ESPAIEAIHLLRKTFPNLLVACDVCLCPYTSHGHCGLLSENGAFRAEESRQRLAEVALAYAKAGCQVVAPSDMMDGRVEAIKEALMAHGLGNRV
SVMSYSAKFASCFYGPFRDAAKSSPAFGDRRCYQLPPGARGLALRAVDRDVREGADMLMVKPGMPYLDIVREVKDKHPDLPLAVYHVSGEFAML
WHGAQAGAFDLKAAVLEAMTAFRRAGADIIITYYTPQLLQWLKEE
>MmPS

QSVLHSGYFHPLLRSWQTAASTVSASNLIYPIFVTDVPDDVQPIASLPGVARYGVNQLEEMLRPLVEAGLRCVLIFGVPSRVPKDEQGSAADSE
DSPTIEAVRLLRKTFPSLLVACDVCLCPYTSHGHCGLLSENGAFLAEESRQRLAEVALAYAKAGCQVVAPSDMMDGRVEAIKAALLKHGLGNRV
SVMSYSAKFASCFYGPFRDAAQSSPAFGDRRCYQLPPGARGLALRAVARDIQEGADMLMVKPGLPYLDMVREVKDKHPELPLAVYQVSGEFAML
WHGAQAGAFDLRTAVLETMTAFRRAGADIIITYFAPQLLKWLKEE
>DrPS
ESILHSGYFHPTLRYWQTCASELRPDNLIYPIFITDSPDAVEPIASLPGQARYGVNKIEGLLRPLVDKGLKCVLIFGVPAKVAKDERGSGADAD
DTPAVLAVKKLRSTFPELVLACDVCLCPYTSHGHCGILREDGSLDNAASCLRLAEVALAYARAGCHIIAPSDMMDGRIAAIKQALIANDLGNKV
SVLSYSAKFASCYYGPFRDAAQSKPAFGDRRCYQLPPGARGLALRACDRDVKEGADMLMVKPGLPYLDIVREVKNKHPTHPLAVYNVSGEFAML
WHGAEAGAFDLRTAVMEAMTAFRRAGADIIITYYTPQLLIWLTE-
>XtPS
SAILHSGYFHPVLRAWQCSATSLDANNLMYPIFITDNPDAIEEIPSLPGQARYGVNQLEGLLRPLVDNGLKCVLIFGVPSKVIKDERGSAADAP
DTPAILAIQRIREKFPQLLIACDVCLCPYTSHGHCGILREDGSLQNESSCQRLAEVALAYARAGCHIVAPSDMMDGRIGAIKQALVSNDLGNKV
SVMSYSAKFASCFYGPFRDAAQSKPAFGDRKCYQLPPGARGLAIRAVDRDVREGADMLMVKPGMPYLDLVREVKEKHPALPLAVYHVSGEYAML
WHGAQANAFDLKVAVLEAMTGFRRAGADIIITYYTPQLLQWIKER
>BtPS
QSVLHSGYFHPLLRNWQTAATSLSASNLIYPIFVTDVPDDKQPIASLPGVARYGVNRLEEMLKPLVEEGLRCVLIFGVPSRVPKDERGSAADSE
DSPAIEAIRLLRKNFPSLLVACDVCLCPYTSHGHCGLLSENGSFQAEESRQRLAEVALAYAKAGCQVVAPSDMMDGRVEAIKEALMAHGFGNRV
SVMSYSAKFASCFYGPFRDAAQSSPAFGDRRCYQLPPGARGLALRAVDRDVREGADLLMVKPGTPYLDIVREVKNKHPELPLAVYHVSGEFAML
WHGAQAGAFDLKAAVLEVMTAFRRAGADVIITYYTPQLLQWLKE-
>ClfPS
QSVLHSGYFHPLLRTWQTAATTLSASNLIYPIFVTDVPDDVQPISSLPGVARYGVNRLEEMLKPLVEEGLRCVLVFGVPSRVPKDERGSAADSE
DSPAIEAIRLLRKTFPSLLVACDVCLCPYTSHGHCGLLSKNGAFLAEESRQRLAEVALAYAKAGCQVVAPSDMMDGRVEAIKEALMAHGLGNRV
SVMSYSAKFASCFYGPFRDAAQSSPAFGDRRCYQLPPGARGLALRAVARDVREGADMLMVKPGMPYLDVVREVKDKHPELPLAVYHVSGEFAML
WHGARAGAFDLKAAVLEAMTAFRRAGADIIITYYTPQLLQWLKEE
>XlPS
DSILHSGYFHPVLRAWQSTATSLDANNLMYPIFITDNSDAVEDIPSLPGQARYGVNQLDVLLRPLVDNGLKCVLIFGVPSRVTKDDRGSAADAD
DTPAILAIRRIREKFPQLLVACDVCLCPYTSHGHCGILREDGSIQNESSCQRLAEVALAYARAGCHIVAPSDMMDGRIGAIKQALISNDLGNKV
SVMSYSAKFASCFYGPFRDAAQSKPAFGDRKCYQLPPGARGLALRAVDRDVREGADMLMVKPGIPYLDLVRDVKDKHPALPLAVYHVSGEYAML
WHGAQANAFDLKVAVLEAMTGFRRAGADIIITYYTPQLLNWIKER
>CiPS
KNYLHSSIHHPTLRAWNSIPSTLNADNFMYPVFITDTSDAYEPIPSLPEQARHGVDHLVKYLAPLVQGKLKAVLVFGVPTQIKKDGCGSAADDE
MGPVIRGIKKLRKAFPNLLVAADVCLCAYTDHGHCGILNENGTINNPPSIKRLAEVAVNYAKAGAHVVAPSDMMDCRIKAIKHGLHEAGLAGCT
TVLSYSAKFSSCFYGPFRDAAQSAPSFGDRKCYQLPPGSRGLAIRAADRDVQEGADMLMVKPGMPYLDIIFEIKQKYPHHTMAAYHVSGEYAML
CHGASNGALDLKSAVLESFSSLRRAGTDVIITYFTPRVLKWLKE-
>DmPS
ERKLHSGMHHATLRQLQESGCEIAPHNLMYPVFIVSNDDDVQPIASMPGISRFGLNRLKEHLEPLVAKGLSSVLLFGVVDPDMKDEQASNADSA
KNPVVLALPKLREWFPDLLIACDVCICPYSSHGHCGLLGETG-LENGPSIKRIAEIAVAYAKAGAHIVAPSDMMDNRVKAIKQALIDAQM-NSV
SLLAYSAKFTSNFYGPFREAAQSAPKFGDRRCYQLPSGSRSLAMRAIQRDVAEGADMLMVKPGMPYLDILRSTKDSYPYHTLYVYQVSGEFAML
YHAAKAGAFDLKDAVLEAMKGFRRAGADCIITYYTPFLLDIIGKV
>MmuPS
QSVLHSGYFHPLLRAWQTATTTLNASNLIYPIFVTDVPDDIQPIASLPGVARYGVNRLEEMLRPLVEEGLRCVLIFGIPSRVPKDERGSAADSE
ESPAIEAIHLLRKTFPNLLVACDICLCPYTSHGHCGLLSENGAFRAEESRQRLAEVALAYAKAGCQVVAPSDMMDGRVEAIKEALMAHGLGNRV
SVMSYSAKFASCFYGPFRDAAQSSPAFGDRRCYQLPPGARGLALRAVDRDVREGADVLMVKPGMPYLDIVREVKDKHPDLPLAVYHVSGEFAML
WHGAQAGAFDLKAAVLEAMTAFRRAGADIIITYYTPQLLQWLKKE
>AlPS
ESLLQSGYFHPVLRSWQASASAFQASQLIYPVFVTDNPDAVEPIASLPGQARHGVNRLEELLRPLVADGLKCILMFGVPSKAIKDECGSAADGE
ETPVIQAVRRVRSLFPELLIACDVCLCPYTSHGHCGILRTDGSLQNEASCHRLAEVALAYAKAGCHIVAPSDMMDGRIRAIKEALISNDLGNKV
SVMSYSAKFASCFYGPFRDAALSKPAFGDRRCYQLPPGSRGLALRAVDRDVKEGADLLMVKPGMPYLDLVREVKDRHPNHPLAIYHVSGEFAML
WHGAQAGAFDLKAVVMEALAGFRRAGADVLITYFVPQLLQWMKED
>MgPS
DSLLHSGYFHPVLRSWQCTATTFNASNLIYPIFVTDSPDAVEPIASLPGQARYGVNKLEGMLQPLVEDGLKCVLIFGVPSKVPKDERGSAADAE
DTPAIQAIRKICSTFPQLLIACDVCLCPYTSHGHCGILREDGTIQNEASCQRLAEVALAYAQAGCHIVAPSDMMDGRIAAIKKALISNDMGNKV
SVMSYSAKFASCFYGPFRDAAQSKPAFGDRRCYQLPPGARGLALRAVDRDVREGADMLMVKPGMPYLDLVRDVKERHPTHPLAVYHVSGEFAML
WHGAQAGAFSLRAAVLPAHGCARRGGALTDSGKPLPGLLQCTQLG
>OlPS
QSVLHSGYFHQTLRFWQTCATNLRPENLIYPIFVTDAADAVEPINSLPGQARYGVNKLEEMLKPLVAKGLKCVLIFGVPAKIAKDDRGSGADTD
DTPAVLAVKKIRSLFPELLVSCDVCLCPYTSHGHCGILNDDGTLNNDASCLRLAEVALAYARAGCHIIAPSDMMDGRVGAIKQALLSNGLGNKV

SVLSYSAKFASCYYGPFRDAAQSKPAFGDRRCYQLPPGARGLALRAVERDVREGADMLMVKPGLPYLDILREVKDKFPTHPLAVYNVSGEFAMM
WHGAKAGAFDLRAAVMEAMTAFRRAGADIIITYFTPELLSWLNES
>TrPS
QSILHSGYFHPTLRYWQTCATDLKADNLIYPIFITDSPDAVEPIGSLPGQARYGVNQLEAILSPLVENGLKCVLIFGVPSKIQKDERGSGADSE
DTPAVLAVKKLRSLFPELLVACDVCLCPYTSHGHCGILNDDGTLNNDASCLRLAEVALAYAQAGCHIIAPSDMMDGRVRAIKQALLSNGVGNKV
SVLSYSAKFASCYYGPFRDAAQSKPAFGDRRCYQLPPGAGGLAIRAVERDVREGADMLMVKPGLPYLDIVRQVKDKFPNHPLAVYNVSGEFAMM
WHGAQAGAFDLRAAVMEAMTAFRRAGADIIITYYTPQLLSWLKE-
>GaPS
ESIIHSGYFHPTLRYWQTCVADLRPDNLIYPVFVTDSADAVEPIGSLPGQARYGVNKLEGMLQPLVDNGLKCVLIFGVPAKIEKDDRGSGADTD
DTPAVLAVKKIRSLFPELLVACDVCLCPYTSHGHCGILNDDGTLNNDASCLRLAEVSVAYARAGCHIIAPSDMMDGRVRAIKHALISNGMGNKA
SVLSYSAKFASCYYGPFRDAAQSKPAFGDRRCYQLPPGARGLALRAVERDVREGADMLMVKPGLPYLDIVREVKDKFPTHPLAVYNVSGEFAMM
WHGAQAGAFDLRAAVMEAMTAFRRAGADIIITYYTPQLLSWLKE-
>TnPS
QSILHSGYFHPTLRYWQTCAADLKPDNLIYPIFITDSPDAVEPIGSLPGQARYGINKIEAMLRPLVENSLKCVLIFGVPSKIQKGNRMTGADTD
DTPAVLAVKKIRSLFPELLVACDVCLCPYTSHGHCGILNDDGTLNNDASCLRLAEVAVSYAKAGCHIIAPSDMMDGRVSAIKQGLLSNGMGNKV
SVLSYSAKFASCYYGPFRDAAQSKPAFGDRRCYQLPPGAGGLAIRAVERDVREGADMLMVKPGLPYLDIVRAVKDKFPSHPLAVYNVSGEFAMM
WHGAQAGAFDLRAAVMEAMTAFRRAGADIIITYYTPQLLSWLKE-
>GgPS
DSLLHSGYFHPVLRSWQCTATTFNASNLIYPIFVTDSPDAVEPIASLPGQARYGVNKLEGMLQPLVEDGLKCVLIFGVPSKVPKDERGSAADAE
DTPAIQAIRKICSTFPQLLIACDVCLCPYTSHGHCGILREDGTIQNEASCQRLAEVALAYAQAGCHIVAPSDMMDGRIAAIKKALISNDMGNKV
SVMSYSAKFASCFYGPFRDAAQSKPAFGDRRCYQLPPGARGLALRAVDRDVREGADMLMVKPGMPYLDLVRDAKERHPTHPLAVYHVSGEFAML
WHGG-AGRRLQKAAVMEAMAAFRRAGADTIITYFTPQLLRWLRED
>CqPS
LTKLHSSIFHPTLRKLQCQDVDIAAHNLMYPIFLVEDDDAIQEIPSMPGVARYGINPLKKHLTPLVEKGLASILLFGVVEKLPKDPTGTGADSK
DNPVVKALPLLRKWFPDLLIACDVCLCPYTSHGHCGVLTEDGVIDNEPSIQRIAEISLAYARAGAQIVAPSDMMDNRIWAIKKILRENKLENRV
SVLSYSVKFASGFYGPFRDAAKSAPAFGDRKCYQLPPGSKGIARRAAKRDVEEGADMLMVKPGMAYLDIVKQVKDDYPELPLFIYQVSGEYSML
LNAGKIGAFDLRTVLWEVLVGMRRAGADCIITYFTPTLLDWLRE-
>NvPS
GAALKSGFQHAVTRQWQSTNTSLTPHNLIYPLFISDDPDGLEHIASLPGQARVGINNLEKVVKPLVDKGLSSVLVFGVISKLEKDGNASNADSD
LCPTILGIKKLHSLFPDLLIICDVCLCPFTDHGHCEVMFTGNKIYSSESCDRTANVSKFSAFVGCHVVAPSDMMDNRVAAIKQIMAANGYGSKV
AVLSYSAKFASSFYGPFRDAAKSAPAFGDRRCYQLPPGARGLAMRAVDRDVQEGADMLMVKPGMPYLDIVRDTKNKYPDLPLAIYQILA-----
-----ANGYASKVAVLSYRQFLFLLGADIIITYFVPQLLDWLQE-
>AdPS
QGSLHGGYNHSATRAWQSTNTEITSKNLIYPLFISDHDDAYEEISSLPGQYRMGVNNIEKIVSPLVKNGLKSVLLFGVVTKLQKSENGHNADSD
LSPAILATNKLRNLFPELLICCDVCLCPYTSHGHCGILNKDGSINNVESIKRLAEVSLAYAKAGCHVIAPSDMMDNRVGAIKDILHQNGFGGKV
AVMSYSAKFASSFYGPFRDAAMSAPAFGDRRCYQLPPGAKGLAARAVERDVKEGADMVMVKPGMAYLDIVRQTKDKFPDLPLAIYQVSGEYAML
YHGSKAGAFDLKTIVLESLTAMRRAGADIIITYYVPSLLDWLKEN
>OcPS
QSVLHSGYFHPLLRSWQTASTTLSASNLIYPIFVTDVPDDVQPINSLPGVARYGVNRLEEMLRPLVEEGLRSVLIFGVPSRVPKDERGSGADSE
DSPAVEAVRLVRKTFPSLLVACDVCLCPYTSHGHCGLLGENGAFQAEESRQRLAEVALAYAKAGCQVVAPSDMMDGRVEAIKEALLAHGLGNRV
SVMSYSAKFASCFYGPFRDAAQSSPAFGDRRCYQLPPGARGLALRAVDRDVREGADMLMVKPGMPYLDIVREVKVKHPELPLAVYHVSGEFAML
WHGAQAGAFDLKAAVLEAMTAFRRAGADIIITYYTPQLLQWLKEE
>HmPS
HCILQGGYFNNTLRSWQSNTSGLTAASLIYPIFI------------------RVGKNKVVDYLKPLIENGLKTVLLFGMVKEDHKNEKGDAIYIE
DNPVINVIKLLKETFHGLLIACDVCLCAYTDHGHCGVLDNKAAIDNKASVNAIALASLIYATAGCHIIAPSDMMDGRIYKIDQLLKENSLRNKV
TIMSYSAKFASCFYGPFRDAAASAPAFGDRRCYQLPPQSSGLANRSVSRDVSEGADILMVKPGMAYLDVVKEIKNKYPDYPVAVYQVSGEYAML
YHASQQGAFDIKQAVIESLHCMMRAGATVLISYFTPQVLKWLKE-
>DaPS
ERKLHSGMHHATLRLLQESGCEIAPHNLMYPLFIVSNDDDVQPIASMPGISRFGLNRLRDHLQPLVAKGLSSVLLFGVVEPELKDEEASNADSA
HNPVVRALPKLREWFPDLLIACDVCICPYSSHGHCGLLGETG-LENGPSIKRIAEIAVAYAKAGAHIVAPSDMMDNRVKAIKQALIDAEM-SRV
SLLAYSAKFSSNFYGPFREAAQSAPKFGDRRCYQLPSGSRSLAMRAIQRDVAEGADMLMVKPGMPYLDILRSTKDLYPFHTLYVYQVSGEYAML
YHAAKAGAFDLQEAVLEAMKGFRRAGADCIITYYTPFLLDVIGKV
>DpPS
DRKLHSGMHHPTLRLLQESGCEISPHNLMYPVFIVSNDDDVQPIASMPGISRFGVNRLRDHLEPLVKKGLTSVLLFGVVESELKDEEASNADSA
VNPVVRALPKLREWFPDLLIACDVCICPYSSHGHCGLLGKAG-LENGPSIKRIAEIAVAYGQAGAHIVAPSDMMDNRVRAIKQALIDASL-NSV
SLLAYSAKFASNFYGPFREAAQSAPKFGDRRCYQLPSGSRSLAMRAIQRDVSEGADMLMVKPGMPYLDILRQTKDSYPYHTLYVYQVSGEYAML
YHASKAGAFELRDAVLESMRSFRRAGADCIITYYTPYLLDIIGRT

```
>AmPS
KHTLHSGIFHPVLRQWQSPNVEITVNTLMYPIFISDEQDAKEPISSMPGVYRYGINHLRKMLQPLVLKGLQSILLFGVSKHLKKDHIGSNADSI
KNPIIKAVPLIREWFPNLLIACDVCLCPYTIHGHCGILNEDESINNKASIERISEIALAYAKAGAQIVAPSDMMDGRIGAIKKKLAAAGLMNKV
AVLSYAVKFASGFYGPFRDASQSAPKFGDRKCYQLPPGSNGLAARAAARDVAEGADMLIVKPGLPYLDVVRHTKDAHPEYPMFVYQVSGEYAML
YHGAQNGAINLENVLKEVLLSMRRAGADCIITYFTPLILDMLQPK
>AaPS
LTKLHSSIFHPTLRKLQCQDVDIAAHNLMYPVFLVEDDDAVQEIPSMPGVARYGLTTLKKHLTPLVEKGLASILLFGVVDKLPKDPTGSGADSA
ENPVVKALPQLRQWFPDLLIACDVCLCPYTSHGHCGVLTADGVIDNEPSIQRIAEIAFAYAKAGAHIVAPSDMMDNRIWAIKKILRENKMENTV
SVLSYSVKFASGFYGPFRDAAKSAPAFGDRKCYQLPPGSKGIAKRAAKRDVEEGADMLMVKPGMAYLDIVKQVKDDYPELPLFIYQVSGEYSML
LNAGKIGAFDLRTVLWEVLIGMRRAGADCIITYFTPTLLDWL---

#PBGD#
>HsPD
MSGNGNAAATAEENSPKMRVIRVGTRKSQLARIQTDSVVATLKASYPGLQFEIIAMSTTGDKILDTALSKIGEKSLFTKELEHALEKNEVDLVV
HSLKDLPTVLPPGFTIGAICKRENPHDAVVFHPKFVGKTLETLPEK-SVVGTSSLRRAAQLQRKFPHLEFRSIRGNLNTRLRKLD-EQQEFSAI
ILATAGLQRMGWHNRVGQILHPEECMYAVGQGALGVEVRAKDQDILDLVGVLHDPETLLRCIAERAFLRHLEGGCSVPVAVHTAMKDGQLYLTG
GVWSLDGSDSIQETMQATIHVPAQHEDGPEDDPQLVGITARN-IPRGPQLAAQNLGISLANLLLSKGAKNILDVARQLNDAH
>ClfPD
MSGNGTAAAAAEGNGPKMRVIRVGTRKSQLARIQTDSVVAMLKALYPGLQFEIVAMSTTGDKILDTALSKIGEKSLFTKELEHALEKNEVDLVV
HSLKDLPTVLPPGFTIGAICKRENPYDAVVFHPKFVGKTLETLPEK-SVVGTSSLRRAAQLQRKFPHLEFRSIRGNLNTRLRKLD-ELQEFSAI
ILAAAGLQRMGWQHRVGQILHPEECMYAVGQGALGVEVRAKDQDMLDLVGVLHDPETLLRCIAERAFLRHLEGGCSVPVAVHTAMKDGQLYLTG
GVWSLDGSDSMQETMQATIHITAQHEDGPEDDPQLVGITARN-IPREAQLAAENLGISLASLLLNKGAKNILDVARQLNDAH
>BtPD
MSGNGNAAAIAEEDTPKMRVIRVGTRKSQLARIQTDSVVATLKALYPGLQFEIIAMSTTGDKILDTALSKIGEKSLFTKELEHALERNEVDLVV
HSLKDLPTVLPPGFTIGAVCKRESPYDAVVFHPKFVGKTLETLPEK-SVVGTSSLRRAAQLQRKFPHLEFKSIRGNLNTRLRKLD-ELQEFSAI
ILATAGLQRMGWQNRVGQILHPEECMYAVGQGALGVEVRAKDQDILDLVGVLHDPETLLRCIAERSFLRHLEGGCSVPVAVHTAIKDGQLYLTG
GVWSLNGAETMQDTMQTTIHVPVQHEDGPEDDPQLVGITARN-IPRQPQLAAENLGISLATLLLNKGAKNILDVARQLNEAH
>MmPD
-----------------MRVIRVGTRKSQLARIQTDTVVAMLKALYPGIQFEIIAMSTTGDKILDTALSKIGEKSLFTKELENALEKNEVDLVV
HSLKDVPTILPPGFTIGAICKRENPCDAVVFHPKFIGKTLETLPEK-SAVGTSSLRRVAQLQRKFPHLEFKSIRGNLNTRLRKLD-ELQEFSAI
VLAVAGLQRMGWQNRVGQILHPEECMYAVGQGALAVEVRAKDQDILDLVSVLHDPETLLRCIAERAFLRHLEGGCSVPVAVHTVMKDGQLYLTG
GVWSLDGSDSMQETMQATIQVPVQQEDGPEDDPQLVGITARN-IPRGAQLAAENLGISLASLLLNKGAKNILDVARQLNDVR
>GgPD
---MAEVRPATGENGVGSRAIRVGTRRSQLARIQTDSVVEMLREFYPDLCFEIVAMSTTGDKILDTALSKIGEKSLFTKELENALERNEVDLVV
HSLKDLPTSLPPGFTIGAICKRENPLDAVVFHPKNCGKTLSVLPEK-SVIGTSSLRRAAQLKKKFPQLEFRDIRGNLNTRLKKLD-EKEDFSAI
ILAAAGLKRMGWENRIGQLLSPEDCLYAVGQGALAVEVRAKDQETLNMVSALQDGETVLCCIAERAFMKRLEGGCSVPVAVNTMLKDGQLYLTG
AVYSLDGSDSLKETMQTSVNYPHRNEDGPNDDVQHVGITAKN-VPGQAQEAAENLGIELASLLLSKGAKHILSVARQLNDAC
>DrPDb
---MSVKTNLQDSDGRVSRVIRVGTRKSQLARIQTDSVVEKLKELYPDVLFEIVAMSTTGDKILDTALSKIGEKSLFTKELENALEKNEVDLVV
HSLKDLPTVLPAGFTIGAVLKRENPHDAVVLHPKNKGKRLDSLSNK-SVIGTSSLRRAAQLKKRFPHLEFKDIRGNLNTRLKKLD-ENNDFSAI
ILAAAGLKRMGWENRISQILGPEDCMYAVGQGALAVEVRARDLDILEMVSVLNDSDTALSCIAERAFLKHLEGGCSVPVAVHSEVKNSQLYLTG
AVYSLDGSDSLKETMQTSFNPDTQAEEKVDEKVQRVGITALK-VAEAAQDAAMKLGVDLGNLLLSKGAKEILTVARQLNDAR
>XtPD
----------MEGAEENKHVIRVGTRKSQLARIQTDSVVEMLSKRFPSTHFDIVAMSTTGDKILDTALSKIGEKSLFTKELENALERNEVDLVV
HSLKDLPTSLPPGFTIGAVCKRENPYDAVVFHPKRYGNTLSTLPEK-SVIGTSSLRRAAQLKKKFPHLEFKDIRGNLNTRMKKLD-EKEDFSAI
ILAAAGLRRMGWKNRIGQILTSDECLYAVGQGALAIEVRAKDQDILSMVSALQDPETVLRCIAERAFMKRLEGGCSVPVAVNTVVKDSQLYLTG
AVYSLDGSDSLKETMQSCINFPQQEVEGPNDDVQHVGITALG-VSHQALESAECLGTGLADLLLSKGAKEILTVARQLNDSR
>XlPD
----------MEGAGETKHVIRVGTRKSQLARIQTDSVVEMLSKRFSSTHFEIVAMSTTGDKILDTALSKIGEKSLFTKELENALEKNEVDLVV
HSLKDLPTSLPPGFTIGAVCKRENPYDAVVFHPKRFGNTLSTLPEK-SVIGTSSLRRAAQLKKKFPHLEFKDIRGNLNTRLRKLD-EQEDFSAI
ILAAAGLRRMGWENRIGQILTSDECLYAVGQGALAVEVRAKDQDILSMVSALQDPETVLRCVAERAFMKKLEGGCSVPVAVSTVVKDSQLYLTG
AVYSLDGSDSLKETMQSCINFPQQEVEGPNDDVQHVGITALG-VSHQALESAECLGTGLADLLLSKGAKEILTVARQLNDSR
>CiPD
--------------MGDNNILRVGTRKSELAMVQTNWVVNQLKSLNKSLEVEIVQMATIGDKILDKSLSKTGEKSLFTKELEDALIANQVDLVV
NSLKDMPSTLPPNLTISAVSRREDPRDAVIMHPDNVGKRLEDLPPG-SIVGTSSLRRVAQLKKLFPDLKFKSIRGNLNTRLRKLD-DASNFSAI
ILAVAGVKRMGWNARISYTLTTDECLYAIGQGALGIETRADDMKSIKLVSQLHDKDTVIRTVAERAFLKTLGGGCSVPQAVHSWIIEDELYMRG
AVFNLDGSKSLYETMQTSLFVNEDDKMEPDSCCEFASITTHKFINPMLLKASGHLGRDLALLLLQRGATAILDEAREQNLLR
```

>SpPD
-------------MADKMEIVRVGSRKSELAMIQTNHVVEMLQKVHPETEFKIVTMETIGDHIQDKPLASIGESNLFTKELEKALALDEVDMLV
HSLKDMPSRLPSNMAIGAICEREDPHDALVLHKKLTGISIETMKEG-SVLGTSSVRRVAQLKRKFPHLVFKDVRGNLNTRLRKLD-EGQVYDGL
VLAVAGMMRMGWEHRISQILKPDICKYSVSQGALGIEIKTKNRRMHELLRQLNHVPTLIRCIAERGLLKTLEGGCSAPVAVHSELEENR-----
--HSLDAETN-------------------EEMKLFSSVIA-PWVSQHAMQEAEALGVSVAKGLFDKGAGPILEAAKEEIAER
>DmPD
-------------MSAQEKVIRVGSRKSELALIQTKHVIGRLQKLYPKQKFEIHTMSTFGDRVLNVSLPKIGEKSLFTRDLEDALRNGGVDFVV
HSLKDLPTALPTGMAIGAVLEREDARDALVLRENFKGHTIASLPKG-SVIGTSSLRRTAQIRRMYPHLTVCDIRGNLNTRLAKLDAADSKFSGI
ILAQAGLVRMGWMSRISQVLEPTDLLYAVGQGALAVECRANDDQVLAMLQKLMCLNTTCRILAERSFLKTLGGGCSAPVAVWSNLKGEPLSLTG
AVWSLDGAIEIRNHLACALNEQKLEGEAPDVENLFCGLYQHACHSRGIYEKANQLGKTLAEDLIKRGALDVMKVAQAEIHGK
>MmuPD
MSGNGNAAATAEENSPKMRVIRVGTRKSQLARIQTDSVVATLKALYPGLQFEIIAMSTTGDKILDTALSKIGEKSLFTKELEHALEKNEVDLVV
HSLKDLPTVLPPGFTIGAICKRENPHDAVVFHPKFVGKTLETLPEK-SVVGTSSLRRAAQLQRKFSHLEFRSIRGNLNTRLRKLD-EQQEFSAI
ILATAGLQRMGWHNRVGQILHPEECMYAVGQGALGVEVRAKDQDILDLVGVLHDPETLLRCIAERAFLRHLEGGCSVPVAVHTAMKDGQLYLTG
GVWSLDGSDSIQDTMQATIHVPAQHEDGPEDDPQLVGITARN-IPRGPQLAAQNLGISLANLLLSKGAKNILDVARQLNDAH
>MgPD
----------------------MGRASLQLARIQTDSVVEMLREFYPDLCFEIVAMSTTGDKILDTALSKIGEKSLFTKELENALERNEVDLVV
HSLKDLPTSLPPGFTIGAICKRENPLDAVVFHPKNCGKTLSLLPEK-SVIGTSSLRRAAQLKKKFPQLEFRDIRGNLNTRLKKLD-EKEDFSAI
ILAAAGLKRMGWENRIGQLLSPEDCLYAVGQGALAVEVRAKDQETLNMVSVLHDGETVLCCIAERAFMKHLEGGCSVPVAVNTMLKDGQLYLTG
AVYSLDGSDSLKETMQASVNYPQRNEDGPNDDVQHVGITAKN-VSGQAQEAAENLGIELASLLLSKGAKHILRVARQLNDAC
>TgPD
--MLRELYPDLHFEIVAMSTTGDKILDTALSKIGEKSLFTKELENALERNEVDLVV
HSLKDLPTSLPPDFTIGAVCKRENPLDAVVFHPKNCGKTLSLLPEK-SVIGTSSLRRAAQLKKKFPHLEFRDIRGNLNTRLKKLD-EKEDFSAI
ILAAAGLKRMGWENRIGQLLSPEDCLYAVGQGALAVEVRAKDQEILNMVSALHDADTVLCCIAERAFMKRLEGGCSVPVAVNTLLKDGQLYLTG
AVYSLDGSDSLKETMQTGVSYPHQNEDGPNDDVQHVGITAKN-VPGQAQEAAENLGVELASLLLSKGAKHILSVARQLNDAR
>AlPD
--MLQKHHPQLHFEIIGMTTTGDKILDTALSKIGEKSLFTKELENALERNEVDLVV
HSLKDLPTSLPPGFTIGAVCKRENPHDAVVFHPKHVGKTLSSLPDK-SVIGTSSLRRAAQLKRAFPQLQFKDIRGNLNTRLKKLD-EKDDFSAI
VLATAGLRRMGWEDRIGQILTPMECLYAVGQGALAVEVRARDQEILEMVSVLHDGETVLRCIAERAFMKHLEGGCSVPVAVSTTLKDSQLYLTG
AVYSLDGSESLCETMQTNVKFPMEDEEGPDDNIQHLGIMART-EPRAAQAAAEKLGLDLAALLLSQGARPILDAARQLNAA-
>OlPD
---MSTIGDKILDTALSKIGEKSLFTKELENALERNEVDLVV
HSLKDLPTSLPAGFTIGAVLRRENPHDAVVLHPKNAGKTLASLPKN-SVVGTSSLRRAAQLKRRFPHLEFKDIRGNLNTRLKKLD-DKEDFAAI
ILAAAGLKRMGWEDRISAVLQPEDCMYAVGQGALAVEVRAKDTDILDMVSVLHDPDTVLRCIAERAFLKRLEGGCSVPVAVHTYIKDSQLYLTG
AVYSLDGSESLKETMQTSICSGGSQCEEVDERLQRVGVTATK-IPADLQDGAERLGVDLADLLLTKGAKEILTVARQLNDAR
>TrPD
---MSTTGDKILDTALSKIGEKSLFTKELENALEKNEVDLVV
HSLKDLPTTLPPGFTIGAVLKRENPHDAVVLHPKNAGKSLDALPAN-SVIGTSSLRRAAQLKKRFPHLDFKDIRGNLNTRLKKLD-EKEDFAAI
ILAAAGLRRMGWENRISQILEPDDCMYAVGQGALAVEVRARDADILEMVSVLHDPDTVLRCIAERAFLKHLEGGCSVPVAVHTVVKDSQLYLTG
AVYSLDGSDSLKETMQTGIAAAADG--SPDECVQRVGVTARK-IPGEAQDKAERLGVDLAKLLLSKGAKEILTVARQLNDAR
>GaPD
---MTTIGDNILDRALSKIGEKSLFTKELENALEKNEVDLVV
HSLKDLPTTLPSGFTIGAVLKRENPHDAVVLHPKHLGRTLETLPES-SVIGTSSLRRAAQLKKRFPQLLFKDIRGNLNTRLKKLD-EKDDYAAI
VLAAAGLRRMGWDDRIGQILRPEDCMYAVGQGALAVEVRARDADILEMVSVLHDPETVLRCIAERAFLRRLEGGCSVPVAVHTEVKNSQLYLTG
AVFSLDGSDSLKDTMQTSVAADDKQKDAVDERVQRVGVTASG-VPGGAQDGAERLGEDLANLLLSKGAKEILTVARQLNDAR
>TnPD2
MTGQTGSKSLQDGNGKVHRVIRIGTRKSQLARIQTDSVAEKLKELYPDVHLEIVAMSTTGDKILDTALSKIGEKSLFTKELENALERNEVDLVV
HSLKDLPTSLPPGFTIGAVLKRENPHDAVVLHPKNAGKTLDALPAH-SVIGTSSLRRAAQLKKRFPHLDFRSQRGNLNTRLKKLD-EKEDFAAI
ILAAAGLRRMGWDNRISQILEPEDCMYAVGQGALAVEVRAKDADILEMVSVLHDPDTVLRCIAERAFLKHLEGGCSVPVAVHTKVKDSQLYLTG
AVYSLDGSDSLKETMQTSVAAPADG-QSPVDECARVGVTANK-IPGEAQDRAERLGVDLAKLLLSKGAKEILTVARQLNDAR
>AmePD
-----------MTSTDTRDVIRVGSRKSELALKQTKYVIECLKEYHPTKEFQIITMSTKGDKILDKSLPKIGEKSLFTEELELALESGRVDFVV
HSLKDLPTSLPEGMALGAILKREDPRDAVVMSKKYKNKTLSTLPEG-SVIGTSSLRRSAQLARNMPHLKVENIRGNLNTRLRKLDDENGPFAAI
ILAAAGLKRMNWENRISQLLEPEEALYAVGQGALGVECRETDWKILSLLEPLYDVETTLRCVCERSFLKTLGGGCSAPVAVSSTLKNKILTVTG
AVWSLDGQKFIKDTSKSKLYIPDDDGEPPREPQLYCSVAPGK-VSNISLLGAEQLGKDLGKNLIEKNALDVMAEARNEILNS
>LaPD
MSGNGDADATTEENGPKMRVIRVGTRKSQLARIQTDSVVTTLKTLYPGLQFEIIAMSTTGDKILDTALSKIGEKSLFTKELEHALEKNEVDLVV

HSLKDLPTVLPPGFTIGAICRRESPYDAVVFHPKFVGKTLETLPERSSVVGTSSLRRAAQLQRKFPHLEFRSIRGNLNTRLRKLD-EQQVFSAI
ILAEAGLHRMGWQNRVGQILHPEECMYAVGQGALGIEIRAKDQHILDLVSVLHDPETLLRCIAERAFLRHLEGGCSVPVAVHTAVKDGQLYLTG
GVWSLDGSDSMQETMQATVHIPAQHEDGPEDDPQLVGITAQN-VPRGPQLAAENLGISLASLLLNKGAKNILDVARQLNDAH
>OcPD
-----------------MRVIRVGTRKSQLARIQTDSVVATLKTLYPGLRFEIIAMSTTGDKILDTALSKIGEKSLFTKELEHALEKNEVDLVV
HSLKDLPTVLPPGFTVGAICKRENPCDAVVFHPKFVGKTLATLPER-SVVGTSSLRRAAQLQRKFPHLEFKSIRGNLNTRLRKLD-EQQEFSAI
ILAVAGLQRMGWHNRVGQILHPEECMYAVGQGALGVEVRAKDQDILDLVGVLHDPETLLRCIAERAFLKHLEGGCSVPVAVHTAMKDGQLYMTG
GVWSLDGSDTMQETMQASIQVPAQHEDGPEDDPQLVGITARN-IPRGPQLAAENLGISLANLLLNKGAKNILDVARQLNDAH
>HmPD
TEDNGSSKKKYSLVTEKTSVV-VGSRKSQLAVIQTKSIVAALCQKEKDVECIIETMDTLGDKILHIALPKIGEKSLFTKDLELALSEKRVDFLV
HSLKDLPTTLPQGMAISTIYKRDDPRDCIIFHPKHNGKKLADLPEN-SIIGTSSLRRVAQLKRKYNKLTFQSVRGNLNTRLQKLE-EESLYDAI
VLAKAGVDRMGWTEKIGQILSEDECLYAIGQGAITVEVRNDDKYTISILAQLTDMETLLACVAERCFMRTLNGGCSTPIACHSQFSGNNYSLRG
IVLNADGSRFLDSTVSTTLQVNILTDEPPSYTVSKSGVVINS-LYINEFIKAEKCGEELAYCLKKLGADDVLKEVRSKLPNI
>DrPDa
--MEGPFKYIREGNGKASRVIRMGTRKSQLARIQTDSVAGTLKQLYPDVRLEIVAMSTIGDKILDTALSKIGEKSLFTKELENALERNEVDIVV
HSLKDLPTSLPPGFTIGAILERENPHDAVVLHPKNAGLTLDSLPEK-SVIGTSSLRRAAQLKKRFPQLEFENIRGNLNTRLKKLD-EKDDYAAI
ILAAAGLKRMGWESRISQVLGPEDCMYAVGQGALAVEVRAQDKDILEMVSVLHHPDTVLRCISERAFLKQLEGGCSVPVAVHTEVKGSMLYLTG
AVYSLDGADCLKDTMQTCVELDNKVNEST-QRSANVGVTACN-ISSSALEAAEKLGLDLANVLLNKGAKDILTTARKLNDAR
>DaPD
-------------MSAQEKVIRVGSRKSELALIQTKHVIGRLQKLYPKQKFEIHTMSTFGDRVLNVSLPKIGEKSLFTRDLEDALRNGGVDFVV
HSLKDLPTALPTGMAIGAVLEREDARDALVLRENFKGHTIASLPNG-SVIGTSSLRRTAQIRRQYPHLVVCDIRGNLNTRLAKLDAADSKFAGI
ILAQAGLVRMGWMSRISQVLEPTDLLYAVGQGALAVECRANDEPVLAMLQKLMCLNTTCRILAERSFLKTLGGGCSAPVAVWSNLKGEPLSLTG
AVWSLDGAIEIRNHLACALNEEKSETEAPDVENLFCGLYQHACYSRGIYEKANELGKTLAEELIKRGALDVMKVAQAEIHGK
>DpPD
-------------MSAQEKVIRVGSRKSELALIQTKHVIGPTPELYLKQRFEIHTMSTFGDRVLNVSLPKIGEKSLFTRDLEDALRNGGVDFVV
HSLKDLPTALPTGMAIGAVLEREDARDALVLRENFQGHTIASLPQG-SVIGTSSLRRTAQIRRLYPHLIVCDIRGNLNTRLAKLDAADSRFAGI
ILAQAGLVRMGWMSRISQVLEPTDLLYAVGQGALAVECRANDVQVLAMLQKLMCLNTTCRILAERSFLKTLGGGCSAPVAVWSNLQGEPLSLTG
AVWSLDGAIEIRNHLSCALSEKPTG-EAPDVDNLFCGLYQHPCYSRQVYERANLLGKNLAEELIKKGALDVMKIAQAEIHGK
>AaPD
-----------MTGASKEHVIRVGSRKSELALIQTKHVIACLQKLNPDVQYEIHTMTTVGDRVLNKSLPKIGEKSLFTKDLEDALRNGGVDFVV
HSLKDLPTSLPIGMAIGAVLEREDPRDALVLNEKFRGKTLSTLPKG-SVIGTSSLRRSAQLARLHPHLVVCDIRGNLNTRLAKLDAEGSKFAGI
VLAQAGLVRMGWEKRIDQVIEPSEILYAVGQGALAVECRSNDEYILNMLSKLCHLETQCKILVERSFLKTLGGGCSAPVAVCTSLKRKHLNIQG
AVWSLDGKTEIRADDGCSVELEHVQQDHPNSEHLFCGMHRNKNVPNESFEACEKLGQQLAHFLISKGALDVMKCAQNEIHSK
>CqPD
NENLEREIVAPKLESTSEHLFQTGIRHSKLALIQTKYVISCLQKLNPDVVYEIRTMTTVGDRVLNKSLPKIGEKSLFTKDLEDALRTGGVDFVV
HSLKDLPTALPLGMAIGAVLERDDPRDALVLNEAHRGRTLSTLPKG-SVVGTSSLRRSAQLARKYENVVVCDIRGNLNTRLAKLDADGSKFAGI
ILAQAGLVRMGWNARVSQVIEPGEILYAVGQGALAVECRSNDEYILDMLSKLCHLETQCKILK--------------------------MYRDE
SVLNDQLLMALLGQFQY---------EHP--NKEHLG-------PPEPPSHLERFSPEDLHKIYQPKADAITSL--------
>NvPD
-------------------------------MIQTNFIVCKLKKLQPELSVELVSMKTIGDEVLDKALPKIGETNLFTKELELALAAGKVDFLV
HSLKDLPSLLPEGMGLAAIYCRDDPRDAVIFHAKHKGASLASLPEG-SNIGTSSLRRVAQLKRNFPHLKFESIRGNLNTRLRKLD-EGDKYDAI
VLAKAGLDRMGWEERTDQVLTPKECLYAVGQGALAVELNVSDLKTFELISQLFDFNTTVRCATERSFLRTL-----------------------
--VRT-----

#UROS#
>HsUS
MKVLLLKDAKEDDCGQDPYIRELGLYGLEATLIPVLSFEFLSLPSFSEKLSHPEDYGGLIFTSPRAVEAAELCLEQNNKTEVWERSLKEKWNAK
SVYVVGNATASLVSKIGLDTEGETCGNAEKLAEYICSRESSAL-PLLFPCGNLKREILPKALKDKGIAMESITVYQTVAHPGIQGNLNSYYSQQ
GVPASITFFSPSGLTYSLKHIQELSGDNIDQIKFAAIGPTTARALAAQGLPVSCTAESPTPQALATGIRKALQPHGCC
>MmUS
MKVLLLKDAKEDDSGLDPYIQELRLCGLEATLIPVLSFEFMSLPSLSEKLSHPEGFGGLIFTSPRAVEAVKLCLEKDNKTEAWEKSLKDRWNAK
SVYVVGSATASLVNKIGLDAEGAGSGNAEKLAEYIC--SKPSSEPLLFPCGTIKGDTLPKMLKDKGIPMESMHVYQTVPHPGIQGSLKSYYEDQ
GIPASITFFSPSGLKYSLEYIQALSGSSFDQIKFIAIGPSTTRAMAAKGLPVSCTAESPTPQALAAGIRNVLKPNHCC
>DrUS
MKVLLLKEPRESESEADPYIKELASCGHTATLIPALSFRFVSLNELSERLFQPERFGGLIFTSPRAVEAVKTSLESQKLRGKWDAVK-DKWNTK
SVYVVGKATASLVVDLGLRPLGEDSGTADALALLILQRENPDIEPLFFPCGSIKREVLPTALRNSHIPLETLTVYQTSEHPDLQKNISDYFTQQ
GVPACVAFFSPSGVSFCLDLLKDLSGCQLEQIKFASIGRTTADALQARGLTVSCCAEKPTAKHLAIAITQALRDL---

>GgUS
MKVLLLKDPKDKESGPDPYVKELGLYGFEATLIPVLSFEFVSLEGLFEKLSHPECYGGLVFTSPRALEAIKICLRENSKNEAWSKSLKQEWNAK
PTYVVGKATASLVEEIGLCPQGEKSGNAEKLAEYICSREGPHSSPLLFPCGALKREVLPTALKEKGIPLESLTVYQTAQHEHLQQSLSSYFSQQ
GVPASIVFFSPSGVKFCLQHIQKLSGDFANRIKFAAIGPTTAEAMEAAGIPVSCTAESPTPQDLTAGIQKALRPQNCI
>XtUS
MKVLLLKDPKTKDLASDPYVKELSSYGLQATLIPVLSFKFVSLDHFFDKLSHPESYAGLIFTSPRAVEAVKLCLQTPDHKEAWNDRLCAKWNSK
PVYVVGKATASLVEEIGLSSEGEGSGNAEKLAECICSKGLSYSAPILFPCGSLKREVLPKKLQEKKVPLETITVYQTAPHPAVQDSLMNYFTKE
GVPASVVFFSPSGVKYCLKFLKDLPNDQLNQIKFGAIGPTTAEAMAEEGIPVSCTALNPTPQDLAIGIKQADMQ----
>BtUS
MKVLLLKDPKEDDCGQDPYVRELGLYGLEATLIPVLSFEFLSLPSLSEKLSHPEGYGGLIFTSPRAVEAVERCLQKDTKAEVWKKSLKEKWNAK
SVYVVGNATASLVNRIGLHTEGETCGNAEKLAEYICSREPSAL-PLLFPCGTLKREILPKMLKDKGIPLESVTVYQTIPHPGIQGNLTSYYTQQ
GVPASITFFSPSGLTHSLKHIQELSGDSIGQIKFVAIGPTTAQALAAQGLPVSCTAESPTPRALAAGIRTTLQPHAC-
>ClfUS
MKVLLLKDPKEDDCGQDPYIRELGLYGFEATLIPVLSFEFLSLPSFSEKLSHPEGYGGLIFTSPRAVEAVELCLEKDNKTEVWKHSLKEKWNAK
TVYVVGNATASLVNKIGLDAEGANCGNAEKLAEYICSRESPAL-PLLFPCGTVKGEILPQMLKDRGIPMESITVYQKVPHPGIQVNLNSYYAKQ
GIPASITFFSPSGLAYSLKHIQELSGDNIDRIKFAAIGPSTARALAAQGLPVSCTAESPTPQGLATGLRLALEAPRC-
>XlUS
MKVLLLKDPKTKDLASDPYVKELSSHGLQATLIPVLSFKFVSLDHFFDKLSHPESYAGLIFTSPRAVEAVKLCLQKPAHKEAWKDHLCAKWNSK
PVYVVGKATASLVEEIGLSSEGEGSGNAEKLAECICSKGLSYSAPILFPCGSLKKEVLPKKLQEKNVPLETITVYQTGPHPAIQVSLSDYFTKE
GVPASIVFFSPSGLKYCLLFLKDLPSDQLNQIKFAAIGPTTAEAMAEEGIPVSCTAQNPTPQDLAIGIKQIGMQ----
>DmUS
RTVIIFKSESES---SDVYAETLEKHDFNPVFVPTLSFGFKNLDELRAKLQNPDKYAGIIFTSPRCVEAVAESLNLGELPGGWKMLH-------
-NYAVGEVTHNLLSTLDLFTHGKQTGNARALGDYIVDTFDGSRAPLLLPCGNLATDTLLSKLAENGFSVDACEVYETRCHPELGANVERALEIY
GSIEFLAFFSPSGVN-CAQQYFTSRQLSMDKWKLVAIGPSTRRALESLGQKVYCTAERPTVEHLVKVLLNPQDSRERL
>BfUS
MGILLLREPREEK-GTDPYEQEFVAAGLQVKSVEVLSFEFCSLPELRSLVKKPEEFSGMVFTSQRSVEAVTRCLQE------------------
------------ARSLGLTPSGEECGTATQLADLITQKFTSNSKALLFPCGFMRRETLPSALSAANIPLTELRVYQTTAHPDIDKRLQAYIAEQ
----------------------------------IAAIGPTTAEAMTSAGLSVACTAEKPNPQALLAAIQATIAAQATE
>SpUS
MNSLLLRSAQEND-TKDKYQEAFQEADVDCVSLSPIGFEFIDLDTLYAHISHPEDHSGLIFSSPRTVQSVALCLERHDTNDEWNSRLRDEWSKL
HAFSVGTSTGGVVRKLGLHPMGEDSGNAENLCKIILEAVKPGSKPLLYPCGTMRRETIPKTLQKEGILFKEDVAYQTVPNPDLEQELAKYLQEK
GAPFCIVFFSPSGVQYSKE-VFDKFKADLHSTKLLAIGGTTRAAMQEHGYVVAGVAEKPNPTALLQAIQQCQGGTDG-
>MmuUS
MKVLLLKDAKEDDCGQDPYIRELGLYGLEATLIPVLSFEFLSLPSFSEKLSHPEDYGGLIFTSPRAVEAAQLCLEKNNKTEVWERSLKEKWNAK
SVYVVGNATASLVSKIGLDTEGETCGNAEKLAEYICSRESSAL-PLLFPCGNLKREILPKALKDKGIAMESVTVYQTIPHPGIQGNLNSYYSQQ
GVPASITFFSPSGLTYSLKHIQELSGDNIDQIKFAAIGPTTARALAAQGLPVSCTAESPTPQALATGIRKALQPHGCC
>MgUS
MKVLLLKDPKDKESGPDPYVKELGLCGFEATLIPVLSFEFVSLEDLFEKLSHPERYGGLVFTSPRALEAIKICLREKSKNEAWSKSLKQQWNTK
PTYVVGKATASLVEEIGLSPQGEKSGNAEKLAEYICSREKPHSSSLLFPCGALKREVLPTALKEKGIPLESLTVYQTAQHEHLQQSLSSYFSQQ
GIPASIVFFSPSGVKFCLQHIQKLSGDSANQIKFAAIGPTTAEAMEAAGIPVSCTAESPTPQDLAAGIQKALRPQNCI
>TgUS
MKVLLLKDPKDKDSGPDPYIKELGSYSLEATLIPVLSFDFTSLESLFGKLSHPECYGGLVFTSPRALEAIKICLKEKSKKEAWSNSLKQMWNIK
PTYVVGKATASLVEEIGLVPQGEKAGNAEKLAEYICSREKPNSSALLFPCGALKREVLPTVLREKGIPLESLTVYQTAQHPDLQESLSSYFSQQ
GIPASVVFFSPSGVKFCLQHIQKLSGDLINQIKFAAIGPSTAEALRGAGVSVSCTAGSPTAQDLAAAIHTALQPHPC-
>AlUS
MKVLLLKDPKESENGPDPYIEELGSHGLEATLIPVLAFEFISLQSLFEKLSCPEQYLGLIFTSPRAVEAIKLCLADKSKKEDWKNYLREKWNTK
SIYVVGKATAVLVAELGLIPQGENSGNAEKLSGYICSRESPNSLPLLFPCGSLKRETLPTILREKGIALEILTVYQTTQHPDLQESLRNYFSQQ
GVPASITFFSPSGVKYCLKQIMKLSGDLITQIKFAAIGPTTAEALETEGISVSCTATSPTPQDLTTGIRRSLQLKDC-
>OlUS
MHVLLLKEPREEASGPDPYLKELASHGHKATLIPVLSFKFVSLNTLADKLFQPEKHGGLIFTSPRAVEAVKMCLDAEKRKEEWNKSLRDQWNTK
PIYVVGKATAALVQNLGLTPLGEDTGTAEVLSRVIIEREDTNIPPFLFPCGSIKREVLPTALREKGVPLETLTVYQTSEHPDLERNLNNFFTEQ
GTPACVAFFSPSGVKFCLELILRLSGDQRTQIKFAAIGPTTRDAMAAEGLCVSCTAEKPTAEHLAAEISKALIKKYCC
>TrUS
MHVLLLKEPRDGESGIDPYIKELASHGHTATLIPVLSFAFVSLSTLSDKLFQPEKHGGLIFTSPRAVEAVKMCLEAEKRVEEWNKCVRDQWNAK
SVYVVGKATAALVRSLGLTPLGEDTGTAEVLSRVIIEREDTNIPPLLFPCGSIKREVLPTALRGSGVPLEMLTVYQTAEHPNLEENLRSYFTEQ
GTPASIAFFSPSGVKFCLETVRRLSGDQLPQIKFAAIGPTTEEAMAAEGLCVSCAAEKPTAGHLAAAIAKALQ-----
>GaUS

MHVLLLKEPRDGESGPDPYIKELATHGHKATLIPVLSFKFVSLNTLSDKLFQPEKHGGLVFTSPRAVEAVQMCLEAEERREEWNSSVKEKWNSK
SIYVVGKATAALVGSLGLKALGEDTGTADVLSRLIIEREDTNIPPLFFPCGSIKREVLPTALKGNGVPLETLTVYQTAEHPDLEKNLKNYFTEQ
GAPASVAFFSPSGVKFCLEAVRRLSGDRLTQIEFAAIGPTTRDAMTAEGLRVSCTAEKPTAEHLAAAIAKALQQP---
>CiUS
MKIVLMRASTGC---NDEYVNTLNKIG-DVTSIPVLSFNFCNCDLIKDAISAPDSWSGIIFTSKRSVEAVQLSLDT-PLSELWASKP-------
-CYAVGESTAKMAENLGFISKGGNSGNAQVLAELIVEQKLVNNQPLLFICGQLKRETLPSVLTCNEIPFHTITAYETVKNPVVDEAVQSYVEVN
GSPDFITFFSPSGHRFCFNTWQSALKGDFSATKIVAIGETTAHSFASQPDISVVTAERPNPESLFKIIKANCESL---
>CqUS
KKVVVLKSESDN---SDTYSSLLQKHGFEPVLVPTLDFSFKNLDVLRDRLLSPYKYSGLIFTSPRSITAVRDALGGQRLKDDWRTLE-------
-NYSVGETSHDLCSHLQLNTKGQQSGNASNLADFIKDLYNKTITPFLFPCGNLKQDVLQYKLSEYGYSLDSVEVYETVPHKDLERNLVQLFR--
GAPAFLLFFSPSGINYCSA-IFERNKLDLGGCRIVAIGPSTKKAIENKGLTVYRTAEKPSPDLTLNLYCVYMFYITSP
>AaUS
KKVVILKSESDN---SEAYPALLRKHGFEPIFVPTLDFSF-RLDILRDRLLSPYS---IRYHSPRAITAVRDALDGNRLKDDWKPLE-------
-NYSVGETSSELQRTLELDTKGQHSGNASNLADFIKDLYNKTVTPFLFPCGNLKQDVLQNKLSEYGYSLDCVEVYETVPHRDLERNLVELFREG
AQPGYLLFFSPSGMNYCAS-VFERHKLDINGCRIVAIGPSTKKAIENKGYAVHRTAEKPSPEYVVSALLDT-------
>AdUS
TVVVLFRSPTPKP-KEDEYHKVLRENGMKPFSIPVLSFQFDNQQQLFQKLREPFHFRGMVLTSQRAVEAMERCVNELISHEVWKNTLRNDWQDK
AIFVVGEATAKALDKLGLESTGHEAGSAEALVPVILHSVRQGHDPLLFPCGNLRRETIPTEMEKAGISLDSVQVYRTCADLNIEQSLKDVIKEK
V-----------------------------------LI--------------LLLHCI------------------------
>LaUS
MKVLLLKDPKEDDCGQDPYIRELGLYGLEATLIPVLSFEFLSLPSFSEKLSHPEGYGGLIFTSPRAVEAVELCLEKDSKTEAWEKSLKEKWNAK
PVYVVGSATASLVRKIGLDTEGEKCGNAEKLAEYICSRESPAL-PLLFPCGTLKGEILPKMLKDQGIPMESITVYQKIPHPGIQGNLKSYFSKQ
GVPASITFFSPSGLAYGLQHIQELSGDHINQIKFAAIGPTTARALVAQGLPVSCTAESPTPGALAAGIRKALQAHSCC
>OcUS
MKVLLLKDAKDDACGQDPYVRELGLHGLEATLIPVLAFEFLSLPSFSEKLCRPEDYGGLIFTSPRAVEAAQLCLEKSSKAEVWERSLREKWNVK
SVYVVGGATASRVSRIGLRADGEHSGTAEKLAEYICSKESSPL-PLLFPCGTLKREVLPTVLKEQGVPMESITVYETVPHPGIRGNLDSYYSKQ
GVPDSVAFFSPSGVKYSLKHIEELSGDSVGQIKFAAIGPTTARALAAQGLRVSCTAESPTPQALAAGLRKALPLPGGC
>HmUS
MSIVLLREEDDD----DYYHKLLYANGFFVCSIPVLCFSYYNIEKLEEFFHNSSEYSALVITSKHATYALEVVLKKNNLSFSLPQNF-------
YIYVVGKSTELYLQKLGLSSLGSDSGNADQLADYIIKRKIFHLKPLMFVCGNLARETIPDKLRNFGLTVETISCYKTIANDNFPEKISQFIKQY
DIPNVIVFFSPSGVKFYFSEICAKSYWEF--IKMTVINTIASSGFTNE-LSLLCSSTKLERDEVLGKLCENVDTSFYK
>NvUS
PSVLLLRSSSDDP-KQDRYHMELRSHGFQCTSIPVLSFEFENLDKLEKMLSEPTEYGGIILTSARGVEAMEKCIEE------------------
------KISLSAKKKLGLESVGHQAGSAEMLAPMILEGAHPQTKPFLFPCGNLRKETIPTEMQNAGVPLKGLQVYKTCKHTELAQNLTSHVEEK
GVPSVIVFFSPSGVQFVLSLLTEIFG-DFSALKLVAIGATTSAAMIKHGLSVAAVAKQPSPKSLAECISQCF------
>DaUS
RTVIIFKSGSET---DDVYTETLEKHDFQPVFVPTLSFGFKNLPELRQKLQTPDKYAGIIFTSPRCVEAVAESLNLGQLPGGWKLLH-------
-NYAVGEVTHNLLSSLDLFTHGKQTGNARALGDYIVETFDGSRDPLLIPCGNLATDTLLSKLAENGFALDACEVYNTLCHPELAENVERAIATY
GSIEFLAFFSPSGVN-CAQEYFRSRNLSMDKWKLVAIGPSTRRALESQGLKVYCTAERPTVEHLVKVLLNPQDSRERL
>DpUS
RTVIIFKSESDS---TDVYAETLLGHNFNPVFVPTLCFGFKNLQELGNKLQTPDKYAGIIF---HQSTAVNEALLLGELPGGWKLLH-------
-NYAVGEVTHNLLSTLQLFTHGKQTGNARNLGEYIVDTFDGSRDPLLLPCGNLATDTLLSKLAENGFCVDACEVYETKCSPQLGECVERALKEA
GSVEFLAFFSPSGVA-CAMEYFRAHNISLEPWKLVAIGPSTRRALESQGQKVYCTAERPTVEHLVKVLLNPQDSRERL

#UROD#
>HsUD
GMEVTMVPGKGPSFPEPLREEQDLERLRDPEVVASELGYVFQAITLTRQRLAGRVPLIGFAGAPWTLMTYMVEGGGSSTMAQAKRWLYQRPQAS
HQLLRILTDALVPYLVGQVVAGAQALQLFESHAGHLGPQLFNKFALPYIRDVAKQVKARLREAGLAPVPMIIFAKDGHFALEELAQAGYEVVGL
DWTVAPKKARECVGKTVTLQGNLDPCALYASEEEIGQLVKQMLDDFGPHRYIANLGHGLYPDMDPEHVGAFVDAVHKHSR
>MmUD
GMEVTMVPGKGPSFPEPLREERDLERLRDPAAAASELGYVFQAITLTRQRLAGRVPLIGFAGAPWTLMTYMVEGGSSSTMAQAKRWLYQRPQAS
HKLLGILTDVLVPYLIGQVAAGAQALQLFESHAGHLGTELFSKFALPYIRDVAKRVKAGLQKAGLAPVPMIIFAKDGHFALEELAQAGYEVVGL
DWTVAPKKARERVGKAVTLQGNLDPCALYASEEEIGRLVQQMLDDFGPQRYIANLGHGLYPDMDPERVGAFVDAVHKHSR
>DrUD
GMEVQMCPGKGPTFPEPLKEPEDLQRLKTQVDVYSELDYVFKAITLTRHKIEGKVPLIGFTGAPWTLMSYMIEGGGSATHSKAKRWLYRYPEAS
HKLLSQLTDVIVEYLLGQVKAGAQALQVFESHTGCLGPVEFKEFSLPYLRDIARRVKDKIKESGLDNVPMIVFAKDGHYGLEDLSESAYEVVGL
DWTIDPRSARVRTGGKVSLQGNMDPCALYGTKESISEIVRRMLEGFGTKGYIANLGHGLYPDMDPENVGAFVEAVHNHSR

>GgUD
GMEVVMVPGKGPTFPEPLKEVEDLLKLRQKVDVTAELGYVFQAITLTRHSLEGKVPLIGFSGAPWTLMSYMVEGGGSTTMAKAKSWLYRHPEAS
HRLLRLLTDVVTDYLVGQVAAGAQALQLFESHAGHLGPEQFQEFALPYIRDIARDVKSKLKAEALSLVPMIVFAKDAHYALRDLAQAGYEVVGL
DWTIQPQEARAQVGKGVTLQGNLDPCALYAPKEKIGELVKKMLENFGTQRYIANLGHGLYPDMNPEHVGAFVEAVHAHSR
>XtUD
GMEIRMEPGRGPVFPEPLQTPQDLHKLRSSVDVSEELGYVFHAITLTRHKIEGKVPLIGFTGAPWTLMTYMIEGGGSNTMSKAKRWLYQHPSAS
HQLLKQLTDVIVEYLVGQVAAGAQALQVFESHAGCLGPQQFSDFSLPYLRDISRRVKHKLKAEGLDQVPMIVFAKDAHYALEDLSQSGYEVVGL
DWTIRPQEARERTGKRVTLQGNMDPCALYASKEDIEKTVKKMIEGFGQRGYIANLGHGLYPDMDPDHVGAFISAVHKYSQ
>BtUD
GMEVTMVPGKGPSFPEPLREERDLERLQDPATVASELGYVFQAITLTRQRLAGRVPLIGFAGAPWTLMTYMVEGGGSSTMSQAKRWLYQRPQAS
HQLLRILTDALVPYLVGQVAAGAQALQLFESHAGHLGPQLFSKFALPYIRDVSKRVKAGLQEAGLAPVPMIIFAKDGHFALEELAQAGYEVVGL
DWTVAPEKARERVGKTVTLQGNLDPCALYASEEEIGKLVQQMLNDFGPQRYIANLGHGLYPDMDPEHVGAFVDAVHKHSR
>CifUD
GMEVTMVPGKGPSFPEPLREERDLERLRDPAAVASELGYVFQAITLTRQRLAGRVPLIGFAGAPWTLMTYMIEGGSSSTMAQAKRWLYQKPQAS
HQLLRILTDALVPYLVGQVAAGAQALQLFESHAGHLGPQLFNKFALPYVRDVAKRVKARVQEAGLAPVPMIIFAKDGHFALEELAQAGYEVVGL
DWTVAPEKARERVGKMVTLQGNLDPCALYASEEEIGRLVQKMLDDFGPQRYIANLGHGLYPDMDPEHVGAFVDAVHKYSR
>XlUD
GMEIRMEPGRGPVFPEPLQTPQDLQKLRSSVDVSEELGYVFRAITLTRHKLEGKVPLIGFTGAPWTLMTYMIEGGGSNTMSKAKRWLYQHPSAS
HQLLKQLTDVIVEYLVGQVAAGAQALQVFESHAGCLGPQQFSDFSLPYLKDISRRVKHKLKAEGLNQVPMIVFAKDAHYALEDLSQSDYEVVGL
DWTIRPQEARERTGKRVTLQGNMDPCALYASKGDIEKTVKKMIEGFGRRGYIANLGHGLYPDMDPDHVGAFISAVHKYSQ
>BfUD
GMTVEMIPGKGPSFPEPLKLPEDLSKLDKDVDVNKTLGYVFDAITLTRHKLEGKVPLIGFSGAPWTLMCYMIEGGGSESMSKPKQWLYKYEEAS
HQLLNILTKVIIDYLVGQVIAGAQLLQVFESHAGHLCARTFNTFALPYLSIIARQVEEKVRAQGKEPVPMIIFAKGAHYAIEDLAQSGYEVVGL
DWTVNPKTARHQTQGKVTLQGNMDPGALYASKEEIRTIVREMLEQFGTQQHIANLGHGIYPTIDPDHVEAFVDAVHSISE
>CiUD
GMEVQMVPGKGPVFPKPLANPEDLKTLNQTPDVDKELGYVFDAIKLTRQKLDGKVPLIGFSGAPWTLMAYMVDGGSSATFSKARSWLFRSPNES
KALLDMLTDTTIKYLVNQVRAGAQALQVFESHAGILGSSTFAKFSLPYLAKIATQVKKQLSDLGIQRVPLIIFAKGAHYAIEELSATDYDVISL
DWTMDVKRARECAKKSKALQGNLDPAMLYAGVDEICSATKKMVEDFGVTGHIANLGHGMYPDLDPEHLKAFVNSVHSASE
>SpUD
GLTVEMIPGTGPSFPEPLTSPDDLSRLNAEVDINQALSYVFEALTLTRKELDGKVPLIGFSGAPWTLMVYCIEGGGSKTFSKAKKWLYTQPDAS
HKLLKILTRTIVDYLVGQVLAGAQLLQVFDSHAGALGYDKFCQFCLPYLQQIAKEVKEKLRAKSVEPVPMIVFAKGANYAISELSQCDYDVMSL
DWDVQPQIARAQAGPNFPLQGNLDPCAIYSSKEDISSYVAQMLERFGTQSYIANLGHGIYPDFDPEHVRAFIDSVHQHSE
>DmUD
GLTVEMHAGVGPVLPQPIVVPEDLKRLTPDGALSR-LSYVGDAITMMRHKLEGRVPLIGFTGAPWTLMGYMIEGGGSKTMSKAKAWLNEHPEDS
KLFLNLLTDAIVDYLEMQVKAGAQMLQVFESSAEHLSKEQFLQWCVPYLKRIRDELVDRLTKKAIPVVPMTLFAKGAGHSLKEQSELGYDVIGL
DWTVDPLEARNLVGPNITLQGNLDPQDMYRDPDELRNLTTEMVHKFGKSRYIANLGHGITPQTPITSMEVLVEAVHKAL-
>MmuUD
GMEVTMVPGKGPSFPEPLREEQDLERLRDPAVVASELGYVFQAITLTRQRLAGRVPLIGFAGAPWTLMTYMVEGGGSSTMAQGQRWLYQRPQAK
SKTFFFL----ITGFIGQAVSGIEALQLFESHAGHLGPQLFNKFALPYIRDVAKRVKARLREAGLAPVPMIIFAKDGHFALEELSQAGYEVVGL
DWTVAPKKARECVGKTVTLQGNLDPCALYASEEEIGQLVKQMLDDFGPQRYIANLGHGLYPDMDPEHVGAFVDAVHKHSR
>MgUD
GMEVVMVPGKGPTFPEPLKEVEDLLKLRQKVDVSAELGYVFQAVTLTRHSLEGKVPLIGFSGAPWTLMSYMVEGGGSATMAKAKSWLYRHPEAS
HRLLRLLTDVVTDYLVGQVAAGAQALQLFESHAGHLGPEQFQEFALPYIRDIARDVKSKLKAEALPLVPMIVFAKDAHYALRDLAQAGYEVVGL
DWTIQPQEARAQVGKGITLQGNLDPCALYAPKEKIGELVKKMLESFGTQRYIANLGHGLYPDMNPEHVGAFVEAVHAHSR
>TgUD
GMEVVMVPGKGPTFTEPLKEVEDLLKLRQKVDVTSELGYVFQAITLTRHSLEGKVPLIGFSGAPWTLMSYMIEGGGSTTMAKAKSWLYRHPEAS
HRLLRLLADVIIDYLVGQVAAGAQALQLFESHAGHLGPELFQDFALPYIQDIAQAVKSKLKEEALPLVPMIIFAKDAHYALRDLAHAGYEVVGL
DWTIRPQEARAQIGKDVTLQGNLDPCALYAPKEKIGELVKKMLESFGTQRYIANLGHGLYPDMNPEHVGAFVEAVHAHSR
>AlUD
GMEVVMVPGKGPTFPEPLKGVEDLLKLRQKVDVAAELGYVFQAITLTRHRLEGKVPLIGFSGAPWTLMSYMIEGGGSNTMAKAKAWLYRHPEAS
HRLLTLLADVIADYLVGQVAAGAQALQLFESHAGHLGPEQFSEFCLLYIRSIAKQVKTKLHENAMPVVPLIIFAKDAHYALEDLAQSGYDVIGL
DWTIRPEDARQRIGKDITLQGNLDPCALYAPKEKIGELVKRMLDGFGTQRYIANLGHGLYPDMSPEHVGAFVDAVHAHSR
>OlUD
GMNVQMLTGKGPTFTEPLKEPEDLERLQAKVDVQKELGYVFKAITLTRHKLEGKVPLIGFTGAPWTLMSYMIEGGGSVTQSKAKRWLYRHPEAS
HMLLKMLADVIVEYLLGQVTAGAQALQVFESHAGILGPAQFKEFSLPYLRDIARRVKDRLKEAGQD-VPMIVFAKDAHYGLEDLSESHYEVVGL
DWTIDPQSARERTGGKVSLQGNMDPCALYAPKERISEIVKKMVAGFGTKGYIANLGHGLYPDMEPENVGAFVEAVHQHSK
>TrUD

GMNVQMVAGKGPTFPEPLKEPEDLQQLQAKVDVSKELDYVFKAITLTRHKIEGKVPLIGFSGAPWTLMSYMIEGGGSNTHSKAKRWLYRHPEAS
HMLLKMLTDVIVQYLLGQVAAGAQALQVFESHAGILGPVEFKEFSLPYLRDIARRVKDKLKEAGED-IPMIVFAKDAHYALEDLSQSHYEVVGL
DWTIDPQSARERTGGKVSLQGNMDPCALYAPKERISQIVKKMLEGFGKKGYIANLGHGLYPDMDPENVGAFVDAVHQHSK
>GaUD
GMDVQMVAGKGPTFPDPLKEPEDLQRLQVKVDVAKELGYVFKAITLTRHKIEGKVPLIGFTGAPWTLMSYMIEGGGSRTFSKAKSWLYRHPEAS
HMLLRMLTDVIVEYLLGQVAAGAQALQVFESHAGILGPSEFQEFSLPYLRDIARCVKDKLKEAGKD-VPMIVFAKDAHYSLEDLSQSRYEVVGL
DWTIDPQSARERTGGKVSLQGNMDPCALYAPKERISEIVKKMVEGFGPRGYIANLGHGLYPDMDPENVGAFVQAVHQHSK
>TnUD
GMSVQMVAGKGPTFPEPLKEPEDLQQLQAKVDVSKELDYVFKAITLTRHKLEGKVPLIGFSGAPWTLMSYMIEGGGSSTHAKAKRWLYRHPEAS
HMLLRMLTDVIVQYLLGQVAAGAQALQVFESHAGILGPAEFKEFSLAYLRDIARRVKEKLKEAGQD-VPMVVFAKDAHYALEDLSQSHYEVVGL
DWTVDPRSARRRTGGKVSLQGNMDPCALYAPKEHISRIVKKMLEGFGTKGYIANLGHGLYPDMDPENVGAFVEAVHRHSK
>CsUD
GMEVQMVPGKGPVFPKPLIHPNEIKALNQDPDIDKELGYVFDAITLTRKKLEGKVPLIGFSGAPWTLMAYMVEGSSSATFSKARAWLFQYPEES
ELLLNLLTNTIIKYLVNQVKAGAQTLQVFESHAGILGSSTFAKFSLQYLSRIASGVKKELSDLGISRVPLIVFAKGAHYALEELSATDYDVISL
DWTIDPKRARESSAKGKALQGNLDPAVLYGSPDSINCATKRMVEAFGARGHIANLGHGMYPDLKPESLKAFVDTVHSASQ
>CqUD
GITVEMKPGVGPVLPEPLVGPADLERLNQTGTMDR-LKYVGDAITMMRHMLEGRIPLYGFTGAPWTLMGYMIEGGGSKTMSKAKGWLADHPEAS
HKLLNILTDQVIEYLLLQVRAGAQILQVFESSAEHLSKEQYLTVALPYLKRIRDDLHRRFAEEGITPVPLTLFSKGAMHSLPEQAATGYDVIGL
DWTIDPEEARRLVGPNVTLQGNLDPQDMYKPKEELRELVLTMVRKFGKHRYIANLGHGITPQTPIESMEVLVQAVHDAL-
>AmeUD
GLTVEMIPGMGPVLPKPLKDPTDLVRL-IQPNVEKDLKYVGDAITLTRYKLEGKVPLIGFTGAAWTLMSYMIQGGGSSTMIRAREWLYKYPDDS
HKLLQLITNVIIDYLVMQVKAGAQI-------------------AFP---------------KGATINSLEMLAKSKT----------YEVLGL
DWTIDPIEARKKFGSEIILQGNFDPCALYGSEQEIINRAKDMALKFGKIRYIANLGHGILPDTPIASVTAFIKGIHSI--
>NvUD
GMTVEMVPGKGPHFPEPLNDPSDIEKLNQNANISEGLGYVYRAITLTRHKLEGKVPLFGFTGAPWTLMSYMIEGGGSTNFTKSRKWLYAHMEAS
SKLLQILTDAVVEHLVLQARAGAQILQVFESHGGLLGHDMFMLFSLPYLRQIAEKVKEKL---GPDAVPMTVFAKGAHYAIKELSQCGYDVVSL
DWTMSSKEAR-IVAPTVTLQGNLDPAALYGSHEDIVYAVKGMVRGFGIQRYIANLGHGMHPDHDPDKLKTFIDTVHSYSE
>AaUD
GITVEMHKGVGPVLPQPLVTPDDLERLNTTGSIDR-LKYVGDAITMMRHMLDGKVPLYGFTGAPWTLMGYMIEGGGSKTMSKAKAWLENYPEAS
TKLLNLLTDQIVDYLLMQVKAGAQILQVFESSAEHLSKEQYLNIALPYLKRIRDDLHKKFDEEGVTPVPMTLFSKGAMHSLTEQAQTGYDVIGL
DWTIDPEEARKLAGPNVTLQGNLDPQDMYKSPEELRELVLTMVRKFGKHRYIANLGHGITPQTPVESMSVLVQAVHDAL-
>AdUD
GMIVHMIPGKGPVFPDPLREPSDLEKLKKDVDLVKSLGYVFSAITLTRQKLEGKVPLIGFSGAPWTLMCYMIEGESSKTFNKARKFLRQHLQIS
QQLLQQMTDLIIDYLVLQVQAGAQMLQVFESHAEAIGYDTFTVCSLPYLKQIVQKVKEKL---GPDCVPMTIFAKGAHYAIKDLSQTGYDVVSL
DWTVLPKEAR-AAAPNVTLQGNLDPACLYASHEEICDAVKKMLENFGTQRHIANLGHGMHPDHEPEKLATFIDTVHSYSE
>LaUD
GMEVTMVPGKGPSFSEPLREEQDLEHLRDPAAAASELAYVFQAITLTRQRLDGRVPLIGFAGAPWTLMTYMIEGGSSSTMAKAKRWLYQRPQAS
HQLLRILTDTLVPYLVGQVAAGAQALQLFESHAGHLGPQLFTKFALPYIRDVAKRVKAGLQEAGLAPVPMIIFAKDGHFALEELAQAGYEVVGL
DWTLAPEKARELVGKTVTLQGNLDPCALYASEEEIRQLVQQMLDDFGPRRYIANLGHGLYPDMDPEHVGAFVDAVHRHSG
>OcUD
GMEVTMVPGKGPSFPEPIREEQDLERLRDPATVASELGYVFQAITLTRQRLAGRVPLIGFAGAPWTLMTYMIEGGGSSTMSQAKRWLYQRPQAS
HQLLQILTDALVPYLVGQVAAGAQALQLFESHAGHLGPQLFNKFALPYIRDVAKQVKARLQEAGLAPVPMIIFAKDGHFALEELAQAGYEVVGL
DWTVDPKKARERVGKTVTLQGNLDPCALYAPKEEIGQLVQQMLDGFGPQRYIANLGHGLYPDMDPEHVGAFVDAVHRHSR
>HmUD
GMQVEMQKGKGPVFPKPLLDATDIKLLNVDVDVKIELKYVLDAITLTRKTLDGKCPLIGFAGAPWTLMAYMIEGGGSKTYSKVKKCLYVDPDSC
KNLLNILTRVCIDFLVEQVKAGAQILQVFDSHAGELSADMFNIFCLPYLKDIATSVKKVLGPN--QSVPMIIFAKGSHYALKDLGESDYDVIAL
DWTMNIEDAR-RLCPHKTLMGNLDPCALYSSKEDIDTRVKKMVQMFGTSKYIANLGHGIYPDVDPDHMEAFVNAVHRHSQ
>DaUD
GLTVEMHAGVGPVLPQPIKVPDDLKRLTPDGALSR-LSYVGDAITMMRHKLDGRVPLIGFSGAPWTLMGYMIEGGGSKTMSKTKAWLKDYPEDS
KLFLNLLTDAIVDYLEMQVKAGAQMLQVFESSAEHLSKEDFLEWSVPYLRRIRDELVDRLNKKVIPVVPITLFAKGAGHSLKEQSELGYDVIGL
DWTVDPIEARNLVGPNITLQGNLDPQDMYRNPDELRNLTTEMVHKFGKSRYIANLGHGITPQTPLASMEVLVEAVHKAL-
>DpUD
GLTVEMHAGVGPVLPQPINTPEDLKRLTPDGALSR-LTYVGDAITMMRHKLEGRVPLIGFTGAPWTLMGYMIEGGGSKTMSKAKAWLSDHPEDT
KLFLNLLTDAIM-----QVKAGAQMLQVFESSAEHLTKEDFLIWAVPYLRRIRDELVDRLTKKAVPLVPITLFAKGAGHSLKEQSELGYDVIGL
DWTVDPIEARALVGPNITLQGNLDPQDMYRDADELRALTTEMVHKFGKSRYIANLGHGITPQTPITSMDVLVEAVHKAL-

#CPO#

>HsCO
MELLILETQAQVCQALAQVDG-GANFSVDRWERKEGGGGISCVLQDGCVFEKAGVSISVVHGNLSEEAAKQMRSRGKVLKTKDG-KLPFCAMGV
SSVIHPKNPHAPTIHFNYRYFEVEEADGNKQWWFGGGCDLTPTYLNQEDAVHFHRTLKEACDQHGPDLYPKFKKWCDDYFFIAHRGERRGIGGI
FFDDLDSPSKEEVFRFVQSCARAVVPSYIPLVKKHCDDSFTPQEKLWQQLRRGRYVEFNLLYDRGTKFGLFTPGSRIESILMSLPLTARWEYMH
SPSENSKEAEILEVLRHPRDWV
>MmCO
MELMIMETQAQVCRALAQVDG-VADFTVDRWERKEGGGGITCVLQDGRVFEKAGVSISVVHGNLSEEAANQMRGRGKTLKTKDS-KLPFTAMGV
SSVIHPKNPYAPTMHFNYRYFEVEEADGNTHWWFGGGCDLTPTYLNQEDAVHFHRTLKEACDQHGPDIYPKFKKWCDDYFFIVHRGERRGIGGI
FFDDLDSPSKEEAFRFVKTCAEAVVPSYVPIVKKHCDDSYTPRDKLWQQLRRGRYVEFNLLYDRGTKFGLFTPGSRIESILMSLPLTARWEYMH
SPPENSKEAEILEVLRHPKDWV
>DrCO
MEMLIMETQSAFCRALEQVD--GGSFRVDRWSRKEGGGGISCVMQDGKIFEKAGVNVSVVYGNLTEEAARQMRSRGKILKGKDG-KLPFRAMGV
SSVIHPKNPHIPTVHFNYRYFEIEEADGTKQWWFGGGTDLTPVYIDLEDATHFHKTLKEACDKHHPKYYPDFKKWCDNYFYIRHRGETRGIGGI
FFDDLDSPDQDEVFNFVRSCAKTVVPCYLPIVYKHLNDSFTPEEKDWQQVRRGRYVEFNLVYDRGVKFGLATPGSRIESILMSLPLTARWEYMH
EPTKGTKEAEMLEILRNPKQWI
>GgCO
MELLIMETQAEVCHALAALDP-GASFAVDTWERKEGGGGISCVLQDGEVFEKAGVNVSVVFGQLSEEAARQMRSRGKALKATEDGKLPFCAMGV
SSVIHPKNPHVPTMHFNYRYFEIEEADGTKQWWFGGGTDLTPTYLNEEDAVHFHKTLKEVCDKHDLKLYPKYKKWCDDYFHIKHRGERRGIGGI
FFDDVDSPSKEEVFQFVQSCAKAVVPSYVPIVKKHCHDSYTPEEKLWQQLRRGRYVEFNLVYDRGTKFGLLTPGSRIESILMSLPLTARWEYMN
TPSESSKEAEILEVLRNPKDWV
>XtCO
MELMIMGTQAEVCRALAQADG-AASFTVDRWQRAEGGGGISCVLQDGKVFEKAGVNVSVVHGYLSEESIRQMKSRGKSLKTKDG-KLPFCAMGV
SSVIHPKNPHVPTIHFNYRYFEIEEADGNRQWWFGGGTDLTPTYLDQEDVVHFHQTLKDACDKHSPTFYPKYKKWCDDYFFINHRGESRGVGGI
FFDDLDFASKEELFAFVESCAKAVVPCYLPIVEKHKNDSFTPEEKLWQQLRRGRYVEFNLVYDRGTKFGLATPGSRIESILMSLPLTARWEYNH
IPPEDSKEGEILKVLRKPRDWV
>BtCO
MELLILETQAQVCQALAQVDG-GARFSVDRWERKEGGGGISCVLQDGHVFEKAGVSISVVHGNLSEEAAKQMRSRGKLLKTKDG-KLPFSAMGV
SSVIHPKNPHTPTIHFNYRYFEVEEADGNTLWWFGGGCDLTPTYLNQEDAVHFHRTLKEACDQHGPDLYPKFKKWCDDYFFIAHRGERRGIGGI
FFDDLDSPSKEEVFRFVQSCTQAIIPSYVPLVKKHCNDSFTPQEKLWQQLRRGRYVEFNLLYDRGTKFGLFTPGSRIESILMSLPLTARWEYMH
SPSENSKEAEMLEVLRHPRDWV
>DmCO
MEILIMEIQAEFCRALEAEENCGQKFKVDRWERPEGGGGITCVLQDGDVFEKAGVNISVVTGSLPPAAVQQMRARGKNLK--EGASLPFFASGV
SAVIHPRNPHVPTIHFNYRYFEVETAKGEKQWWFGGGTDLTPYYLCEKDASHFHQTLKSACDEHDPTYYPRFKKWCDDYFRIKHRNESRGIGGI
FFDDIDSPNQEAAFNFVSSCARAVIPSYVPLVRKHKNREYGNNERQWQLLRRGRYVEFNLIYDRGTKFGLYTPGARYESILMSLPLHARWEYMH
EPKSQSEEGKLMKVLKNPKDWV
>ClfCO
VRVVLEQPDSPDSPVSPSPPFLGRIHPFSSWRKREAGGGISCVLQDGHVFEKAGVSISVVHGNLSEEAAKQMRSRGKNLKTKDG-KLPFSAMGV
SSVIHPKNPHAPTIHFNYRYFEVEEADGNKQWWFGGGCDLTPTYLNQEDAVHFHRTLKEACDQHGPDLYPKFKKWCDDYFFIVHRGERRGIGGI
FFDDLDSPSKEEVFRFVQSCAQAVVPSYIPLVKKHHNDSFTPQEKLWQQLRRGRYVEFNLLYDRGTKFGLFTPGSRIESILMSLPLTARWEYMH
SPPENSKEAEILEVLRHPRDWV
>CiCO
MELMIMETQSKVCKAIESLEAEGQKFEVDKWNKESGGGGVTCVLQDGKVFEKAGVNVSVVHGSLTEGAEKQMRARGRDFKRFDDGSLPFIAMGV
SSVIHPTNPHVPTIHFNFRYFEVTGADKKKTWWFGGGTDLTPMYLDQNDVKHFHTELKKACDKHDVQYYPKFKKWCDEYFTIPIRGERRGVGGI
FFDDIEGPDEESALAFVTSCAEAIIPCYVPIVVKNKNKSFTSEQKQWQQLRRGRYVEFNLIYDRGTKFGFQTPGARIESILMSLPLTARWQYMH
TPKLGSAENELLDVLKSPREWV
>SpCO
MELFIMKTQAKICKALEEIEG-EKKFKIDRWERAEGGGGITCILEDGEVFERAGVNISVVHGKLSPKAAAQMRARGKPLT--PGKELPFFATGI
SSVVHPRNPMIPTLHFNYRYFEVEQ-DGESQWWFGGGTDLTPYYLDEEDATHFHRTLKAACDLHDPEYYPKFKEWCDNYFVVEHRGERRGLGGI
FFDDIDQPTQEKAFSFVKSCATAILPCYIPIIDKHQKDPYGYKESL---------------FDRNI-----------------------------
-----SEE--------------
>MmuCO
MELLILETQAQVCQALAQVDG-GASFSVDRWERKEGGGGISCVLQDGCVFEKAGVSISVVHGNLSEEAAKQMRSRGKVLKTKDG-KLPFCAMGV
SSVIHPKNPHAPTIHFNYRYFEVEEADGNKQWWFGGGCDLTPTYLNQEDAVHFHRTLKEACDQHGPELYPKFKKWCDDYFFIAHRGERRGIGGI
FFDDLDSPSKEEVFRFVQSCAKAVVPSYIPLVKKHCDDSFTPQEKLWQQLRRGRYVEFNLLYDRGTKFGLFTPGSRIESILMSLPLTARWEYMH
SPSENSKEAEILEVLRHPRDWV
>MgCO
MELLIMETQAEVCRALAALDP-GASFAVDTWERKEGGGGISCVLQDGEVFEKAGVNVSVVFGQLSEEAARQMRSRGKALKATEDGKLPFCAMGV

SSVIHPKNPHVPTMHFNYRYFEIEEADGSKQWWFGGGTDLTPTYLNEEDAVHFHRTLKEVCDKHDLKLYPKYKKWCDDYFYIKHRDERRGIGGI
FFDDVDSPSKEEVFQFVQSCAKAVVPSYVPIVKKHCHDSYTPEEKLWQQLRRGRYVEFNLVYDRGTKFGLLTPGSRIESILMSLPLTARWEYMN
TPPESSKEAEILEVLRNPKDWV
>TgCO
MELLIMETQAEVCRALADLDP-GASFAVDTWERKEGGGGISCVLQDGEVFEKAGVNVSVVSGLLSEEAARQMRSRGKSLKAKDG-KLPFCAMGV
SSVIHPKNPHVPTMHFNYRYFEIEEADGTKQWWFGGGTDLTPTYLNEEDAIHFHKTLKEACDKHDVKLYPKYKKWCDDYFYIKHRGERRGIGGI
FFDDMDSPSKEEVFQFVKSCAKAVVPCYIPIVKKHCHDSFTPEEKLWQQLRRGRYVEFNLVYDRGTKFGLLTPGSRIESILMSLPLTARWEYMH
NPPENSKEAEILEVLRNPKDWV
>AlCO
---MRSRGKSLKAKNG-KLPFCAMGV
SSVIHPKNPYVPTIHFNYRYFEIEEADGTKQWWFGGGTDLTPTYLNKEDAVHFHKTLKEACDKHNPDLYPKFKKWCDDYFYIKHRGERRGIGGI
FFDDLDSPSKEEVFQFVESCAKAIVPCYIPIVKKHCHDTFTPEEKLWQQIRRGRYVEFNLVYDRGTKFGLATPGSRIESILMSLPLTARWEYMH
SPPENSREAEILEVLRHPKDWA
>OlCO
MELLIMETQAEFCKALEEVD--GGTFKVDRWERKEGGGGISCVMQDGKVFEKAGVNVSVVSGYLTEEAAKQMRSRGKVLKGKDG-KLPFSAMGV
SSVIHPKNPHIPTVHFNYRYFEIEEEDGSKQWWFGGGTDLTPVYIDKEDAFHFHNTLKEACDKHHPQYYPDFKKWCDRYFFVRHRGETRGIGGI
FFDDLDSPGQEEAFNFVKSCARTVVPCYLPIVYKHLNDSFTDEEKDWQQIRRGRYVEFNLVYDRGVKFGLATPGSRIESILMSLPLTARWEYMQ
EPDKGSREAEMLEVLRNPKEWV
>TrCO
MEMLIMETQAEFCKALEEVD--GGTFQVDKWQRKEGGGGISCVMQDGKVFEKAGVNVSVVSGYLTEEAAKQMRSRGKALKGKDG-KLPFCAMGV
SSVIHPKNPHIPTVHFNYRYFEIEEEDGSRQWWFGGGTDLTPVYVNKDDAFLFHNTLKEACDQHHPQYYPDFKNWCDRYFYVRHRGETRGIGGI
FFDDLDSPSQEEAFSFVKSCARTVVPCYLPIVYKHLNDPFTQEEKAWQQVRRGRYVEFNLVYDRGVKFGLATPGSRIESILMSLPLTARWEYMH
EPAEGSREAEMLEVLRNPKEWV
>GaCO
MEMLIMETQADFCNALQEVD--GGTFKVDRWQRNEGGGGISCVMQDGKVFEKAGVNVSVVFGNLTEEAARQMRSRGKVLKGKDG-KLPFCAMGV
SSVIHPKNPHIPTVHFNYRYFEIEEEDGTKQWWFGGGTDLTPIYVSKEDASHFHNTLKEACDKHHPQYYPDFKKWCDRYFYIRHRGETRGIGGI
FFDDLDSPSQEEAFDFVKSCARTVVPCYLPIVRKHLNDSFTDEEKAWQQVRRGRYVEFNLVYDRGVKFGLATPGSRIESILMSLPLTARWEYMH
EPAKGTLEAEMLEVLRNPKEWV
>TnCO
MEMLIMETQAEFCRALEEVD--GGTFQVDKWQRKEGGGGISCVMQDGKVFEKAGVNVSVVSGYLTEEAAKQMRSRGKSLRGRDG-KLPFCAMGV
SSVIHPKNPHVPTVHFNYRYFEIEEEDGSKQWWFGGGTDLTPVYINPEDASLFHGTLKEACDRHHPRYYPDFKKWCDRYFYIRHRGETRGIGGI
FFDDLDSPSQEEVFSFVKSCARTVVPCYLPIVRKHLNDPFTQEEKDWQQVRRGRYVEFNLVYDRGVKFGLATPGSRIESILMSLPLTARWEYMH
EPAEGTPEAEMLAVLRNPEEWV
>CsCO
MEMMIMETQSKVCKAIEALEDGGQKFLVDKWSKDNSGGGVTCVLQDGTVFEKAGVNVSVVHGNLTKGAEKQMRARGKQFQRLKDGTLPFIAMGV
SSVIHPTNPHCPTIHFNFRYFEVTGRNGEKTWWFGGGTDLTPMYLDENDVKHFHTELKKACDVHDKEYYPNFKTWCDDYFMITIRGERRGVGGI
FFDDIEGPDAQSALEFVTSCANAIIPCYVPIITKNKYKSFTEEQKQWQQLRRGRYVEFNLIYDRGTKFGFQTPGARIESILMSLPLTARWQYMH
TPKLGSMENQLLDVLKSPRDWV
>CqCO
MELLIMKIQADFCNALEGEENFGKKFTVDRWERSEGGGGITCVLQDGDVFEKAGVNISVVHGNLPKGAIQQMRSRGKQLA--DG-ELPFFAAGV
SAVIHPRNPMVPTIHFNYRYFEVTDSQGTKQWWFGGGTDLTPYYLNEEDAIHFHRTLKEACDEHDSSYYPKFKAWCDKYFFIPHRNECRGVGGI
FFDDLDEPDQESAFNFVSSCAHSVIPSYLPLVKKHKNEAYGDRERQWQLLRRGRYVEFNLIYDRGTKFGLYTPGARYESILMSLPLTAKWEYMN
LPPADSKESEITHVLKNPKEWL
>AmeCO
MELLVMTTQADFCKALESLEDPNHRFKVDRWIRKEGGGGITCVLQDGVVFEKAGVNVSVVTGTLPPGAVQQMRARGKNMQ--EG-SVPFFAAGV
SAVIHPRNPMIPTIHFNYRYFEVENSDGSIQWWFGGGTDLTPYYLNEDDIKQFHKTLKIACDKHDLSFYPKYKKWCDDYFFITHRGERRGVGGI
FFDDIDTPSQEEAFQFVKSCAEAVIPSYIPLVEKHKNDRYGYAERQWQLLRRGRYVEFNLIYDRGTKFGLYTPGARYESILMSLPLSAKWHYMH
EPEPDSKEAELVNVLRNPKEWL
>AaCO
MELMIMKIQADFCQSLENEENFGKKFTVDRWERKEGGGGITCVLQDGDVFEKAGVNISVVHGVLPKGAIQQMRSRGKQLA--DG-ELPFFAAGV
SAVIHPRNPMVPTIHFNYRYFEVTDSKGNKQWWFGGGTDLTPYYLDEKDAVHFHQTLKEACDEHDESYYPKFKEWCDKYFYIPHRNECRGVGGI
FFDDIDTPDQESAFNFITTCAHSVIPSYLPLVRKHKNDAYGDRERQWQLLRRGRYVEFNLIYDRGTKFGLYTPGARYESILMSLPLTAKWEYMH
VPAPDSKEGEITHVLKNPRDWL
>LaCO
MELLIMETQAQVCQALAQVDG-GASFSVDRWERKEGGGGISCVLQDGHVFEKAGVSISVVHGNLSEEASKQMRSRGKILKTKDG-KLPFSAMGV
SSVIHPKNPHAPTIHFNYRYFEVEEADGNKQWWFGGGCDLTPTYLNQDDAVHFHRTLKEACDKHGPDLYPKFKKWCDDYFFIAHRGERRGVGGI
FFDDLDSPSKEEVFRFVQSCAQAVVPSYIPLVKKHCNDSFTPQEKLWQQLRRGRYVEFNLLYDRGTKFGLFTPGSRIESILMSLPLTARWEYMH

SPSENSKEAEILEVLRHPRDWV
>OcCO
MELLILETQAQVCQALAQVDG-GASFSVDRWERKEGGGGISCVLQDGRVFEKAGVSISVVHGNLSEEAAKQMRSRGKTLKKTKDGKLPFCAMGV
SSVIHPKNPHAPTIHFNYRYFEIEEADGNKQWWFGGGCDLTPTYLNQEDAVHFHRTLKEACDQHGPDLYPKFKKWCDDYFFIAHRGERRGIGGI
FFDDLDSPSKEEVFRFVQSCAQAVVPSYIPLVKKHCDDSFTPQEKLWQQLRRGRYVEFNLLYDRGTKFGLFTPGSRIESILMSLPLTARWEYMH
SPSENSKEAEILEVLRHPRDWV
>NvCO
MEVMILKAQGEICRKLSDLD--GKKFQVDRWERQQGGGGMTCVIQDGKVFEKAGVNVSVVYGTLAAKAAQQMKSRGKGFK---GEDLPFFACGI
SSVIHPRNPYVPTVHFNYRYFEVTDIDGQKHAWFGGGTDLTPYYLNEQDVMHFHGVLKRVCDKHDASYYQRFKSWCDKYFFIPHRGECRGVGGI
FFDDLEDPTEEKLFNFLESCAEAVIPAYAPIVEKHMNDPYSEEDRKWQLLRRGRYVEFNLVYDRGTKFGLYTPGSRIESILMSLPLEARWEYMH
VPDKNSREEKMLEVLRNPREWI
>DaCO
MELLILEIQAEVCRALEAEENCGQRFEVDRWERAEGGGGITCVLQDGDVFEKAGVNISVVTGWLPPAAVEQMRARGKKLN--SGKKLPFYASGV
SAVIHPRNPHVPTIHFNYRYFEVETAKGEKQWWFGGGTDLTPYYLCEKDANHFHQTLKSACDEHDATYYPRFKKWCDDYFRIKHRNESRGIGGI
FFDDVDTPNQESAFKFVSSCAKAVIPSYMPLVRKNKNREYGSNERQWQLLRRGRYVEFNLIYDRGTKFGLYTPGARYESILMSLPLHARWEYMH
EPKSKSEESKLLKVLRNPKEWV
>DvCO
MELMIMEIQAEFCRALESEEFSGQKFKVDRWQRKEGGGGVTCVLQDGEVFEKAGVNVSVVTGMLPPAAVQQMRARGKELK--ENVKLPFFASGV
SAVIHPRNPHVPTIHFNYRYFEVELPDGKKQWWFGGGTDLTPYYLDENDARHFHQTLKSACDEHDTSYYPRFKKWCDDYFHIKHRNESRGIGGI
FFDDIDEPSQDSAFNFVTSCARAVIPSYLPLVQKHKNADYGNNERQWQLLRRGRYVEFNLIYDRGTKFGLYTPGARYESILMSLPLHARWEYMH
EPKSTSKENDLLMVLRNPKEWV

#PPO#
>HsPO
RTVVVLGGGISGLAASYHLSRAPCPPKVVLVESSERLGGWIRSVRGPNGAIFELGPRGIRPAGALGARTLLLVSELGLDSEVLPVRGDHPAAQN
RFLYVGGALHALPTGLRGLLRPSP-PFSKPLFWAGLRELTKPRGKEPDETVHSFAQRRLGPEVASLAMDSLCRGVFAGNSRELSIRSCFPSLFQ
AEQTHRSILLGLLLGAGRTPQPDSALIRQALAERWSQWSLRGGLEMLPQALETHLTS-RGVSVLRGQPVCGLSLQAEGRWKVSLRDSSLEADHV
ISAIPASVLSELLPAEAAPLARALSAITAVSVAVVNLQYQGAHLPVQGFGHLVPSSEDPGVLGIVYDSVAFPEQDG-SPPGLRVTVMLGGSWLQ
TLEASGCVLSQELFQQRAQEAAATQLGLKEMPSHCLVHLHKNCIPQYTLGHWQKLESARQFLTAHRLPLTLAGASYEGVAVNDCIESGRQAAVS
VLG
>ClfPO
RTVVVLGGGISGLAAGYHLSRAPCAPKVVLVEGSERLGGWIRSVRGPGGAVFELGPRGIRPAGAPGARTLLLVSELGLDSEVLPVRGDHPAAQN
RFLYVGGALHALPSGLRGLFRPSP-PFSKPLFWAGLRELTTPRGKDPDETVHSFAQRRLGPEVASLAMDSLCRGVFAGNSRELSVRSCFPSLFQ
AEQSHRSVLLGLLLGAGHSPRPDSALIRQARAERWSQWSLRGGLETLPQALNTYLTS-RGVSVLRGQPVCGLGLLPGGRWKVSLGDGSLEADHV
ISAVPASVLSKLLPAQAAPLAHALSAITAVSVVVVNLQYRGARLPVQGFGHLVPSSEDPGVLGIVYDSVAFPEQDG-SPPGLRVTVMLGGSWLQ
TLEARGGLLSRELFQQQAQQAAATQLGLKEPPSHCLVHLHKNCIPQYTLGHWQKLEAVTQFLASQRLPLTLAGASYEGVAVNDCIESGRRAAAR
VLG
>BtPO
RTVVVLGGGISGLAASYYLSRAPCPPKVVLVEGSERLGGWIRSVRGPDGAIFELGPRGIRPAGALGARTLLLVSELGLDSEVLPVRGDHPAAQN
RFLYVGGALHALPSGIRGLLRPSP-PFSKPLFWAGLRDLTTPRGKDPDETVHSFAQRRLGPEVASLAMDSLCRGVFAGNSRELSIRSCFPSLFQ
AEQTHRSILLGLLLGAGRGPQLDSALIRQAQAERWSQWSLRGGLETLPQALHAHLTS-RGVSVLQGQPVCGLSLQAEGRWKVSLEDSSLEADHI
ISAIPASVLSKLLPAEATPLARALSTITAVSVAVVNLQYRGARLPVQGFGHLVPSSEDPVILGIVYDSVAFPEQDG-SLPGLRLTVMLGGSWLQ
TLEARGCVLSQELLQQEAEKAAATQLGLNEPPSHCLVHLHKNSIPQYTLGHWQKLESAAQFLAAQKLPLTLAGASYEGVAVNDCIESGRQAAAR
VLG
>MmPO
RTVIVLGGGISGLAASYHLIRGPSPPKVILVEGSKRLGGWIRSIRGSDGAIFELGPRGIRPAGALGARTLLLVSELGLESEVLPVRGDHPAAQN
RFLYVGGTLHPLPSGLRGLLRPSP-PFSKPLFWAGLRELLKPRGKEPDETVHSFAQRRLGPEVASLAMDSLCRGVFAGNSRELSIRSCFPSLFQ
AEQTHRSILLGLLLGAGQSPQPDSSLIRQARAERWSQWSLRGGLEVLPQALHNHLAS-KGVTVLSGQPVCGLSLQPEGRWKVSLGDSSLEADHI
ISAIPASELSKLLPAEAAPLARILSTIKAVSVAVVNLQYRGACLPVQGFGHLVPSSEDPTVLGIVYDSVAFPEQDG-NPPSLRVTVMLGGYWLQ
KLKAAGHQLSPELFQQQAQEAAATQLGLKEPPSHCLVHLHKNCIPQYTIGHWQKLDSAMQFLTAQRLPLTLAGASYEGVAVNDCIESGRQAAVA
VLG
>DrPO
KVVAVLGGGIGGLSACHHLSKSPNVSKIVLLEGSGRCGGWLSSVRRDDGAVFEQGPRGVRPGGAVGRNTLNMVSELGLESELLPITSDHLASQN
RFLYVKGQLHKMPSGLGGVLRTIP-PFSRPIIQSVLKELVISKGTEEDESVHAFVSRRLGSELADIAIDCLCRGVFAGDSRQLSVRSCFPPLYE
AEQARGSIVLGMLMGSGAGPKVVPSLAKRASKESWTQWSLKRGMQTLPEALEDSLRRRNGVELHHHAKVKRLNVDST-GWEIKLDDGTSKADHV
ISTLPASALASVLPPAAQTLSEQLRSIASVNVALVNLEYEGFILPVTGFGHLVPSTEDAGVLGVVYDSVPFPEHNRSRGATTRLTVMMGGAWFE
QTFGSPDLVMEQTLLDRAVQAVNSHLSVTSQPVWSCVALLKNCIPQYHLGHWKRLEKMRQYISNHNLALTLAGASYDGVSVNDVIFSGRTAAEG

LVG
>DmPO
MTTAVLGGGLSGLSAGYYLLRRFGKP-LTIYEASPRVGGWVRSENRKDNFIFESGPRTIRPVGEPGANTLELVED--LKLEVTPIRRSHVAARN
RMLYAKGQLCMLPNSPKGLFGVLP-PFTKPLYKAVLRDLFTASKKAEDESIYSFAERRFGKEIADYAISPMICGICAGDAREISVRFLMEGLFE
KEQKYGGVLKGTLISRNKTKDTKDGLYAQAVKEKWAMYGLKGGLENLPKTMRKYLGE-RDVNVQLSNECRNLTFSSS-GVRMNIKDAEVPVEHV
VSSLPAYKLAPLVKQQHPSLSAQLLSIPYVDVLVVNMQFPGKLLKQDGFGLLVPPVEKLPLLGVIFDSCCFDMGEN-----TVLTVMMGGHWFD
QWFGDRP--SPKQILDLATSHVQKMLQIREEPKFSRVHTLHKCIPQYTVGHKRRVEAIRNYIKTYKLPLSVCGAAYDGVGINDVILSARRQVEA
IPL
>XlPO
RTVVVAGGGISGLTACYHLAKDARVSKVILLEGSSRLGGWMHSTRTEDGAVFEHGPRGIRPGGVVGRNTLCMVSELGLQQEILPVPRSHPAAKD
RYLYVNKSLHKLPSSVGGVLRTIP-PFTRPLFLCGLRDLAAPRGSKEDESIYDFVARRFGKELADIVIDSLCRGVFAGDCRSLSVRSCFPFLYE
AEVKRRSVILGMATAGEKGPPVDSELIRRSKQERWSQWSLEGGMQTLSEALEDFLRN-RGVEIHKDTPIRQLERTAGNGWKIKLPDGSISADYI
FSALPAKDLSSLLPQDLEPLVKELQQMSSATVAVVNLQYEGDVLPISGFGHLIPSSEDRALLGIVYDSIAFPQHNKRGSSSTRLTVMLGGAWFQ
SSIGDPDTVSSEKVLELATTAAAEQLGVREKPSRSIVNINKDCIPQYTLGHWSRTGNLSTYIRQVNLPLSLVGASYHGVSVNDCIYNAKQAVHN
LLG
>BfPO
TSVAVIGGGISGLSAAYYLSKAPHLVKVVLLEGSSRLGGWLQSTRTEEGAIFEHGPRSLRVAGEPGANILGMADDLGLTDQILPVLPSHEGAKN
RFIYAGGKLHKLPSN----FRWVS-PTNIPVPVCGHRHVCEGREEEGDESVHSFFCRRLGKQFTDNAIDPMVRGIYGGDCRQLSVQALFPSIQQ
AERRKGSITRGLLFGPKKKESPLKVLLKKARQERWALWSLKDGLEALSNSLQEYLMK-SGVELLTDRRVETLEFDTT--NQVNTLDEQLQVNHV
ISAVPSQCLSQMFAGNHPILSENLSVNPSATIGLVNLEFSGSVLPSEGFGYLVPSGQPERILGAVFDSSIFPQHNRPNSATTRLTVMMGGTWFC
EQFGDPNEVDTSLLLDVALETMSRHLNITSSPLRSFTTIQKDCIPQYTLGHTDRLEQMESYIAEKRLPLSLVGCSYRGVGVNDCVLDARKAVTN
LLK
>CiPO
MKFVVCGGGISGLASAYYLARTTSKPKIFLIEKSTNVGGWMKTNVRNGGAINERGPRSVRTAGLVGNNTLQLISDIGMESNVIPVRSDDIAAKQ
RLLYVNKKLHILPNSLLSACKKQQ-PFSNSLARSFVGGLLKKVPP-SYETMSEICSERFGREVADYLVSAFVRGVFAADSKQLSMKAAFPPLWE
AQMPRRPTLKNPLFNKSLKSNP---FIKRAKREKWTQWGVDNGFSYLTDNLEQQLAQVDNIEIIKYTNIDEIMMQNNGKMMMSTTTEQINIDHT
ISALPSYALSRILKNSNKTLGIMLSHIKWVDVGVVNVEFSNEILPHVGFGHLVPSSEPSSVLGIVYDSCAFPQHDRVNMPTTRMTCMMGGSWFR
ELFGEPNLVSNQTLESAALGSISQQLGIHEDPMNVDTTLCKKCIPTYRVGHTDLVENIEDFISEHDVPLSLVGASYWGVSINDCIFNSRVAVNN
ILK
>SpPO

--MRACFPAIFD
AERKYGSIIIGGFRSKPEKFPDIGTIAQRAKEQKWASWTMTDGLQTFPNKLSDWLVE-NGVEVRRSNPCSRIEFDGQKRVRVVTENQDVEADHI
VCSTPAQVTQRLLPPEHQTLSNLLSQITSSNVAVVNLEYNNLQLPVTGFGYLVPSSVQSKILGVVFDSCAFPELDRRKNPTTRMTCMMGGAWFH
DLFGDPHSVDPSDLLSCALTSIKDQLGISQKPTFTKVNIHHDCIPQYKLGHTELVDDIHLYIKDHRLPLSIVGSSYKGVSVNDCIFQARQAVTS
L--
>MmuPO
RTVVVLGGGISGLAASYHLSRAPCPPKVVLVEGSERLGGWIRSVRGPNGAIFELGPRGIRPAGALGARTLLLVSELGLDSEVLPVRGDHPAAQN
RFLYVGGALHALPTGLRGLLRPSP-PFSKPLFWAGLRELTKPRGKEPDETVHSFAQRRLGPEVASLAMDSLCRGVFAGNSRELSIRSCFPSLFQ
AEQTHRSVLLGLLLGAGRTPQPDSALIRQALAERWSQWSLRGGLEMLPQALETHLTS-RGVSVLRGQPVCGLSLQAEGRWKVSLRDSSLEADHV
ISAIPASVLSELLPAEAAPLARALSAITAVSVAVVNLQYQGAHLPVQGFGHLVPSSEDPGILGIVYDSVAFPEQDG-SPPGLRVTVMLGGSWLQ
TLEASGCVLSQELFQQRAQEAAATQLGLKELPSHCLVHLHKNCIPQYTLGHWQKLESARQFLAAHRLPLTLAGASYEGVAVNDCIESGRQAAVS
VLG
>AlPO
RTVAVLGGGVSGLSACFYLSRSPQAPKVILLESSSRLGGWISSTRTEDGAVFEHGPRGVRPAGNVGKNTLLMVATFGLSIQSIPLPQSKTSQQ-
------MAIQSLHKNLQGSIQSIPLPQSNPTRQMAIQSLHQKPPGKNDDNSWFVPHWRESEELADIVIDSLCRGVFAGDCRALSVRSCFPALFQ
AERAHRSVILGMTLAGGKGAPALDSLIRRAKEERWSQWSLRGGMQTLPEALEAFLKQ-RGAEIHRDTPVKRMERTASGSWRIMLDDGSIEADHV
ISALPAKALAASLPAWAEPLSRELLNIRAVSVAVVNLAYGNVGLPVMGFGHLVPSFEDNALLGIVYDSVAFPEQDGSGGPAIRLTVMLGGAWFK
LGLGDPDTVSHAMLLSCAKAAVKKHLGIHAEPSRAIVKVQKACIPQYEIGHWKHIENAATYLVQQKLPLSLIGASYEGVSVNDCISGARAAAVK
LLG
>OlPO
RTVAVLGGGAGGLAASYYLCRSLQVTKVILIESSSRFGGWLKSTRRPDSAVFEQGPRGIRPAGAVGQNTLNMVDDLGLGREVLPVLYSHVASKN
RFLYVNGRLNRMPSGLRGLLRTTP-PFSRPLLYSLASEILVKKSKEEDESVHSFVSRRLGQELADLAVDSLCRGVFAGDCRKLSVRSCFPPLFK
AEQQMGSLILGMLLGSGSTATVTPGLAKKAKKESWAQWSLRGGLETLPESIAEYLQRSGKAEIQKNAPVKNISHSEL-GWKIQLEDGVVSADHI
ISALPAKALACVLPPSCQSLTQMLQDVHAVTVAVVNLEYDGSILPVQGFGHLLPSSEDKGLLGVVYDSVPFPEHNQPDRETTRLTVMMGGAWFQ
EVFGDPESVTSEHLLKQACEAVCCQLGVKAAPIWSHVSLQRDCIPQYYLGHSQRVESMRSFIRSRNLPLSLVGSSFDGVSVNDVIFSGRAAVEE
LFG

>TrPO
KTVAVLGGGIAGLAASYYLCRSPQVTKVILLESSSRVGGWLSSTRRPDGAVFEHGPRGIRPAGAVGRNTLNMVDDLGLQDEIVPVSYSHVASKN
RYLYVNKELHKMPSGIRGLLQTVP-PFSRPLLFNLVKEILVKKGQEDDETIHSFFCRRLGKEFADIAVDSLCRGVFAGDCRKLSVRSCFPLLYN
AEQRRGSVLLGMMLGSGPSPVVPPGLALRSVKESWAQWSLRRGVESLPESIAELLQRSGKVELHTDAPVKEIHPSAS-DWKIRLEDGLISADHI
ISALPAKALSSVLPSSCLPLTQRLMEIETVTVAVVNLEYEGSVLPVEGFGHLLPSSEDQAVLGVVYDSVAFPQHNRPTGETTRLTVMMGGAWFH
RAGGSLGAVTQGDLLSRATEAVRDHLDVTAAPIWSHVEIQKDCIPQYYEGHAQRVESMRNFIKKENLPLSLIGSSYDGVSVNDVIFSGRTAAEE
LLG
>GaPO
TTVAVLGGG-GGLAASYYLCKSPQVTKVVVLESSSRLGGWLWSTRRSDGAVFEHGPRGIRPAGAVGRNTLNMVEDLGLGGEILPVTYSHVASKN
RYVYVNRRLHRMPSGMSGLLRTVP-PFSRPLLLNIAGEILVRKGKGEDESIHSFVSRRLGKELADIAVDSLCRGVFAGDCRKLSVRSCFPALYH
AEQRRGSLTLGMLLGSGPTAVVPPGLAQRSVDESWAQWSLRRGVESLPESIGEYLRHSGRAELHREAAVQQISPSAS-GWKITLEDGVMSADHI
ISALPAKALSSILPSSCQPLIQLLQDIATVTVAVVNLEYEGSVLPVQGFGHLLPSSEDRGLLGVVYDSVPFPQHNRPEGQTTRLTVMMGGAWFQ
EVFGDPAAVTEERLLATATEAVSRQLGVSSAPSWSRVTLQKDCIPQYYQGHYRRVESMRSFIRDKSLSLSLIGSSYDGVSVNDVIFSGRTAAEE
LLG
>TnPO
KTVAVLGGGVAGLAASFYLCRSPQVTKVILVESSGRLGGWLSSTRRADGAVFEHGPRGIRPAGALGRNTLNMVDELGLQEEIVPVPSSHAASRN
RYLYVNQKLHRMPSGLRGLVRTVP-PFSRPLLLNLAQEMLVKKGQQEDETIHSFFSRRLGQELADIAVDSLCRGVFAGDCRRLSVRSCFPPLFQ
AEQRRGSLLLGMMLGSGPGPVVPPGLALRSVKESWAQWSLRRGVESLPEALAEFLRRSGKVELHVHAPVAQIQPSAS-GWKICLEDGVLSADHV
ISALPAKALASALPPSCRPLTQQLKGIETVTVAVVNLEYEGSVLPVEGFGHLLPSSEDPALLGVVYDSEAFPQHNRPTGTTTRLTVMMGGAWFH
AVGGPLGAVPEEALLSRATEAVHAHLHVSAAPIWSHVSVQKDCIPQYYQGHSGRLASMRSFIRKENLALSLIGSSYDGVSVNDVIYGGRTAAEE
LLG
>CqPO
-MTAILGAGISGLSAGYYLLKK-GLPPPVIYEASSRIGGWIRTERFDGGYVFEAGPRTIRPKGPAARNTLDLIEEVGLGEHVHPIFSNHVAARN
RMIYAKGRLNMLPSDLSGVVKTVP-PFTKPLFFAGLKDLFGGKSRKDDEAMYSFVERRFGKEIADYAISSMLCGICAGDAKQISVKFLMKSLFE
VEQKHGGVVKGMLMEAKDKNSSKQVLAKRAKSENWSIYSIQGGLQTLPNRLKSVLDE-GQVDIRTDVKCEEIEFKGN-KVELRVDGEERTLDRL
ISSIPSYKLAKYLAKQHPILARELAAIPFVDVGVINLRFKNDLLKQKAFGFLVPPIEKLPILGCIFDSCCFDMEDN-----TVLTVMMGGAWFR
QWFGENP--SEEQLLEVALKQVNQILNINQKPDSYKVNILRRCIPQYVVGHQKRVDGIKQYIRDHNLPLGLCGASYDGVGVNDVILSARVSVDE
VVG
>AaPO
-MTAILGAGISGLSAGYYLLKK-GLPPTVIYEASNRIGGWIRTDRFNEGFVFEAGPRTIRPKGPAASNTLDLIEEVGLADEVYPIYSNHVAARN
RMIYAKGKLNMLPSDLSGVLKTVP-PFTKPLFFAGFKDIFAGRSKVEDESMYSFVERRFGKEIADYAVSSMLCGICAGDAKQISVKFLMKSLFE
MEQKHGGVVKGMLKEAKGKKDGAKELAKRAKSENWSIYSIHGGLQTLPDRLGTVLHK-NGVGIETDVKFEEIMFDGG-KVQLKVDGQDRVVDHL
ISSIPSYKLAKRVSSQHPELSKELAAIPYVDVAVINLCYKTDLQKHKGFGFLVPPIENLPILGVIFDSCCFDMDDN-----TVLTVMMGGAWFE
KWFGSNP--DEELLLDVALKNVNRILDIQQAPDAYKVNLLRKCIPQYVVGHHKRVEAIRAYIRDHKLPLGLCGASYDGVGVNDVILSARKCVEE
QVK
>AdPO

---------------------------MSKSMLPFILREPFVGRKQDSDESVYEFFRRRLSKEVAEYMVDPFCRGIFAGDARVLSLRSCFPPLYE
LENKHGSIIKGVLKSSLKPASPDSSLGEKAKKERWSTWSLRGGLQTLSDKLLEELQR-KGVNVKLESPCTSVKFDSCGKLTVSSPTEQIRVDHV
ISALPAHTLATVLAKDWQQLATDLSNIRTVTVAVVNLEYEGNVVPMDGFGFLVPSSDAVQILGIIFDSCAFPENDRLGAKTTRLTVMMGGHWFD
SLFGHPDSVDPQHLQRVAIETAEKTLGIRAKPTTCLTNVLRDCITQYTIGHSERLERISQFIKRNKLPLTLIGASYNGVSVNDCIYKTQLAVDD
IFE
>LaPO
RTVVVLGGGISGLAASYHLSRAPDPPKVVVVEGSKRLGGWIRSVRGPDGAIFELGPRGIRPAGVLGARTLLLVSELGLDSEVLPVRGDHPAAQN
RFLYVGGALHPLPTGLRGLFRPSP-PFSKPLFWAGLRELTKPRGKEPDESVHSFAQRRLGPEVASLAMDSLCRGVFAGNSRELSIRSCFPSLFQ
AEQTHRSILLGLLLGAGRSPQPDSALVRQARAERWSQWSLRGGLEMLPQALDTHLTS-RGVSVLRGQPVCGLSLQAEGRWKVSLGDSSLEADHV
ISAVPASVLSELLPAEAAPLARALRTITAVSVAVVNLQYQGARLPVQGFGHLVPSSEDPGVLGIVYDSVAFPEQDG-RSPGLRVTVMLGGSWLQ
MQEASGCVLSQELFQQEAEKAAAIQLGLKEPPSHCLVHLHKNCIPQYTLGHWQKLESATQFLATHRLPLTLAGASYEGIAVNDCIESGRQAAAR
ALG
>OcPO
RTVVVLGGGISGLAASYHLSRAPRPPKVVLVEGSERLGGWIRSVRGPGGAVFELGPRGIRPAGALGARTLLMVSELGLDSEVLPVRGDHPAAQN
RFLYVGGALHALPTGFRGLLRPSP-PFSKPLFWAGLRELTKPRGKEPDETVHSFVQRRLGPEVASLAMDSLCRGVFAGNSRELSIRSCFPSLFQ
AEQTHRSILLGLLLGAGQSPPPDSALIRQARAERWSQWSLRGGLEMLPQALDTHLTS-RGVQVLRGQPVCRLSLQAGGRWKVSLGDSSLEADHI
ISAIPAPALSKLLPAEAAPLARVLSTITAVSVAVVNLQYRGAHLPVQGFGHLVPSSEDPGVLGIVYDSVAFPEQDG-SPPGLRVTVMLGGSWLQ
TLEASGRVLPQELFQQQAQEAAATQLGLKEPPSHCLVHLLKNCIPQYTVGHWQKLESAMQFLAAQRLPLTLAGASYEGVAVNDCIESGRQAAAS
VLG
>HmPO

FRIIILGSGISGLSAAETVIKYSQVPKVTLVECEPHVGGWLQSKVYEDGSVFEHGPRSARSIGDTALQALNIMSDIGIGSKIIGIPRNKITST-
RYIYANGKMNELPSSISSMLFRQD-PFSRSIISIIAKELFVEKNVTEDESIYSFFCRRFNKEIADYLATSLCRGIFGGDANVLSMKSCFRQVFE
FEKKYGSVTRGMFFSKSGNAESLSDLALKAKREKWTSWTVQNGMQALPKYWADSLRL-NGVQILLDTKCEKLSFSNE-KVFCTTNSCVLEADAV
ISSLPVCSLKSLLTEANSTLSALLGSINFATMAVVNLEYVNEKVPFNGFGFLVPSNQPLNILGMTFDSVVLPHPTQHNS--AILTVMSGGAWFE
EIFGNPNSCDENMIIESSVKNVRDILGFRSMPRRVLCKIQKNCIPQYYIGHDERVEQIKNFLKASDLPLLITGTSWGGVGINDAIINSKNDTLR
WLA
>NvPO
SRFTVLGGGISGLSAAYYLAKHHSKPDITVLEASGRFGGWMHSKRSPEGAVFEQGPRSLRPAGPPGWTTLDLVSELGLESNIIPVNTSHPGATN
RFIYSRGKVHKLPNSIISILKKQS-LFSQSLLPTILKEPFKEKRISNDESVYSFFSRRLNREVAEYIGDPVCRGIFAGDARALSLRAVFPAVYE
MEKNYGSVVKGALLGKSVKPGPDASLVQRSINEKWSIWSLDGGLQTFADKLHNSLNQ-MGVKFIVGKPCFAIDFTDDYKAILQIAEEKLETDHL
ISSLSADRLASILPSSHSNLVDALSEIKPVSVGLVNLEYNGNIIKDEGFGVLLPSSEATKVLGIIYDSCLFPQHDRKDGGHTRLTVMMGGHWFE
EQFGHPDIVDTSVLTDTAVDAVAKIMGVSKDPIKTLTSVQTNCISQYHIGHTSRVNRIFNYVRDNGLPLSLVGSSYKGVGVNDCVYNAKVMVEE
LVA
>DaPO
MTTAVLGGGLSGLSAGYYLLRRFGKP-LTIYEAAPRVGGWVRSEFRKDGFIFESGPRTIRPVGVPGANTLELVEE--LQLDIKPITRSHVAARN
RMLYAKGQLCMLPNSPKGLFGVMP-PFTKPLYKALLRDLVTQSTPAEDESIYSFAERRFGKEIADYAISPMICGICAGDAREISVRFLMEGLFE
KEQKYGGVLKGSLIARGKVDEGKEGLYSQAVQEKWAMYGLTGGLEILPRTMRKYLGE-RDVNVQLSNECKNLTFGNS-GVRMTVKDADVPVDHI
VCSLPAHKLAPLVKVQHPSLSAQLLDIPYVDVVVVNMQFQGQLLKQDGFGLLVPPVEKLPILGVIFDSCCFDMGGN-----TVLTVMMGGHWFD
KWFGDRP--SQKQILDLATHHVQEILGIRDDPKFSRVHTLHKCIPQYTVGHKRRVDNIRKYLKTYKLPLSLCGAAYDGVGINDVILSARRQVEA
LQL
>DpPO
MKTAVLGGGLSGLSAGYYLLRRFGNP-LTIYEASPRVGGWIRTEARRDDFIFETGPRTIRPVGIPGANTLEMMEE--LQLPLKPIPRSHVAARN
RMLYAKGQLCMLPNSPRGLFGVKP-PFTQPLYKALLHDLSTGRTLAEDESIYSFAERRFGKEIADYAISPMICGICAGDAKQISVRFLMEGLFE
KEQKYGGVLKGILYARDNVDEKKPGLYAQALQEKWAMYSIEGGLEVLPRAMRKYLDQ-HNVNVQLSNACQTLTFSKS-GVRMSLRNADVPVDHV
VSSLPAYKLAPLLKPQHPSLCEQLLEIPYVNVVVVNIQYNGNQLKQDGFGLLVPPVEKLPILGVIFDSCCFDMNGN-----TVLTVMMGGHWFD
QWFGHQP--SQKQILDIAKKYVREILDIQEEPKFSRVHILPKCIPQYTVGHKERVKRIRKYLKDYKLPLSLCGAAFDGVGINDVILSARRQVDA
IP-
>AmPO
-MTAILGAGISGLSAAYYALGNTRMAPLVILEASNRVGGWIRSIKQSDGTIFETGPRVMRAN---TYNVLNLIEELGLSSKIIPIKVNHPAAKN
RYIYADNVLHCLPNNLKGIITKNT-LLNRSLSSILWNDFKAKKILTDDESISSFVERRFGKDVSEKMIAPILCGICGGDIHKLSAKSFMTNLFE
IEQKYGSVFMGFIQQKNKIENDTDTLAQRAKREAWSTWGLEGGFEQLPQTLAENIMK-RGVNIKIKHKCEKIIFNKD-CVELIINGKVEKYSSI
ISSLSAKSLANLIQEQHPKLSKELHSIPTVTMIVVNFQFSKNVLPIEAFGVLIPPKEEIPILGIIFDSCALPQNSKM----TVLTVMMGGAWFE
KYFGKCS--SEEHLKMVAIKYVNKLLCINEDPKICNVSILKDCIPQYVIGHAQRLTRIHDYISAHKIPLGLCGSSYHGVXVSHVILSAKEAVSS
IIN

#FECH#
>HsFC
RKPKTGILMLNMGGPETLGDVHDFLLRLFLDRDLMTLPIQNKLAPFIAKRRTPKIQEQYRRIGGGSPIKIWTSKQGEGMVKLLDELSPNTAPHK
YYIGFRYVHPLTEEAIEEMERDGLERAIAFTQYPQYSCSTTGSSLNAIYRYYNQVGRKPTMKWSTIDRWPTHHLLIQCFADHILKELDHFPLEK
RSEVVILFSAHSLPMSVVNRGDPYPQEVSATVQKVMERLEYCNPYRLVWQSKVGPMPWLGPQTDESIKGLCERGRKNILLVPIAFTSDHIETLY
ELDIEYSQVLAKECGVENIRRAESLNGNPLFSKALADLVHSHIQSNELCSKQLTLSCPLCVNPVCRETKSFFTSQQL
>ClfFC
RKPKTGILMLNMGGPETVGDVHDFLLRLFLDRDLMTLPIQDKLAPIIAKRRTPKIQEQYRRIGGGSPIKMWTSKQGEGMVKLLDELSPHTAPHK
YYIGFRYVHPLTEEAIEDMERDGIERAIAFTQYPQYSCSTTGSSLNAIYRYYNKVGKKPAMKWSTIDRWPTHPLLIQCFADHIVKELDHFPLEK
RSEVVILFSAHSLPMSVVNRGDPYPQEVGATVQRVMDKLGYSNPYRLVWQSKVGPMPWLGPQTDETIKGLCERGRKNILLVPIAFTSDHIETLY
ELDIEYSQVLANECGVENIRRAESLNGNPLFSKALADLVLSHIQSNELCSKQLTLSCPLCVNPVCRETKSFFTSQQL
>BtFC
RKPRTGILMLNMGGPETVEEVQDFLQRLFLDQDLMTLPVQDKLGPFIAKRRTPKIQEQYRRIGGGSPIKMWTSKQGEGMVKLLDELSPHTAPHK
YYIGFRYVHPLTEEAIEEMERDGLERAVAFTQYPQYSCSTTGSSLNAIYRYYNEVGRKPTMKWSTIDRWPTHPLLIQCFADHILKELDHFPPEK
RREVVILFSAHSLPMSVVNRGDPYPQEVGATVQRVMDKLGYSNPYRLVWQSKVGPMPWLGPQTDEAIKGLCKRGRKNILLVPIAFTSDHIETLY
ELDIEYSQVLASECGLENIRRAESLNGNPLFSKALADLVHSHLQSKERCSTQLTLSCPLCVNPTCRETKSFFTSQQL
>MmFC
RKPKTGILMLNMGGPETLGEVQDFLQRLFLDRDLMTLPIQNKLAPFIAKRRTPKIQEQYRRIGGGSPIKMWTSKQGEGMVKLLDELSPATAPHK
YYIGFRYVHPLTEEAIEEMERDGLERAIAFTQYPQYSCSTTGSSLNAIYRYYNEVGQKPTMKWSTIDRWPTHPLLIQCFADHILKELNHFPEEK
RSEVVILFSAHSLPMSVVNRGDPYPQEVGATVHKVMEKLGYPNPYRLVWQSKVGPVPWLGPQTDEAIKGLCERGRKNILLVPIAFTSDHIETLY
ELDIEYSQVLAQKCGAENIRRAESLNGNPLFSKALADLVHSHIQSNKLCSTQLSLNCPLCVNPVCRKTKSFFTSQQL
>GgFC

RKPKTGILMLNMGGPERLDDVHDFLLRLFLDRDLMTLPAQNKLAPFIAKRRTPRIQEQYSRIGGGSPIKKWTAVQGEGMVKLLDSMSPQTAPHK
YYIGFRYVHPLTEEAIEEMEDDGIERAIAFTQYPQYSCSTTGSSLNAIYRYYNKKGKKPKMKWSIIDRWPTHPLLIQCFADHIQKELDLFPPDK
RKDVVILFSAHSLPMSVVNRGDPYPQEVGATVQRVMEKLNHSNPYRLVWQSKVGPMPWLVPQTDETIKGLCQRGKKNMLLVPIAFTSDHIETLY
ELDIEYAQVLANECGVENIRRAESLNGNPLFSKALADLVCSHIQSNEICSKQLTLCCPLCVNPVCRETKAFFTNQQL
>DrFC
RKPKTGILMLNMGGPEKLEDVHDFLLRLFMDTDFMQLPVQNKLGPFIAKRRTPKIQEQYSKIGGGSPIKAWTTMQGEGMVKLLDEMCPDTAPHK
FYIGFRYVHPLTEEAIELMEKDGVERAVAFTQYPQYSCSTTGSSLNAIYRYYSNRADRPKMRWSVIDRWPTHPLLIECFAEHVRNELDKFPVEK
RDDVVILFSAHSLPLSVVNRGDPYPQEVGATVQRVMDRLGHCNPYRLVWQSKVGPMAWLGPQTDEVIKGLCQRGKRNLLLVPIAFTSDHIETLH
ELDIEYSQVLGEEVGVENIRRAESLNGNPLFFRALADLVQSHLQSNESCSRQLTLRCPLCVNPTCAQTKAFFSSQKL
>DmFC
QKPKTAILMLNMGGPTHTDQVHDYLLRIMTDRDMIQLPVQSRLGPWIAQRRTPEVQKKYKEIGGGSPILKWTELQGQLMCEQLDRISPETAPHK
HYVGFRYVNPLTENTLAEIEKDKPERVVLFSQYPQYSCATSGSSFNSIFTHYRSNNLPSDIKWSIIDRWGTHPLLIKTFAQRIRDELAKFVETK
RNDVVILFTAHSLPLKAVNRGDAYPSEIGASVHMVMQELGQTNPYSLAWQSKVGPLPWLAPATDDAIKGYVKQGLKNFILVPIAFVNEHIETLH
ELDIEYCDELAKEVGVEEIRRAATPNDHPLFIDALTNVVADHLKSQQAVNPKFLMRCPMCSNPKCRESKSWYRQLCS
>XlFC
RKPKTGILMLNMGGPETLDDVHGFLLRLFLDKDLMTLPAQSKLAPFIAKRRTPKIQEQYSKIGGGSPIKKWTEQQGEGMVKLLDELSPATAPHK
YYIGFRYVRPLTEAAIEEMERDGVERAIAFTQYPQYSCSTTGSSLNAIYRYYNAKGTQPKMKWSVIDRWPTHPLLIQCFADHIQKELNMFPADK
RGEVVILFSAHSLPMSVVNRGDPYPQEVGATVQKVMERLGFSNPYRLVWQSKVGPMAWLGPQTDESIKGLCQRGKKNILLVPIAFTSDHIETLY
ELDIEYAQVLAKECGVENIRRSESLNGNPLFSKALADLVLSHMKSSEICSKQLSLRCPMCVNPVCGEAKSFFTKQQQ
>XtFC
RKPKTGILMLNMGGPETVDDVHDFLLRLFLDKDLMTLPAQSKLAPFIAKRRTPKIQKQYSKIGGGSPIRKWTEQQGDGMVKLLDELSPATAPHK
YYVGFRYVNPLTEAAIEEMERDGVERAIAFTQYPQYSCSTTGSSLNAIYRYYNTKGAQPKMKWSVIDRWPTHPLLIQCFADHIQKELNMFPADK
RGDVVILFSAHSLPMSVVNRGDPYPQEVGATVQKVMERLGYSNPYRLVWQSKVGPMPWLGPHTDESIKGLCQRGKKNILLVPIAFTSDHIETLY
ELDIEYAQVLANECGVENIRRSESLNGNPLFSKALADLVLSHMKSNEICSKQLTLRCPMCVNPVCGEAKSFFTKQQQ
>BfFC
RKPKTGIMMMNLGGPETLEDVQSFLLRLFSDKDLIPLPAQSFLAPRIAKRRTPKIQEQYSRIGGGSPIKMWTTKQGEGMIKILDQISPQTAPHK
FYIGFRYADPLTEDTIEQMEKDGIERAVAFTQYPQYSCSTTGSSLNAIYRYYNKRGERSQMKWTVIDRWPTHPMLVECFAQNIQKELAKFPADV
RDDVVILFSAHSLPMSVCEQRRPVPPGGWCDCTVVDGIVVFSPGYRLDWQSKVGPLPWLGPQTDEVIQGLANNGKKNLLLVPIAFTSDHIETLH
ELDIEYAATLAAECGVENVRRAESLNDNPLFIEGLADLVKTHLDKGTSCTRQLMLRCPMCVNPACGPAKQFFHDQGQ
>CiFC
VKPKTGIVLLNMGGPEKWENVYDFLFRLFSDPDLIPLPARSRLAPFIAKRRTPKIEEQYKKIGGGSPITKWTNHQGTEMVKILDKISPETAPHK
HYIGFRYVPTLTEDALDQMRDDGVERAVAFTQYPQYSCSTTGSSLNAIFRHYKRNNDVSAMKWSVIDRWPTHSGLVEAFADRTAAALEKFPKDV
RNKVVILFSAHSLPMSVVNRGDPYPAEVAATVSRVMERLDNSNPYRLVWQSKVGPMPWLGAQTDEAIKGLCRNGFKNLLLVPIAFTSDHIETLF
ELDEEYIGELAMEAGVENIHRAESLNDSPIFIQAMADVVKSHLNSDAVSTSQLPLRCPLCTNEACVGMRNFFLKNSV
>SpFC
QKAKTGIMMLNMGGPENTDEVHDFLLRLFMDKDIIPLPAQSKLAPIIARRRTPKIQAQYKKIGGGSPIKKWTKLQGEGLVKLLDKLSPETAPHK
YYIGFRYVNPLTEDTIDQMERDGIERAIAFTQYPQYSCSTTGSSLNAIYRHYAKNPENSTMKWSVIDRWPVNAGLVEAFSQHVQAELEKFPADV
KDDVVILFSAHSLPMSVVNRGDPYPAEVGATVQRVMERLNFSHPYRLVWQSK---

>MmuFC
RKPKTGILMLNMGGPETLGDVHDFLLRLFLDRDLMTLPIQNKLAPFIAKRRTPKIQEQYRRIGGGSPIKIWTSKQGEGMVKLLDELSPNTAPHK
YYIGFRYVHPLTEEAIEEMERDGLERAIAFTQYPQYSCSTTGSSLNAIYRYYNQVGRKPTMKWSTIDRWPTHHLLIQCFADHILKELDHFPLEK
RSEVVILFSAHSLPMSVVNRGDPYPQEVSATVQNVMERLGYCNPYRLVWQSKVGPMPWLGPQTDESIKGLCERGRKNILLVPIAFTSDHIETLY
ELDIEYSQVLAKECGVENIRRAESLNGNPLFSKALADLVHSHIQSNELCSKQLTLSCPLCVNPVCRETKSFFTSQQL
>TgFC
--------MLNMGGPERLDDVHDFLLRLFLDRDLMTLPVQNKLAPLIAKRRTPKIQEQYSRIGGG-PIKKWTAVQGEGMVKLLDSMSPLTAPHK
YYIGFRYVHPLTEEAIEEMERDGIQRAIAFTQYPQYSCSTTGSSLNAIYRYYNQKGEKPKMKWSIIDRWPTHPLLIQCFADHIQKELNLFPADK
RKEVVILFSAHSLPMSVVNRGDPYPQEVGATVQRVMEKLNYSNPYRLVWQSKVGPMPWLGPQTDETIKGLCQQGKKNMLLVPIAFTSDHIETLY
ELDIEYAQVLANECGVENIRRAESLNGNPLFSKALADLVCSHLQSNEVCSRQLTLCCPLCVNPVCREAKAFFSSQPL
>AlFC
--------MLNMGGPETLGDVHDFLLRLFLDRDLMTLPIQNKLAPFIAKRRTPKIQEQYSQIGGGSPIKKWTATQGEGMVKLLDEMSPHSAPHK
YYIGFRYVHPLTEEAIEEMEKDGIQRAIAFTQYPQYSCSTTGSSLNAIYRYYNAKGEKPKMKWSTIDRWPTHPLLIQCFADHIEKELALFPPDK
RKDVVILFSAHSLPMSVVNRGDPYPQEVGATVQRVMEKLKYSNPYRLVWQSKVGPMPWLGPQTDETIKGLCQRGKKNILLVPIAFTSDHIETLY
ELDIEYAQVLANECGVENIRRAESLNGNPLFAKALAELVFSHLQSNELCSRQLTLSCPLCVNPTCRKTKSFFTSQEL
>OlFC
RKPKTGILMLNMGGPEKLEDVHDFLLRLFLDTDLMKLPVQSKLGPFIAKRRTPKIQEQYSKIGGGSPIKRWTSMQGEGMVKLLDEMSPDTAPHK
FYIGFRYVNPLTEEAIEEMEKDGVERAVAFTQYPQYSCSTTGSSLNAIYRYYSNKGGRPNMRWSVIDRWPTHPLLVECFAEHVLNELQKFPEDK

RDDVVILFSAHSLPMAVVNRGDPYPQEVGATVQRVMERLGHCNPYRLVWQSKVGPMAWLGPQTDEVIKGLCERGKKNLLLVPIAFTSDHIETLH
ELDIEYAQVLGEECGVENIRRAESLNGNPLFMKALADLVQSHLKSNEPCSRQLTLRCPLCTNPTCGETKAFFAGQKL
>GaFC
RKPKTGILMLNMGGPEKLEDVHDFLLRLFLDTDLMKLPVLNTLGPLIAKRRTPKIQEQYSKIGGGSPIKHWTSMQGEGMVKLLDEMSPETAPHK
FYIGFRYVQPLTEEAIEQMEKDGVERAVAFTQYPQYSCSTTGSSLNAIYRYYNNRGARPKMRWSVIDRWPTHPLLVECFADHVRNELLKFPDDK
RDDVVLLFSAHSLPMAVVNRGDPYPQEVGATVQRVMETLGHCNPYRLVWQSRVGPMQWLGPQTDDVIKGLCERGKKNILLVPIAFTSDHIETLH
ELDIEYGQVLGEECGVENIRRAESLNGNPLFMKALADLVQSHLKSNQPCSRQLTLRCPLCTNPTCAETKAFFSSQTL
>TnFC
RKPKTGILMLNMGGPEKLEDVHDFLLRLFMDTDLMQLPVQNKLGPFIAKRRTPKIQEQYSKIGGGSPIRRWTSMQGEGMVKLLDEMSPQTAPHK
FYIGFRYVHPLTENAIEEMERDGVERAVAFTQYPQYSCSTTGSSLNAIYRYYSNRGERPKMSWSVIDRWPTHPLLVECFAEHIQNELLQFPEEK
RDDVVILFSAHSLPMAVVNRGDPYPQEVGATVQRVMERLGHCNPYRLVWQSKVGPMAWLGPQTDEVIKG-CERGKKNILLVPIAFTSDHIETLH
ELDIEYGQVLGEECGVENIRRAASLNGNPLFMKALADLVQSHLKSNMVCSRQLTLRCPLCTNPTCGETKAFFASQKL
>CsFC
MKPKTGILLLNMGGPEKWENVYDFLFKLFSDPDLIPLPARSRLAPFIAKRRTPKIEEQYQKIGGGSPITKWTNHQGLEMVKLLDKLSPETAPHK
HYIGFRYVPTFTEDALDQMRDDGVKRAVAFTQYPQYSCSTTGSSLNAIFRHYKKNHDALNIKWSVIDRWSTHIGLIEAFAVRTLAALEKFPEEI
RSKVVILFSAHSLPMSVVNRGDPYPAEVAATVSRVMERIGYSNPYRLVWQSKVGPMPWLGAQTDDAIKGLCRNGFKNLLLVPIAFTSDHIETLY
ELDEEYIGELSKEAGVENIHRAESLNGSPIFIQAMADVVKSHLDSGFISTPQLPLRCPMCTNEACVGMRNFFLNGAI
>DsFC
QKPKTAILMLNMGGPTHTDQVHDYLLRIMTDRDMIQLPVQSRLGPWIAQRRTPEVQKKYKEIGGGSPILKWTELQGQLMCEQLDRISPETAPHK
HYVGFRYVNPLTENTLAEIENDKPERVVLFSQYPQYSCATSGSSFNSIFTHYRSNDLPSDIKWSIIDRWGTHPLLIKTFAQRIRDELAKFVETK
RNDVVILFTAHSLPLKAVNRGDAYPSEIGASVHMVMQELGQTNPYSLAWQSKVGPLPWLAPATDDAIKGYVKQGLKNFILVPIAFVNEHIETLH
ELDIEYCDELAKEVGVEEIRRAAAPNDHPLFIDALTNVVADHLKSQQAVNPKFLMRCPMCSNPKCRESKSWYRQLCS
>AmeFC
---MTCIVQTNLD------------------------------------QRRV-----------------QWTNKQGKLLCEKLDKISPKTAPHK
HYVAFRYANPLTENTLQKIEEDGVEHTIIFSQYPQYCCATSGSSFIEIYKYYKNRQLPSNMKWSVIDRWATHPLFIETITERIKEELVLFPDDI
RSDVIILFSAHSLPLKAVSRGDAYASEVAGTVALVMEKLQYCNPYKLVWQSKVGPVSWLEPFTDDAIKAYVKQGKKHFILVPVAFVNEHIETLH
ELDIEYCKELAEELGIDKIRRTAAPNDHPTFINALADIVVSHLKSNNSLSPMFLTRCPHCVSGNCAESKKWYAQICR
>AgFC
TKPRTAVVMLNMGGPQNTDQVHDYLHRIMTDRDMIQLPVQSKLGPWIAKRRTPEVQKKYSEIGGGSPILKWTNVQGELMCKQLDKLSPETAPHK
HYVAFRYVNPLTEDTFREVERDQPERVVLFSQYPQYSCATSGSSFNAIYTHFKENNGLAQARWSVIDRWGTHPLLAKTFADNIRKELDKFPAEK
RKDVVLLFSAHSLPLRAVSRGDAYPSEVGATVQNVMQELGWSQPYCLVWQSKVGPLPWLEPFTDDAIKGYVKQGKKNFILVPIAFVNEHIETLH
ELDIEYCEELAHEVGAEKIGRAAAPNDHPLFIEALTDVVRHHLANGSTVGPKFLLRCPACVNQKCSVSKQWFNQLCN
>CqFC
SKPRTAIVMLNMGGPQNTDQVHDYLLRIMTDRDMIQLPVQSKLGPWIAKRRTPEVQKKYAEIGGGSPIFKWTNLQGELLCKQLDKVSPETAPHK
HYVAFRYVTPLTEDTLKQVEADQPERVVLFSQYPQYSCATSGSSFNAISTHYKTNNGLSNAKWSIIDRWGTHPLLARTFADNIRKELEKFPAEK
RKDVIILFSAHSLPLRAVNRGDAYPSEVGATVQNVMGELNYCNPYCLVWQSKVGPLPWLEPFTENAIKGYVKQGKKNFILVPIAFVNEHIETLH
ELDIEYCQELAHEVGAEKIGRAAAPNDHPLFIEAMTDVVSSHLKNGNRVNPKFLLRCPSCINEKCGTSKQWYKEMCN
>IsFC
HDSNSAVPRRRLDGDSSTEDVELFLKRVFTDNDIMSMPFQSILGPLIARRRAPKLAEKYKELEGSSTLLQWTELQGRQITNTLDSISPKTGPHK
YYIGFRYTDPLTEDSLEQIEKDGVERVVAFSQYPQYSCCTSGSSLNAIFRFYNQRKRKSAAQWSFIDRWPIHDAITKGYASIIKEELKKFPEEV
RDQVVILFSAHSLPMKVVDKGDPYPTEVAATVVGVMNELNNSHPYRLVWQSKVGPLPWLKPETEASMRALARKGHRHQLLVPVAFVNEHIETLH
EMDLELGRDVAPKIGLLNFRRAPALNDHPAFIQGLADLVKEHLESGQKCSPQLLLTCPMCDKRICKDMREWVASLP-
>AaFC
SKPRTAIVMLNMGGPQTTDQVHDYLLRIMTDRDMIQLPVQSKLGPWIAKRRTPEVQKKYAEIGGGSPIFKWTNIQGELLCKQLDKVSPETAPHK
HYVAFRYVTPLTEDTLKQVEQDQPERVVLFSQYPQYSCATSGSSFNAIYTHYKSNNGLANAKWSVIDRWGTHPLLAKTFADKIRKELEKFPAEK
RKDVILLFSAHSLPLKAVNRGDAYPSEVGATVQNVMEHLKYCNPYCLVWQSKVGPLPWLEPFTEDAIKGYVKQGKKNFILVPIAFVNEHIETLH
ELDIEYCQELAHEVGAEKIGRAAAPNDHPLFIDALTDVVSSHLKDANAVNPKFLLRCPSCVNAKCSSSKQWYREMCN
>LaFC
RKPKTGILMLNMGGPETLGEVHDFLLRLFLDRDLMTLPLQKYLAHIIAKRRTPKIQDQYRRIGGGSPIKMWTSKQGEGMVKLLDELSPHTAPHK
YYIGFRYVHPLTEEAIEEMERDGLERAIAFTQYPQYSCSTTGSSLNAIYRYYNGVGKKPTMKWSTIDRWPTHPLLIQCFADHILKELDHFPLEK
RSEVVILFSAHSLPMSVVNRGDPYPQEVGATVQRVMDKLGYSNPYRLVWQSKVGPMPWLGPQTKEAIKGLCERGRKNILLVPIAFTSDHIETLF
ELDIEYSQVLANECGVEKIRRAESLNGNPLFSKALADLVLSHIQANERCSKQLTLSCPLCANPVCREMKSFFTTQQL
>OcFC
RKPKTGILMLNMGGPETLGEVHDFLLRLFLDRDLMTLPVQSKLGPFIAKRRTSKIQEQYRRIGGGSPIKMWTSKQGEGMVKLLDELSPHTAPHK
YYIGFRYVHPLTEEAIEEMERDGLERAIAFTQYPQYSCSTTGSSLNAIYRYYNGAGKKPAMRWSTIDRWPTHPLLIQCFADHILKELDRFPPEK
RSEVVILFSAHSLPMSVVNRGDPYPQEVGATVHRVMERLGYSNPYRLVWQSKVGPMPWLGPQTDETIRGLCERGRKNILLVPIAFTSDHIETLY
ELDIEYSQVLAKECGVENIRRAESLNGNPLFSKALADLVHAHIQSDELCSKQWTLGCPLCVNPICRETKSFFTNQRL

>HmFC

QAPQTGIMMLNMGGPSTLDEVGSFLSRLFHDKDLIPLPAQKQLAPLLAARRTPKIVEQYRKIGGGSPIRKWTEIQGQALIKLLDKMSPETAPHK
FYIGFRYADPLTEHSLSEMERDGIKRAIAFTQYPQYSCSTTGSSLNAIYRYYNKLNVPSSIEWSVIDRWPTHHGLIQAFAQTIVQELEKFPSNV
KDDVVILFSAHSLPMSVVNRGDPYPQEVSATVQSVMEMLKFRNQYRLCWQSKVGPLPWLGPQTEDSIKGLVKNGRKNILLVPIAFTSDHIETLH
ELDLEYADELAKEVGVENIRRSAAMNDNSIFVQALADLVRKHIYSGETCSKQLLLRCPMCVNVACGPMRDFFASRGL

>NvFC

LAPKTGILLLNLGGPEKQEDVHGFLLRLFSDKDLIPLPAQKQLAAWIAKRRTPKIKEQYQKIGGGSPIKMWTEKQGQGMVELLDQLSPETAPHK
FYVGFRYATPLTEDAIEQMERDGIERAIAFTQYPQYSCSTTGSSINAIYKYYKQRQGNSSLKWSAIDRWPTHPGLIKAFADNVKVELSKFPEEV
QKDVVILFSAHSLPMKVVDRGDPYPQEVAATVQRVMEALDFSHSYRLVWQSKVGPLPWLGPQTEDAIKGLAKNNKKNLLLVPIAFTSDHIETLH
ELDIEYAQELAHEVGVENIRRAESLNDNPIFIQAMADIVHKHLQSGETCSRQLPLRCPMCVNATCGPAKEFFANQTL

>DaFC

QQPKTAILMLNMGGPQNTDQVHDYLLRIMTDRDMIQLPVQSRLGPWIAQRRTPEVQKKYKEIGGGSPILKWTELQGQLMCERLDKISPETAPHK
HYVGFRYVNPLTENTLAEIEKDKPERVVLFSQYPQYSCATSGSSFNSIFTHYKKNNTPSNIKWSIIDRWGTHPLLVKTFAQRIRDELAKFDETK
RNDVVILFTAHSLPLKAVNRGDAYPSEIGASVHMVMQELGQTNPYSLAWQSKVGPLPWLAPATDDAIKGYVKQGQKNFILVPIAFVNEHIETLH
ELDIEYCDELAKEVGVEEIRRAAAPNDHPLFINALSDIVADHLKSQQSVNPKFLMRCPMCTNPKCRESKKWYQQLCS
