# Supplementary material for: Molecular Evolution of Multiple-Level Control of Heme Biosynthesis Pathway in Animal Kingdom
Source: PLoS One. 2014 Jan 28;9(1):e86718. doi: 10.1371/journal.pone.0086718 (PMC3904948; doi:10.1371/journal.pone.0086718)
Supplement: Table S1 — Model test (M1a) for selection of genes in heme biosynthesis pathway. (PDF) [file pone.0086718.s004.pdf]

Table S1. Model test for selection of genes in heme biosynthesis pathway.

Gene	M0		M1a			
	LnL[a]	ω	LnL[a]		2ΔL[b]	*p* value[c]
ALAS	-29312.4	0.05356	-29058.8	p0 = 0.91614, ω0 =0.04740	507.094818	<0.001
PBGS	-14134.9	0.06398	-14105.2	p0 = 0.96190, ω0 =0.06348	59.378706	<0.001
PBGD	-9517.86	0.04448	-9490.76	p0 = 0.96675, ω0 = 0.04117	54.207694	<0.001
UROS	-9311.62	0.12652	-9265.06	p0 = 0.93205, ω0 = 0.11646	93.10792	<0.001
UROD	-11531.2	0.06194	-11501.9	p0 = 0.91111, ω0 = 0.07122	58.616248	<0.001
CPO	-7212.75	0.04804	-7110.82	p0 =0.92322 , ω0 = 0.04145	203.848868	<0.001
PPO	-17836.4	0.10779	-17669.6	p0 = 0.78652, ω0 = 0.10512	333.43545	<0.001
FECH	-8833.94	0.04141	-8778.1	p0 =0.95812 , ω0 = 0.03364	111.691194	<0.001

[a]The log likelihood (LnL) is shown for each model. p0 is the proportion of codons with ω0 < 1.

[b]Twice the difference between the log likelihood of M0 and M1a.

[c]*p* value p(χ2) of the likelihood ratio test. We did not find the any significance for M2a model.
