# Supplementary material for: Molecular Evolution of Multiple-Level Control of Heme Biosynthesis Pathway in Animal Kingdom
Source: PLoS One. 2014 Jan 28;9(1):e86718. doi: 10.1371/journal.pone.0086718 (PMC3904948; doi:10.1371/journal.pone.0086718)
Supplement: Table S2 — Length of evolutionarily conserved DNase-hypersensitive sites in intron sequences. (PDF) [file pone.0086718.s005.pdf]

Table S2. Length of evolutionarily conserved Dnase-hypersensitive sites in intron sequences (bps)

Intron[a]	ALAS1	ALAS2	PBGS	PBGD	UROS	UROD	CPO	PPO	FECH
1	0	63	66	139	44	43	0	0	54
2	13	0	0	3	0	0	0	3	165
3	51	0	0	0	0	0	0	0	0
4	22	0	0	0	10	0	0	0	8
5	6	0	0	6	20	0	5	0	32
6	8	0	0	0	3	0	0	0	227
7	5	0	0	0	2	20	-	0	0
8	0	54	0	7	0	0	-	0	7
9	0	0	0	3	0	0	-	0	0
10	21	0	0	3	-	-	-	0	0
11	3	0	0	17	-	-	-	0	0
12	-	-	-	7	-	-	-	0	-
13	-	-	-	3	-	-	-	0	-

[a]Intron ID.
