# Supplementary material for: Molecular Evolution of Multiple-Level Control of Heme Biosynthesis Pathway in Animal Kingdom
Source: PLoS One. 2014 Jan 28;9(1):e86718. doi: 10.1371/journal.pone.0086718 (PMC3904948; doi:10.1371/journal.pone.0086718)
Supplement: Table S3 — Potential IRE in eight genes of heme biosynthesis pathway. (PDF) [file pone.0086718.s006.pdf]

Table S3. Potential IRE in ALAS

Species	common name	Distance to transcription start site (bases)	Distance to start codon (bases)[a]	Location[b]	Quality[d]
Gasterosteus aculeatus AS1b	Stickleback AS1b	-12	-183	5UTR[c]	High
Oryzias latipes AS1	Medaka AS1	47	-153	5UTR	High
Takifugu rubripes AS1a	Fugu AS1a	11	-163	5UTR	High
Homo sapiens AS2	Human AS2	108	-30	5UTR	High
Macaca mulatta AS2	Rhesus monkey AS2	12	-379	5UTR	High
Bos taurus AS2	Cattle AS2	22	-30	5UTR	High
Canis lupus familiaris AS2	Dog AS2	29	-30	5UTR	High
Mus musculus AS2	Mouse AS2	96	-30	5UTR	High
Xenopus (Silurana) tropicalis AS2	Western clawed frog AS2	4	-36	5UTR	High
Danio rerio AS2	Zebrafish AS2	75	-26	5UTR	High
Gasterosteus aculeatus AS2	Stickleback AS2	496	-213	5UTR	High
Gasterosteus aculeatus AS2	Stickleback AS2	673	-36	5UTR	High
Oryzias latipes AS2	Medaka AS2	7	-36	5UTR	High
Ciona intestinalis AS	Tunicate AS	-4	-181	5UTR[c]	High
Strongylocentrotus purpuratus AS	Purple sea urchin AS	202	-134	5UTR	High
Nematostella vectensis AS	Sea anemone AS	-68	-77	5UTR[c]	Medium
Apis mellifera AS	Honey bee AS	24	-177	5UTR	Medium
Homo sapiens AS1	Human AS1		10700	Intron9	High
Meleagris gallopavo AS1	Turkey AS1		1359	Exon9	Medium
Taeniopygia guttata AS1	Zebra Finch AS1		7244	Intron8	Medium
Anole Lizard AS1	Lizard AS1		301	Exon4	High
Anole Lizard AS2	Lizard AS2		6677	Intron7	High
Branchiostoma floridae AS	Amphioxus AS		-5013	Intron1	High
Ciona intestinalis AS	Tunicate AS		9890	Intron10	Medium

Strongylocentrotus purpuratus AS	Purple sea urchin AS	564	Intron2	Medium

[a]The localization of potential IRE in 5UTR, exon, intron, or exon-intron boundary.

[b]The distance to ATG codon does not include the intron sequence if IRE is in exon region.

[c]5'UTR is from genomic sequence.

[d]Quality of IRE as determined by SIRE.

Table S3. Potential IRE in PBGS

Species	common name	Distance to start codon (bases)[a]	Location[b]	Quality[c]
Homo sapiens	Human	4165	Intron10	Medium
Macaca mulatta	Rhesus monkey	2927	Intron7	Medium
Macaca mulatta	Rhesus monkey	3886	Intron10	Medium
Bos taurus	Cattle	1375	Intron3	Medium
Mus musculus	Mouse	-2129	Intron1	Medium
Mus musculus	Mouse	924	Exon11&Intron11	Medium
Gallus gallus	Chicken	5910	Intron7	Medium
Gallus gallus	Chicken	7039	Intron10	Medium
Meleagris gallopavo	Turkey	983	Intron3	Medium
Meleagris gallopavo	Turkey	3323	Intron7	Medium
Oryzias latipes	Medaka	4947	Intron11	High
Acropora digitifera	Coral	7915	Intron8	Medium

[a]The distance to ATG codon does not include the intron sequence if IRE is in exon region.

[b]The localization of potential IRE in 5UTR, exon, intron, or exon-intron boundary.

[c]Quality of IRE as determined by SIRE.

Table S3. Potential IRE in PBGD

Species	common name	Distance to transcription start site (bases)	Distance to start codon (bases)[a]	Location[b]	Quality[c]
Strongylocentrotus purpuratus	Purple sea urchin	140	-10	5UTR	Medium
Drosophila melanogaster	D. melanogaster	45	-319	5UTR	High
Apis mellifera	Honey bee	96	-287	5UTR	High
Bos taurus	Cattle		2367	Intron1	Medium
Loxodonta africana	Elephant		773	Intron1	Medium
Loxodonta africana	Elephant		419	Exon7&Intron7	Medium
Oryctolagus cuniculus	Rabbit		350	Exon7&Intron7	Medium
Danio rerio	Zebrafish		2500	Exon14(3UTR)	Medium
Hydra magnipapillata	Hydra		4596	Intron2	Medium

[a]The distance to ATG codon does not include the intron sequence if IRE is in exon region.

[b]The localization of potential IRE in 5UTR, exon, intron, or exon-intron boundary.

[c]Quality of IRE as determined by SIRE.

Table S3. Potential IRE in UROS

Species	common name	Distance to start codon (bases)	Location[a]	Quality[b]
Homo sapiens	Human	7567	Intron5	Medium
Macaca mulatta	Rhes monkey	8506	Intron6	Medium
Macaca mulatta	Rhes monkey	19869	Intron8	Medium
Macaca mulatta	Rhes monkey	20403	Intron8	Medium
Loxodonta africana	Elephant	1278	Intron2	Medium
Loxodonta africana	Elephant	1278	Intron2	Medium
Loxodonta africana	Elephant	10367	Intron4&Exon5	Medium
Oryctolagus cuniculus	Rabbit	11648	Intron4	Medium
Mus musculus	Mouse	3178	Intron4&Exon5	Medium
Mus musculus	Mouse	14727	Intron9	Medium
Gallus gallus	Chicken	-2160	Intron1	Medium
Anole Lizard	Lizard	2405	Intron1	Medium
Xenopus (Silurana) tropicalis	Western clawed frog	-2712	Intron1	Medium

[a]The localization of potential IRE in 5UTR, exon, intron, or exon-intron boundary.

[b]Quality of IRE as determined by SIRE.

Table S3. Potential IRE in PPO

Species	common name	Distance to start codon (bases)[a]	Location[b]	Quality[c]
Anole Lizard	Lizard	6294	Intron6	Medium
Anole Lizard	Lizard	6294	Intron6	Medium
Acropora digitifera	Coral	1265	Exon3	Medium
Nematostella vectensis	Starlet sea anemone	4301	Intron3	Medium
Hydra magnipapillata	Hydra	860	Exon4	Medium
Drosophila ananassae	D. ananassae	104	Exon1	Medium

[a]The distance to ATG codon does not include the intron sequence if IRE is in exon region.

[b]The localization of potential IRE in 5UTR, exon, intron, or exon-intron boundary.

[c]Quality of IRE as determined by SIRE.

Table S3. Potential IRE in FECH

Species	common name	Distance to start codon (bases)[a]	Location[b]	Quality[c]
Homo sapiens	Human	21711	Intron5	Medium
Homo sapiens	Human	28439	Intron7	Medium
Macaca mulatta	Rhesus monkey	12939	Intron2	High
Macaca mulatta	Rhesus monkey	10376	Intron2	Medium
Macaca mulatta	Rhesus monkey	2050	Exon11	Medium
Loxodonta africana	Elephant	22027	Intron6	High
Loxodonta africana	Elephant	24628	Intron7	Medium
Loxodonta africana	Elephant	26232	Intron7	Medium
Oryctolagus cuniculus	Rabbit	24649	Intron5	High
Oryctolagus cuniculus	Rabbit	5324	Intron1	Medium
Mus musculus	Mouse	17087	Intron4	High

[a]The distance to ATG codon does not include the intron sequence if IRE is in exon region.

[b]The localization of potential IRE in 5UTR, exon, intron, or exon-intron boundary.

[c]Quality of IRE as determined by SIRE.
