# Supplementary material for: Molecular Evolution of Multiple-Level Control of Heme Biosynthesis Pathway in Animal Kingdom
Source: PLoS One. 2014 Jan 28;9(1):e86718. doi: 10.1371/journal.pone.0086718 (PMC3904948; doi:10.1371/journal.pone.0086718)
Supplement: Table S4 — Genomewide detection of potential IRE in exon-intron boundary in human and zebrafish genes. (PDF) [file pone.0086718.s007.pdf]

Table S4. Genomewide detection of potential IRE in exon-intron boundary in human genes.

chromosome[a]	Ensembl Id[b]	GeneID[c]	Symbol[d]	Location[f]	Length of potential exon skipping (bases)[g]	Frameshift[h]	Total exon number[i]	Strand[j]	Coordinate[k]	Quality[m]
4	ENSG00000047365	116984	ARAP2	I21-E22	148	Y	32	-1	36122948	High
1	ENSG00000058673	9877	ZC3H11A	I12-E13	99	N	20	1	203807094	High
6	ENSG00000079689	10590	SCGN	I9-E10	69	N	11	1	25691284	High
19	ENSG00000083838	55663	ZNF446	I4-E5	85	Y	7	1	58991010	High
1	ENSG00000116991	57568	SIPA1L2	I6-E7	577	Y	21	-1	232601162	High
16	ENSG00000124074	84080	ENKD1	E6-I6	137	Y	7	-1	67697323	High
19	ENSG00000125755	8189	SYMPK	E2-I2	117	N	26	-1	46357649	High
3	ENSG00000133657	79572	ATP13A3	E30-I30	81	N	31	-1	194134488	High
1	ENSG00000134250	4853	NOTCH2	E17-I17	153	N	34	-1	120491037	High
12	ENSG00000135655	9958	USP15	E20-I20	104	Y	22	1	62790178	High
10	ENSG00000138160	3832	KIF11	E15-I15	126	N	22	1	94397066	High
1	ENSG00000143217	81607	PVRL4	I6-E7	76	Y	9	-1	161043585	High
10	ENSG00000150867	5305	PIP4K2	I2-E3	97	Y	10	-1	22896952	High
3	ENSG00000157445	55799	CACNA2D	E25-I25	98	Y	38	1	54919613	High
12	ENSG00000161835	160622	GRASP	E2-I2	50	Y	8	1	52403023	High
3	ENSG00000163933	91869	RFT1	E4-I4	190	Y	13	-1	53156390	High
7	ENSG00000164880	26173	INTS1	I4-E5	138	N	47	-1	1539657	High
9	ENSG00000165802	26012	NELF	I8-E9	125	Y	16	-1	140347632	High
14	ENSG00000184916	3714	JAG2	E15-I15	114	N	26	-1	105615082	High
10	ENSG00000197321	6840	SVIL	E23-I23	244	Y	35	-1	29813401	High
1	ENSG00000243710	149465	WDR65	E6-I6	153	N	10	1	43652530	High

[a]Chromosome ID for human genome.

[b]Ensembl gene Id.

[c]GeneID.

[d]Gene symbol.

[e]Gene name.

[f]Location of exon-intron boundary.

[g]Length of potential exon skipping (bases).

[h]Frameshift or stop codon because of exon skipping Y: yes, N: no.

[i]Total exon number of the gene.

[j]Transcribing strand of the gene.

[k]Coordinate of exon-intron boundary.

[l]Sequence of the exon-intron boundary sequence.

[m]Quality of IRE as determined by SIRE.

Table S4. Genomewide detection of potential IRE in exon-intron boundary in zebrafish genes.

chromosome[a]	Ensembl Id[b]	GeneID[c]	Symbol[d]	Location[f]	Length of potential exon skipping (bases)[g]	Frameshift[h]	Total exon number[i]	Strand[j]	Coordinate[k]	Quality[m]
4	ENSDARG00000070477	403080	dnajc2	I1-E2	191	Y	17	-1	1643408	High
5	ENSDARG00000021735	555941	cacna1ba	E6-I6	188	Y	49	-1	31061415	High
8	ENSDARG00000020131	445240	fnbp1l	I9-E10	95	Y	12	1	15589179	High
8	ENSDARG00000075375	571061	zgc:171501	I12-E13	177	N	14	-1	51434721	High
8	ENSDARG00000007560	1E+08	sema3bl	E13-I13	134	Y	14	1	27424224	High
9	ENSDARG00000056112	337690	pikfyve	I5-E6	172	Y	34	1	33113934	High
13	ENSDARG00000028748	406698	tm9sf3	E12-I12	147	Y	15	-1	9224136	High
13	ENSDARG00000013095	326857	gclcp	E7-I7	75	N	16	1	2159869	High
15	ENSDARG00000026753	556582	grik4	E11-I11	114	N	18	1	22505093	High
18	ENSDARG00000027423	245701	igf1ra	I9-E10	230	Y	15	-1	20678823	High
21	ENSDARG00000069467	569554	LOC569554	E11-I11	162	N	17	-1	24003472	High
25	ENSDARG00000058015	553392	ano3	E6-I6	135	N	16	1	36501443	High

[a]Chromosome ID for zebrafish genome.

[b]Ensembl gene Id.

[c]GeneID.

[d]Gene symbol.

[e]Gene name.

[f]Location of exon-intron boundary.

[g]Length of potential exon skipping (bases).

[h]Frameshift or stop codon because of exon skipping Y: yes, N: no.

[i]Total exon number of the gene.

[j]Transcribing strand of the gene.

[k]Coordinate of exon-intron boundary.

[l]Sequence of the exon-intron boundary sequence.

[m]Quality of IRE as determined by SIRE.
