# Supplementary material for: Molecular Evolution of Multiple-Level Control of Heme Biosynthesis Pathway in Animal Kingdom
Source: PLoS One. 2014 Jan 28;9(1):e86718. doi: 10.1371/journal.pone.0086718 (PMC3904948; doi:10.1371/journal.pone.0086718)
Supplement: Table S5 — Potential HRM in eight genes of heme biosynthesis pathway (HRM_t and HRM_r). (PDF) [file pone.0086718.s008.pdf]

Table S5. Potential HRM in ALAS (HRM_t and HRM_r).

**Species**	**common name**	**Distance to start codon for HRM (amino acids)**[a]					
		HRM1_t	HRM2_t	HRM3_t	HRM1_r	HRM2_r	HRM3_r
Homo sapiens AS1	Human AS1	7	32	107			
Macaca mulatta AS1	Rhesus monkey AS1	7	32	107			
Canis lupus familiaris AS1	Dog AS1	7	32	107			
Bos taurus AS1	Cattle AS1	7	32	114			
Mus musculus AS1	House mouse AS1	7	32	109			
Oryctolagus cuniculus AS1	Rabbit AS1	18	43	118			
Loxodonta africana AS1	Elephant AS1	7	32	107			
Gallus gallus AS1	Chicken AS1	7		100		31	
Meleagris gallopavo AS1	Turkey AS1	7		108		31	
Taeniopygia guttata AS1	Zebra Finch AS1	7		99			
Xenopus laevis AS1	African clawed frog AS1	7	32	91			
Xenopus (Silurana) tropicalis AS1	Western clawed frog AS1	7	32	107			
Anole Lizard AS1	Lizard AS1	16	41	119			
Danio rerio AS1	Zebrafish AS1	6	31	89			
Oryzias latipes AS1	Medaka AS1	7	32	122			
Takifugu rubripes AS1a	Fugu AS1a	7		104			
Takifugu rubripes AS1b	Fugu AS1b	7	33	103			
Gasterosteus aculeatus AS1a	Stickleback AS1a	7	33	87			
Gasterosteus aculeatus AS1b	Stickleback AS1b	7	32	96			
Homo sapiens AS2	Human AS2		37		9		68
Macaca mulatta AS2	Rhesus monkey AS2				9	36	68
Canis lupus familiaris AS2	Dog AS2		37		9		
Bos taurus AS2	Cattle AS2	10	37				68
Mus musculus AS2	House mouse AS2		37		9		68
Gallus gallus AS2	Chicken AS2	6	25				
Xenopus laevis AS2	African clawed frog AS2		32				
Xenopus (Silurana) tropicalis AS2	Western clawed frog AS2		32				

Anole Lizard AS2	Lizard AS2				
Danio rerio AS2	Zebrafish AS2				75
Oryzias latipes AS2	Medaka AS2				
Takifugu rubripes AS2	Fugu AS2				
Tetraodon nigroviridis AS	Tetraodon AS				
Gasterosteus aculeatus AS2	Stickleback AS2				
Myxine glutinosa AS	Atlantic hagfish AS	50	75	115	
Branchiostoma floridae AS	Amphioxus AS	8	33	83	
Ciona intestinalis AS	Tunicate AS	7	32	100	
Strongylocentrotus purpuratus AS	Purple sea urchin AS	8	33		
Strongylocentrotus droebachiensis	Green sea urchin	8	33	66	
Nematostella vectensis AS	Sea anemone AS	9			
Hydra magnipapillata AS	Hydra AS				
Drosophila persimilis AS	D. persimilis AS				
Drosophila melanogaster AS	D. melanogaster AS				
Drosophila ananassae AS	D. ananassae AS				
Limulus polyphemus AS	Atlantic horseshoe crab AS				
Apis mellifera AS	Honey bee AS				
Aedes aegypti AS	Y.F. mosquito AS			93	

[a]The localization of potential HRM in protein sequence. HRM were grouped to 1, 2, and 3 according to the relative position in multiple amino acid sequence alignment.

Table S5. Potential HRM in PBGS (HRM_r)

**Species**	**common name**	**Distance to start codon for HRM_r (amino acids)**
Homo sapiens	Human	122
Macaca mulatta	Rhesus monkey	129
Canis lupus familiaris	Dog	129
Bos taurus	Cattle	122
Mus musculus	Mouse	122
Danio rerio	Zebrafish	124
Gallus gallus	Chicken	129
Meleagris gallopavo	Turkey	131
Oryzias latipes	Medaka	124
Gasterosteus aculeatus	Stickleback	124
Tetraodon nigroviridis	Tetraodon	136
Oryctolagus cuniculus	Rabbit	132
Acropora digitifera	Coral	123
Xenopus (Silurana) tropicalis	Western clawed frog	68
Anole Lizard	Lizard	126
Takifugu rubripes	Fugu	131
Xenopus laevis	African clawed frog	122
Culex quinquefasciatus	S.H. mosquito	121
Aedes aegypti	Y.F. mosquito	122

Table S5. Potential HRM in PBGD (HRM_t and HRM_r)

**Species**	**common name**	**Distance to start codon for HRM_t (amino acids)**	**Distance to start codon for HRM_r (amino acids)**
Drosophila melanogaster	D. melanogaster	567	382, 540
Drosophila ananassae	D. ananassae	563	535
Drosophila persimilis	D. persimilis	457	432
