# Supplementary material for: Molecular Evolution of Multiple-Level Control of Heme Biosynthesis Pathway in Animal Kingdom
Source: PLoS One. 2014 Jan 28;9(1):e86718. doi: 10.1371/journal.pone.0086718 (PMC3904948; doi:10.1371/journal.pone.0086718)
Supplement: Table S6 — Length of evolutionarily conserved DNase-hypersensitive sites in intron sequences (bps) for Abcg2 , Ank1 and Slc11a2 . (PDF) [file pone.0086718.s009.pdf]

Table S6. Accession number for genes of heme biosynthesis pathway

Protein	Species	Accession No.	sequence code
ALAS	*Homo sapiens AS1*	NP_000679.1	HsAS1
ALAS	*Macaca mulatta AS1*	XP_001090440.2	MmuAS1
ALAS	*Canis lupus familiaris AS1*	XP_533804.2	ClfAS1
ALAS	*Bos taurus AS1*	NP_001094624.1	BtAS1
ALAS	*Mus musculus AS1*	NP_065584.2	MmAS1
ALAS	*Oryctolagus cuniculus AS1*	ENSOCUP00000007390	OcAS1
ALAS	*Loxodonta africana AS1*	ENSLAFP00000002492	LaAS1
ALAS	*Gallus gallus AS1*	NP_001018012.1	GgAS1
ALAS	*Meleagris gallopavo AS1*	ENSMGAP00000002874	MgaAS
ALAS	*Taeniopygia guttata AS1*	ENSTGUP00000006254	TgAS1
ALAS	*Xenopus laevis AS1*	NP_001086051.1	XlAS1
ALAS	*Xenopus tropicalis AS1*	NP_001008072.1	XtAS1
ALAS	*Anolis carolinensis AS1*	ENSACAP00000006616	AlAS1
ALAS	*Danio rerio AS1*	NP_958444.1	DrAS1
ALAS	*Oryzias latipes AS1*	ENSORLP00000006029	OlAS1
ALAS	*Takifugu rubripes AS1a*	ENSTRUP00000020582	TrAS1a
ALAS	*Takifugu rubripes AS1b*	ENSTRUP00000011460	TrAS1b
ALAS	*Gasterosteus aculeatus AS1a*	ENSGACP00000009281	GaAS1a
ALAS	*Gasterosteus aculeatus AS1b*	ENSGACP00000019967	GaAS1b
ALAS	*Tetraodon nigroviridis AS1*	GenBank: CAF94336.1	TnAS
ALAS	*Myxine glutinosa AS*	GenBank: AAD20810.1	MgAS
ALAS	*Ciona intestinalis AS*	XP_002125767.10	CiAS
ALAS	*Branchiostoma floridae AS*	scaffold_456:218439-229437	BfAS
ALAS	*Strongylocentrotus purpuratus AS*	GLEAN3_24016 1.00	SpAS

ALAS	*Strongylocentrotus droebachiensis*	GenBank: AAD20809.1	SdAS
ALAS	*Homo sapiens AS2*	NP_000023.2	HsAS2
ALAS	*Macaca mulatta AS2*	XP_002806298.1	MmuAS2
ALAS	*Canis lupus familiaris AS2*	XP_548619.3	ClfAS2
ALAS	*Bos taurus AS2*	NP_001030275.1	BtAS2
ALAS	*Mus musculus AS2*	NP_001095916.1	MmAS2
ALAS	*Gallus gallus AS2*	GenBank: AAA20400.1	GgAS2
ALAS	*Xenopus laevis AS2*	NP_001087499.1	XlAS2
ALAS	*Xenopus tropicalis AS2*	NP_001006926.1	XtAS2
ALAS	*Anolis carolinensis AS2*	ENSACAP00000010965	AlAS2
ALAS	*Danio rerio AS2*	NP_571757.1	DrAS2
ALAS	*Oryzias latipes AS2*	ENSORLP00000005923	OlAS2
ALAS	*Takifugu rubripes AS2*	ENSTRUP00000036168	TrAS2
ALAS	*Gasterosteus aculeatus AS2*	ENSGACP00000009047	GaAS2
ALAS	*Drosophila melanogaster AS*	GenBank: CAA74915.1	DmAS
ALAS	*Drosophila ananassae AS*	XP_001959451.1	DaAS
ALAS	*Drosophila persimilis AS*	XP_002016548.1	DpAS
ALAS	*Limulus polyphemus AS*	GenBank: AAD20805.1	LpAS
ALAS	*Apis mellifera AS*	XP_624475.1	AmAS
ALAS	*Aedes aegypti AS*	XP_001658653.1	AaAS
ALAS	*Nematostella vectensis AS*	scaffold_23:604784-619599	NvAS
ALAS	*Hydra magnipapillata AS*	XP_002154275.1	HmAS
PBGS	*Homo sapiens*	NP_000022.3	HsPS
PBGS	*Macaca mulatta*	XP_001101576.1	MmuPS
PBGS	*Canis lupus familiaris*	XP_855370.1	ClfPS

PBGS	*Bos taurus*	NP_001014895.1	BtPS
PBGS	*Mus musculus*	NP_032551.3	MmPS
PBGS	*Oryctolagus cuniculus*	ENSOCUP00000005066	OcPSb
PBGS	*Gallus gallus*	ENSGALP00000014392	GgPS
PBGS	*Meleagris gallopavo*	ENSMGAP00000001355	MgPS
PBGS	*Xenopus laevis*	NP_001085993.1	XlPS
PBGS	*Xenopus tropicalis*	NP_001072186.1	XtPS
PBGS	*Anolis carolinensis*	ENSACAP00000001880	AlPS
PBGS	*Danio rerio*	NP_001017645.1	DrPS
PBGS	*Oryzias latipes*	ENSORLP00000017232	OlPS
PBGS	*Takifugu rubripes*	ENSTRUP00000026736	TrPS
PBGS	*Gasterosteus aculeatus*	ENSGACP00000023850	GaPS
PBGS	*Tetraodon nigroviridis*	ENSTNIP00000001185	TnPS
PBGS	*Ciona intestinalis*	XP_002128180.1	CiPS
PBGS	*Drosophila melanogaster*	NP_648564.1	DmPS
PBGS	*Drosophila ananassae*	XP_001956008.1	DaPS
PBGS	*Drosophila persimilis*	XP_002021111.1	DpPS
PBGS	*Apis mellifera*	XP_397149.2	AmPS
PBGS	*Culex quinquefasciatus*	CPIJ006003-PA	CqPS
PBGS	*Aedes aegypti*	XP_001661237.1	AaPS
PBGS	*Acropora digitifera*	aug_v2a.08821	PSPS
PBGS	Nematostella vectensis	NEMVEDRAFT_v1g171543-PA	NvPS
PBGS	*Hydra magnipapillata*	XP_002162848.1	HmPS
PBGD	*Homo sapiens*	NP_000181.2	HsPD
PBGD	*Macaca mulatta*	XP_001101850.1	MmuPD

PBGD	*Canis lupus familiaris*	XP_546491.2	ClfPD
PBGD	*Bos taurus*	NP_001039672.1	BtPD
PBGD	*Mus musculus*	NP_001103721.1	MmPD
PBGD	*Oryctolagus cuniculus*	ENSOCUP00000013032	OcPD
PBGD	*Loxodonta africana*	ENSLAFP00000015573	LaPD
PBGD	*Gallus gallus*	XP_417846.2	GgPD
PBGD	*Meleagris gallopavo*	ENSMGAP00000003123	MgPD
PBGD	*Taeniopygia guttata*	ENSTGUP00000000014	TgPD
PBGD	*Xenopus laevis*	NP_001089699.1	XlPD
PBGD	*Xenopus tropicalis*	NP_001005635.1	XtPD
PBGD	*Anolis carolinensis*	ENSACAP00000015320	AlPD
PBGD	*Danio rerio*	NP_957448.1	DrPDa
PBGD	*Danio rerio*	NP_001019559.1	DrPDb
PBGD	*Oryzias latipes*	ENSORLP00000019210	OlPD
PBGD	*Takifugu rubripes*	ENSTRUP00000025487	TrPD
PBGD	*Gasterosteus aculeatus*	ENSGACP00000006841	GaPD
PBGD	*Tetraodon nigroviridis*	ENSTNIP00000017380	TnPD2
PBGD	*Ciona intestinalis*	XP_002126885.1	CiPD
PBGD	*Strongylocentrotus purpuratus*	XP_781025.1	SpPD
PBGD	*Drosophila melanogaster*	NP_612103.1	DmPD
PBGD	*Drosophila ananassae*	XP_001956689.1	DaPD
PBGD	*Drosophila persimilis*	XP_002026151.1	DpPD
PBGD	*Apis mellifera*	GB15699-PA	AmePD
PBGD	*Culex quinquefasciatus*	XP_001849943.1	CqPD
PBGD	*Aedes aegypti*	XP_001654431.1	AaPD

PBGD	*Nematostella vectensis*	gw.397.4.1(11360)	NvPD
PBGD	*Hydra magnipapillata*	XP_002156062.1	HmPD
UROS	*Homo sapiens*	NP_000366.1	HsUS
UROS	*Macaca mulatta*	XP_001086537.1	MmuUS
UROS	*Canis lupus familiaris*	XP_854192.1	ClfUS
UROS	*Bos taurus*	NP_001178298.1	BtUS
UROS	*Mus musculus*	NP_033505.1	MmUS
UROS	*Oryctolagus cuniculus*	ENSOCUP00000003943	OcUS
UROS	*Loxodonta africana*	ENSLAFP00000011106	LaUS
UROS	*Gallus gallus*	XP_423886.2	GgUS
UROS	*Meleagris gallopavo*	ENSMGAP00000012288	MgUS
UROS	*Taeniopygia guttata*	ENSTGUP00000011834	TgUS
UROS	*Xenopus laevis*	NP_001087355.1	XlUS
UROS	*Xenopus tropicalis*	NP_001107324.1	XtUS
UROS	*Anolis carolinensis*	ENSACAP00000000873	AlUS
UROS	*Danio rerio*	NP_997993.2	DrUS
UROS	*Oryzias latipes*	ENSORLP00000012228	OlUS
UROS	*Takifugu rubripes*	ENSTRUP00000023069	TrUS
UROS	*Gasterosteus aculeatus*	ENSGACP00000003420	GaUS
UROS	*Ciona intestinalis*	ENSCINP00000019540	CiUS
UROS	*Branchiostoma floridae*	XP_002608631.1	BfUS
UROS	*Strongylocentrotus purpuratus*	XP_791243.2	SpUS
UROS	*Drosophila melanogaster*	NP_572507.1	DmUS
UROS	*Drosophila ananassae*	XP_001966733.1	DaUS
UROS	*Drosophila persimilis*	XP_002022441.1	DpUS

UROS	*Culex quinquefasciatus*	CPIJ002599-PA	CqUS
UROS	*Aedes aegypti*	AAEL001978-PA	AaUS
UROS	*Acropora digitifera*	aug_v2a.08669.t1	AdUS
UROS	*Nematostella vectensis*	XP_001635247.1	NvUS
UROS	*Hydra magnipapillata*	XP_002163700.1	HmUS
UROD	*Homo sapiens*	NP_000365.3	HsUD
UROD	*Macaca mulatta*	ENSMMUP00000017069	MmuUD
UROD	*Canis lupus familiaris*	XP_532602.3	CifUD
UROD	*Bos taurus*	NP_001192487.1	BtUD
UROD	*Mus musculus*	NP_033504.2	MmUD
UROD	*Oryctolagus cuniculus*	ENSOCUP00000003606	OcUD
UROD	*Loxodonta africana*	ENSLAFP00000000773	LaUD
UROD	*Gallus gallus*	XP_422430.2	GgUD
UROD	*Meleagris gallopavo*	ENSMGAP00000010721	MgUD
UROD	*Taeniopygia guttata*	ENSTGUP00000008093	TgUD
UROD	*Xenopus laevis*	NP_001084556.1	XlUD
UROD	*Xenopus tropicalis*	NP_001011486.2	XtUD
UROD	*Anolis carolinensis*	ENSACAP00000015037	AlUD
UROD	*Danio rerio*	NP_571422.1	DrUD
UROD	*Oryzias latipes*	ENSORLP00000015888	OlUD
UROD	*Takifugu rubripes*	ENSTRUP00000008429	TrUD
UROD	*Gasterosteus aculeatus*	ENSGACP00000018965	GaUD
UROD	*Tetraodon nigroviridis*	ENSTNIP00000020757	TnUD
UROD	*Ciona intestinalis*	XP_002127816.1	CiUD
UROD	*Cion savignyi*	SINCSAVP00000009593	CsUD

UROD	*Branchiostoma floridae*	XP_002601426.1	BfUD
UROD	*Strongylocentrotus purpuratus*	XP_784308.2	SpUD
UROD	*Drosophila melanogaster*	NP_610501.1	DmUD
UROD	*Drosophila ananassae*	XP_001958625.1	DaUD
UROD	*Drosophila persimilis*	XP_002017737.1	DpUD
UROD	*Apis mellifera*	GB13137-PA	AmeUD
UROD	*Culex quinquefasciatus*	CPIJ010693-PA	CqUD
UROD	*Aedes aegypti*	AAEL006693-PB	AaUD
UROD	*Acropora digitifera*	aug_v2a.06375.t1	AdUD
UROD	*Nematostella vectensis*	NEMVEDRAFT_v1g190553-PA	NvUD
UROD	*Hydra magnipapillata*	XP_002154761.1	HmUD
CPO	*Homo sapiens*	NP_000088.3	HsCO
CPO	*Macaca mulatta*	XP_001088605.1	MmuCO
CPO	*Canis lupus familiaris*	XP_545070.3	ClfCO
CPO	*Bos taurus*	NP_001179322.1	BtCO
CPO	*Mus musculus*	NP_031783.2	MmCO
CPO	*Oryctolagus cuniculus*	ENSOCUP00000003101	OcCO
CPO	*Loxodonta africana*	ENSLAFP00000002240	LaCO
CPO	*Gallus gallus*	XP_416596.1	GgCO
CPO	*Meleagris gallopavo*	ENSMGAP00000015188	MgCO
CPO	*Taeniopygia guttata*	ENSTGUP00000013837	TgCO
CPO	*Xenopus tropicalis*	XP_002933115.1	XtCO
CPO	*Anolis carolinensis*	ENSACAP00000010659	AlCO
CPO	*Danio rerio*	NP_001035183.2	DrCO
CPO	*Oryzias latipes*	ENSORLP00000007038	OlCO

CPO	*Takifugu rubripes*	ENSTRUP00000045295	TrCO
CPO	*Gasterosteus aculeatus*	ENSGACP00000013317	GaCO
CPO	*Tetraodon nigroviridis*	ENSTNIP00000018610	TnCO
CPO	*Ciona intestinalis*	XP_002131844.1	CiCO
CPO	*Ciona savignyi*	SINCSAVP00000005156	CsCO
CPO	*Strongylocentrotus purpuratus*	XP_785975.2	SpCO
CPO	*Drosophila melanogaster*	NP_524777.1	DmCO
CPO	*Drosophila ananassae*	XP_001962390.1	DaCO
CPO	*Drosophila virilis*	XP_002052468.1	DvCO
CPO	*Apis mellifera*	GB20030-PA	AmeCO
CPO	*Culex quinquefasciatus*	CPIJ006245-PA	CqCO
CPO	*Aedes aegypti AS*	AAEL013389-PA	AaCO
CPO	*Nematostella vectensis*	XP_001633399.1	NvCO
PPO	*Homo sapiens*	NP_000300.1	HsPO
PPO	*Macaca mulatta*	XP_001117868.1	MmuPO
PPO	*Canis lupus familiaris*	XP_536137.2	ClfPO
PPO	*Bos taurus*	NP_001179355.1	BtPO
PPO	*Mus musculus*	NP_032937.1	MmPO
PPO	*Oryctolagus cuniculus*	ENSOCUP00000002076	OcPO
PPO	*Loxodonta africana*	ENSLAFP00000018153	LaPO
PPO	*Xenopus laevis*	NP_001086887.1	XlPO
PPO	*Anolis carolinensis*	ENSACAP00000003963	AlPO
PPO	*Danio rerio*	NP_001035068.1	DrPO
PPO	*Oryzias latipes*	ENSORLP00000004261	OlPO
PPO	*Takifugu rubripes*	ENSTRUP00000011985	TrPO

PPO	*Gasterosteus aculeatus*	ENSGACP00000005508	GaPO
PPO	*Tetraodon nigroviridis*	ENSTNIP00000014621	TnPO
PPO	*Ciona intestinalis*	XP_002127282.1	CiPO
PPO	*Branchiostoma floridae*	XP_002588417.1	BfPO
PPO	*Strongylocentrotus purpuratus*	XP_797442.2	SpPO
PPO	*Drosophila melanogaster*	NP_651278.2	DmPO
PPO	*Drosophila ananassae*	XP_001953418.1	DaPO
PPO	*Drosophila persimilis*	XP_002020227.1	DpPO
PPO	*Apis mellifera*	XP_003250386.1	AmPO
PPO	*Culex quinquefasciatus*	CPIJ005767-PA	CqPO
PPO	*Aedes aegypti*	AAEL003762-PA	AaPO
PPO	*Acropora digitifera*	aug_v2a.12613.t1	AdPO
PPO	*Nematostella vectensis*	XP_001633012.1	NvPO
PPO	*Hydra magnipapillata*	XP_002166439.1	HmPO
FECH	*Homo sapiens*	NP_001012533.1	HsFC
FECH	*Macaca mulatta*	XP_002800996.1	MmuFC
FECH	*Canis lupus familiaris*	XP_852936.1	ClfFC
FECH	*Bos taurus*	NP_776479.1	BtFC
FECH	*Mus musculus*	NP_032024.2	MmFC
FECH	*Oryctolagus cuniculus*	ENSOCUP00000002624	OcFC
FECH	*Loxodonta africana*	ENSLAFP00000024721	LaFC
FECH	*Gallus gallus*	NP_989527.1	GgFC
FECH	*Taeniopygia guttata*	ENSTGUP00000017780	TgFC
FECH	*Xenopus laevis*	NP_001081718.1	XlFC
FECH	*Xenopus tropicalis*	XP_002934473.1	XtFC

FECH	*Anolis carolinensis*	ENSACAP00000009322	AlFC
FECH	*Danio rerio*	NP_571706.1	DrFC
FECH	*Oryzias latipes*	ENSORLP00000018916	OlFC
FECH	*Gasterosteus aculeatus*	ENSGACP00000000840	GaFC
FECH	*Tetraodon nigroviridis*	ENSTNIP00000010061	TnFC
FECH	*Ciona intestinalis*	XP_002128123.1	CiFC
FECH	*Ciona savignyi*	SINCSAVP00000009791	CsFC
FECH	*Branchiostoma floridae*	XP_002591270.1	BfFC
FECH	*Strongylocentrotus purpuratus*	XP_787759.2	SpFC
FECH	*Drosophila melanogaster*	NP_524613.1	DmFC
FECH	*Drosophila simulans*	XP_002105667.1	DsFC
FECH	*Drosophila ananassae*	XP_001952891.1	DaFC
FECH	*Apis mellifera*	XP_393413.2	AmeFC
FECH	*Anopheles gambiae*	XP_310249.3	AgFC
FECH	*Culex quinquefasciatus*	CPIJ008006-PA	CqFC
FECH	*Aedes aegypti*	AAEL005415-PA	AaFC
FECH	*Ixodes scapularis*	ISCW016187-PA	IsFC
FECH	*Nematostella vectensis*	NEMVEDRAFT_v1g186132-PA	NvFC
FECH	*Hydra magnipapillata*	XP_002159643.1	HmFC
