# Supplementary material for: Molecular Evolution of Multiple-Level Control of Heme Biosynthesis Pathway in Animal Kingdom
Source: PLoS One. 2014 Jan 28;9(1):e86718. doi: 10.1371/journal.pone.0086718 (PMC3904948; doi:10.1371/journal.pone.0086718)
Supplement: Table S7 — IDs of genes of heme biosynthesis pathway in animals. (PDF) [file pone.0086718.s010.pdf]

Table S7. Length of evolutionarily conserved Dnase-hypersensitive sites in intron sequences (bps)

Intron[a]	*Abcg2*	*Ank1*	*Slc11a2*
1	329	999	207
2	0	147	40
3	0	0	6
4	2	0	0
5	4	0	21
6	0	176	4
7	0	0	0
8	0	0	0
9	4	110	6
10	9	0	0
11	1	87	3
12	0	0	3
13	6	0	5
14	-	89	3
15	-	0	17
16	-	220	204
17	-	0	53
18	-	0	-
19	-	0	-
20	-	100	-
21	-	112	-
22	-	2	-
23	-	74	-
24	-	0	-
25	-	0	-
26	-	0	-
27	-	0	-
28	-	0	-
29	-	0	-
30	-	40	-
31	-	0	-
32	-	0	-
32	-	8	-
33	-	0	-
34	-	0	-
35	-	0	-
36	-	0	-
37	-	0	-
38	-	0	-
39	-	14	-
40	-	356	-
41	-	43	-
42	-	83	-

[a]Intron ID.
