# Supplementary material for: Molecular Evolution of Multiple-Level Control of Heme Biosynthesis Pathway in Animal Kingdom
Source: PLoS One. 2014 Jan 28;9(1):e86718. doi: 10.1371/journal.pone.0086718 (PMC3904948; doi:10.1371/journal.pone.0086718)
Supplement: Table S8 — Number of sequences by taxonomic groups. (PDF) [file pone.0086718.s011.pdf]

Table S8. The number of sequences by taxonomic groups.

Gene	Mammal	Bird	Amphibian	Reptile	Teleost	Chordate	Echinoderm	Arthropod	Cnidaria
ALAS						3	2	6	2
ALAS1	7	3	2	1	6				
ALAS2	5	1	2	1	5				
PBGS	6	2	2	1	5	1		6	3
PBGD	7	3	2	1	6	1	1	6	2
UROS	7	3	2	1	4	2	1	5	3
UROD	7	3	2	1	5	3	1	6	3
CPO	7	3	1	1	5	2	1	6	1
PPO	7	0	1	1	5	2	1	6	3
FECH	7	2	2	1	4	3	1	8	2
