# Supplementary material for: Molecular Evolution of Multiple-Level Control of Heme Biosynthesis Pathway in Animal Kingdom
Source: PLoS One. 2014 Jan 28;9(1):e86718. doi: 10.1371/journal.pone.0086718 (PMC3904948; doi:10.1371/journal.pone.0086718)
Supplement: Table S9 — Species by taxonomic groups. (PDF) [file pone.0086718.s012.pdf]

Table S9. The species by taxonomic groups.

Mammal	Bird	Amphibian	Reptile	Teleost	Chordate	Echinoderm	Arthropod	Cnidaria
Homo sapiens	*Gallus gallus*	*Xenopus laevis*	*Anolis carolinensis*	*Danio rerio*	*Ciona intestinalis*	*Strongylocentrotus purpuratus*	*Drosophila melanogaster*	*Acropora digitifera*
Macaca mulatta	*Meleagris gallopavo*	*Xenopus tropicalis*		*Oryzias latipes*	*Ciona savignyi*	*Strongylocentrotus droebachiensis*	*Drosophila simulans*	*Nematostella vectensis*
Canis lupus familiaris	*Taeniopygia guttata*			*Takifugu rubripes*	*Branchiostoma floridae*		*Drosophila ananassae*	*Hydra magnipapillata*
Bos taurus				*Gasterosteus aculeatus*			*Apis mellifera*	
Mus musculus				*Tetraodon nigroviridis*			*Anopheles gambiae*	
Oryctolagus cuniculus							*Culex quinquefasciatus*	
Loxodonta africana							*Aedes aegypti*	
							Ixodes scapularis	
